# Efficient [(NHC)Au(NTf_2_)]-catalyzed hydrohydrazidation of terminal and internal alkynes

**DOI:** 10.3762/bjoc.16.175

**Published:** 2020-08-26

**Authors:** Maximillian Heidrich, Herbert Plenio

**Affiliations:** 1 Organometallic Chemistry, Technische Universität Darmstadt, Alarich-Weiss-Str. 12, 64287 Darmstadt, Germany

**Keywords:** alkyne, gold, homogeneous catalysis, hydrohydrazidation, NHC ligand

## Abstract

The efficient hydrohydrazidation of terminal (**6a**–**r**, 18 examples, 0.1–0.2 mol % [(NHC)Au(NTf_2_)], *T* = 60 °C) and internal alkynes (**7a**–**j**, 10 examples, 0.2–0.5 mol % [(NHC)Au(NTf_2_)], *T* = 60–80 °C) utilizing a complex with a sterically demanding bispentiptycenyl-substituted NHC ligand and the benign reaction solvent anisole, is reported.

## Introduction

**Scheme 1:**
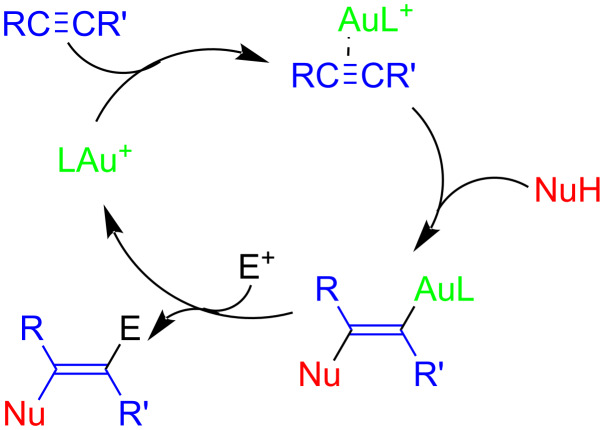
Simplified mechanism of the hydrohydrazidation (NuH= ArCONHNH_2_) of alkynes.

Cationic gold complexes with weakly coordinating counterions (which are often considered to be solvent-separated ions) [1–3], render powerful catalysts for the transformation of organic substrates [4–6], specifically the reactions of alkynes with a variety of heteroatom nucleophiles (Scheme 1) [7] such as water or alcohol [8–22], primary or secondary amines [23–28], or hydrazine [29–31]. These reactions are synthetically useful since gold catalysis is characterized by a good functional group tolerance for oxygen and nitrogen-containing molecules, which tend to be more difficult for catalytic transformations utilizing other transition metals [32–36]. The [LAu(NTf_2_)]-catalyzed reaction can be described by a general mechanism (Scheme 1), in which the coordination of LAu^+^ by the alkyne [37–38] leads to the activation of the triple bond for a nucleophilic attack, generating a vinylgold complex [39–40]. This is followed by the substitution of LAu^+^ with an electrophile E, corresponding (for E = H^+^) to a protodeauration and is leading to the release of the desired organic product [41]. The activity of gold complexes in such reactions tends to be higher, when using sterically demanding ligands (NHC, phosphine) [41].

Recently, the hydrohydrazidation of substituted phenylacetylenes using [(Ph_3_P)Au(NTf_2_)] as the catalyst was reported by Rassadin, Kukushkin et al. [42]. Catalyst loadings of 6 mol % at 60 °C were required to obtain the respective addition product in yields of 66–93%. Internal alkynes were much less reactive and even at 12 mol % catalyst loading the reaction of 3-hexyne and PhCONHNH_2_ yielded the respective addition product in only 32% yield, with diphenylacetylene only 12% of the addition product were obtained. We recently reported the excellent activity of the very bulky bispentiptycenyl-substituted (NHC)Au complexes in the hydration of terminal and internal alkynes [43] as well as in other transition-metal-catalyzed transformations [44–45]. We were now interested, whether catalysts with such ligands also display high activities in other gold-catalyzed reactions.

## Results and Discussion

**Scheme 2:**
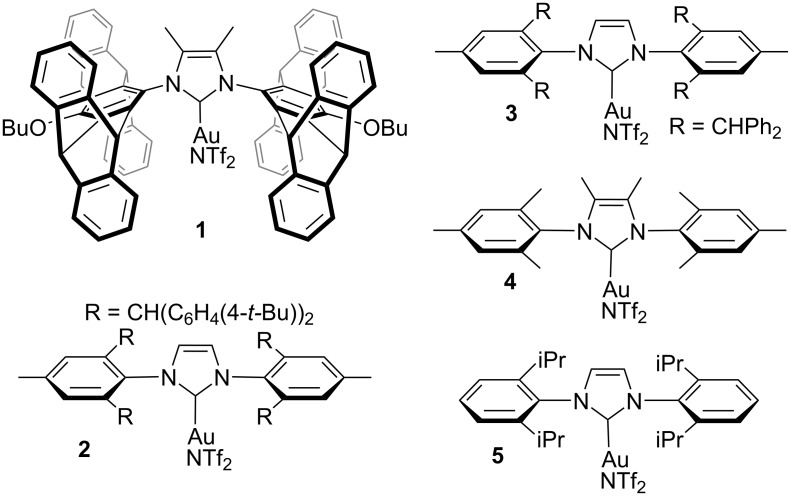
[(NHC)Au(NTf_2_)] complexes tested in hydrohydrazidation reactions of phenylacetylene.

**Catalyst screening:** Five different [(NHC)Au(NTf_2_)] complexes **1**, **2**, **3**, **4**, and **5** with bulky NHC ligands were tested in the hydrohydrazidation of phenylacetylene with benzohydrazide (Scheme 2; *T* = 60 °C, solvent chlorobenzene, reaction time 24 h, catalyst loading 0.2 mol %). The following conversions were observed: complex **1** >99%, complex **2** 16%, complex **3** 38%, complex **4** 31%, and complex **5** 60%. Complex **1** having a bispentiptycene-NHC ligand was the clear best among the five NHC-gold complexes. In this group of gold complexes with sterically demanding NHC ligands, the NHC ligands with intermediate bulk provided the best catalytic performance. The use of the bulkiest complex **2** [46], resulted only in modest substrate conversion. Nonetheless, all of the tested complexes performed much better in hydrohydrazidation reactions than the previously reported [(Ph_3_P)Au(NTf_2_)] [42].

**Scheme 3:**
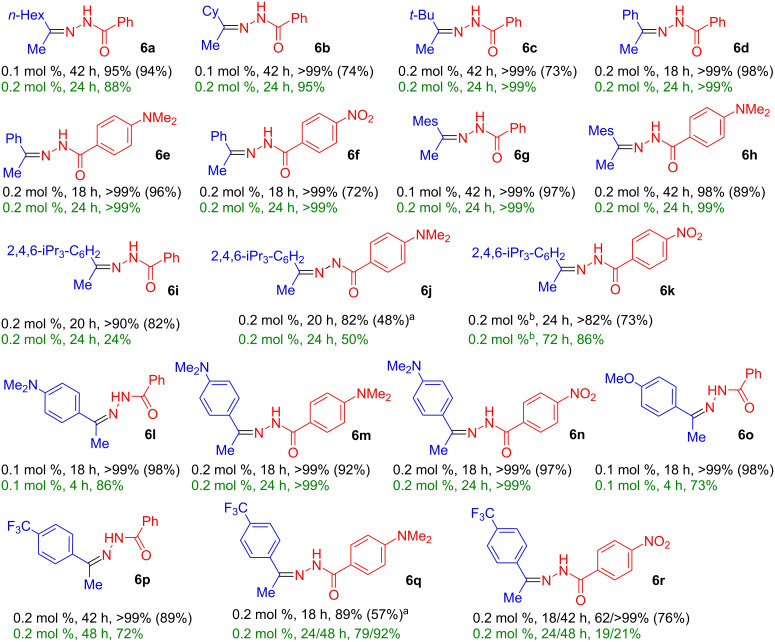
Hydrohydrazidation of terminal alkynes in chlorobenzene and anisole using complex **1** (first line solvent chlorobenzene, second line solvent anisole: catalyst loading, reaction time, conversion % (isolated yield %), reaction temperature 60 °C). ^a^Partial decomposition during column chromatography observed.

**Scheme 4:**
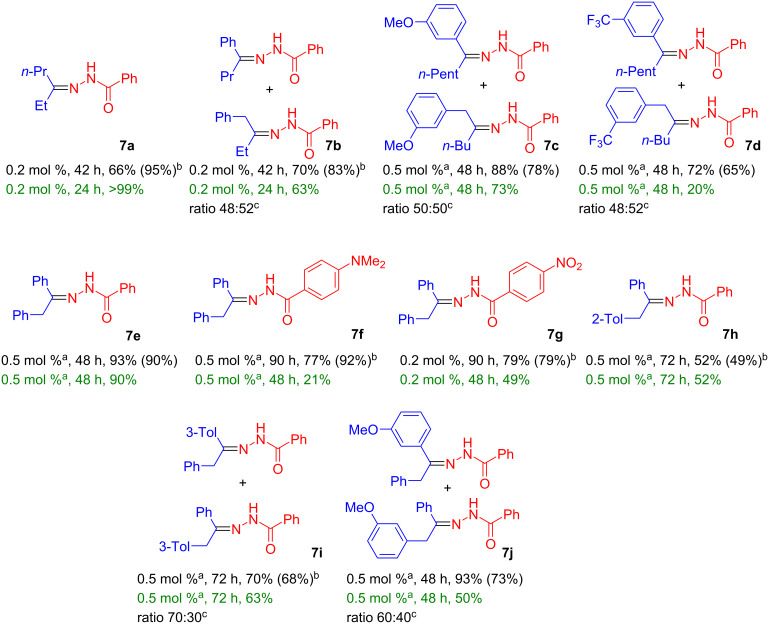
Hydrohydrazidation of internal alkynes in chlorobenzene and anisole using complex **1**. Reaction temperature was 60 °C unless otherwise noted (first line solvent chlorobenzene, second line solvent anisole: catalyst loading, reaction time, conversion % (isolated yield %). ^a^80 °C reaction temperature; ^b^isolated yield after 1 w; ^c^isomeric ratio determined via NMR spectroscopy.

**Solvent screening:** Among the solvents tested in the previous study by Rassadin, Kukushkin et al., chlorobenzene was found to be the best solvent concerning the catalytic activity [42]. However, according to the CHEM21 consortium chlorobenzene is a problematic solvent, whose use should be avoided [47]. We therefore performed an extensive solvent screening focusing on “greener” solvents for the reactions of benzohydrazide with either phenylacetylene or 4-methoxyphenylacetylene (Table 1). It was found that the catalytic performance in anisole was comparable to that in chlorobenzene. According to both the CHEM21 consortium and the GSK solvent sustainability guide the use of anisole is highly favorable [48]. To clarify the role of chlorobenzene and anisole, most substrate screening reactions were performed in both solvents (see also Scheme 3 and Scheme 4) and based on this the use of anisole as a reaction solvent could be recommended.

**Table 1:** Solvent screening for two hydrohydrazidation reactions using complex **1**.

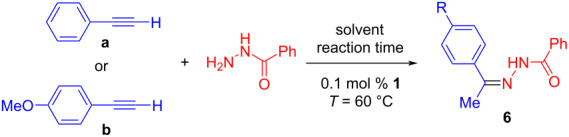				

product	solvent	time (h)	conversion (%)^a^	

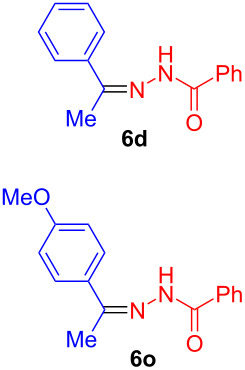	PhCl	24^b^, 6^c^	81^b^	>99^c^
	anisole	24^b^, 6^c^	80^b^	>99^c^
	xylene	24^b^	46^b^	
	MeNO_2_	24^b^, 6^c^	45^b^	82^c^
	dioxane	24^b^, 6^c^	31^b^	67^c^
	MeCN	6^c^		64^c^
	THF	6^c^		53^c^
	glyme	6^c^		68^c^

^a^GC-based conversion (internal standard mesitylene); ^b^substrate **a**; ^c^substrate **b**.

**Reaction time vs catalyst loading:** In order to optimize catalyst loading and reaction times the hydrohydrazidation of phenylacetylene was carried out at successively lower catalyst loading (Table 2). The obvious consequence being a decrease in the substrate conversion.

**Table 2:** Catalyst loading vs. reaction time in the hydrohydrazidation.

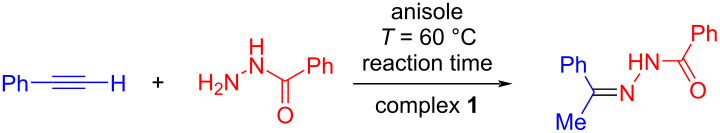			

entry	complex **1** (mol %)	time (h)	conversion (%)^a^

1	0.2	24	>99
2	0.1	24/48	79/93
3	0.05	24/48/96	51/70/>99
4	0.02	24/48	22/29
5	0.01	24/48	2/4
6	zero	24	0

^a^GC-based conversion (internal standard mesitylene).

This, however, could be compensated to some extent by longer reaction times. Based on this observation catalyst decomposition appeared to be negligible and it seemed, that the role of gold is primarily that of a Lewis acid activating the alkyne. Consequently, even at 0.05 mol % catalyst loading virtually quantitative substrate conversion was observed after extending the reaction time to 96 h (Table 2, entry 3). In the absence of the Au catalyst, no product was formed.

**Hydrohydrazidation reactions of terminal alkynes:** Following the initial optimization, a variety of hydrohydrazidation reactions of terminal and internal alkynes with benzohydrazides was studied (Scheme 3). Various combinations of alkylalkynes (1-octyne, cyclohexylacetylene, *tert*-butylacetylene), and arylalkynes (phenylacetylene, 2,4,6-trimethylphenylacetylene, 2,4,6-triisopropylphenylacetylene, and *para*-substituted 4-R-phenylacetylenes (R = NMe_2_, OMe, CF_3_)) with electronically variable benzohydrazides (4-R = NMe_2_, H, NO_2_) were tested, to cover a range of steric and electronic effects.

Most hydrohydrazidation reactions showed nearly quantitative product conversion to **6** and provided isolated yields in excess of 90% using as little as 0.1–0.2 mol % of complex **1**. Just like in related reactions, the nucleophile exclusively added to the β-carbon of the triple bond. A single substituent in the *ortho*-position of the alkyne did not exert a strong steric effect. Even the hydrohydrazidation of the sterically highly demanding 2,4,6-triisopropylphenylacetylene provided 82% isolated yield based on 90% substrate conversion (Scheme 3, compound **6i**). There was a pronounced electronic effect and electron-rich arylalkynes reacted faster than those with electron-withdrawing groups. The products derived from benzohydrazides with electron-rich aromatic groups tended to be somewhat unstable during chromatographic purification (**6j**, **6q**). In such cases it is advisable to facilitate product purification by choosing modified reaction conditions (e.g., higher catalyst loading or longer reaction times), to ensure virtually quantitative product formation.

**Hydrohydrazidation of internal alkynes:** Previously, internal alkynes could not be converted efficiently into the respective hydrohydrazidation products via gold catalysis [42], while product formation was claimed in a thermal reaction [49]. However, in the presence of complex **1** internal alkynes provided the respective addition products **7** in excellent yields using catalyst loadings in the 0.2–0.5 mol % range at slightly elevated temperature (*T* = 60–80 °C, Scheme 4). A range of dialkyl-, alkyl-, aryl-, and diarylacetylenes was reacted with electronically variable benzohydrazides (4-R = NMe_2_, H, NO_2_), to cover a range of steric and electronic substituents. In case shorter reaction times were desired, this could be compensated by increasing the amount of catalyst or the reaction temperature. The order of the alkyne reactivity in the present study was as follows: RCCH > alkyl-CC-alkyl > aryl-CC-alkyl ≥ aryl-CC-aryl.

Disubstituted acetylenes with different substituents tended to produce isomers in the hydrohydrazidation reaction (Scheme 4, products **7b**, **c**, **d**, **i**, and **j**). No preference for either isomer was observed – not even when using aryl-, alkylacetylenes, or electronically different aryl groups in tolanes. However, the steric bulk of a single methyl group in 2-methyltolane led to the selective formation of the single addition product **7h**, but even such sterically hindered tolanes (synthesized via Sonogashira coupling) [50] show reasonable substrate conversion. To assess the electronic effect of substituents on the hydrazide-substituted RC_6_H_4_-CONHNH_2_ relatives (R= NMe_2_, NO_2_) were tested leading to **7f** and **7g**. With a view to the larger distance of the functional group from the reactive center, the electronic effect was weaker, but nonetheless the conversion of the electron-deficient hydrazides (R = NO_2_) was slightly more efficient than with the electron-rich hydrazides (R = NMe_2_).

## Conclusion

In conclusion, we have demonstrated the efficient hydrohydrazidation of various terminal and internal alkynes utilizing the bispentiptycenyl-substituted [(NHC)Au(NTf_2_)] complex **1** using catalyst loadings between 0.1–0.5 mol % at temperatures between 60–80 °C in chlorobenzene, or anisole as a green reaction solvent.

## Experimental

All reagents were obtained from commercial providers (Sigma-Aldrich, Alfa Aesar, TCI Europe) and were used without further purification. All solvents were dried over CaH_2_ and distilled. All screening reactions were performed in Schlenk tubes under normal atmosphere unless otherwise noted. ^1^H and ^13^C NMR spectra were recorded at the NMR department of the Techincal University Darmstadt at 300 MHz (^1^H) and 75 MHz (^13^C) or at 500 MHz (^1^H) and 126 MHz (^13^C), respectively. Chemical shifts are given in parts per million (ppm) on the delta scale (δ) and are referred either to tetramethylsilane (^1^H; ^13^C NMR = 0 ppm) or the residual solvent peak. Thin-layer chromatography (TLC) was performed using silica gel 60 F254 (0.2 mm) on alumina plates. For preparative chromatography silica gel 60 (70–200 µm) was used. Measurements of the high resolution mass spectra (HRMS) were performed on a Bruker Daltonics Autoflex Speed TOF. Gas chromatographic data was obtained on a Clarus 500 gas chromatography system.

**Synthesis of gold complex 1**. To a solution of the (NHC)AuCl complex **1a** (60 mg, 0.043 mmol, 1 equiv) in DCM (5 mL) silver bis(trifluoromethanesulfonyl)amide (18 mg, 0.043 mmol, 1 equiv) was added and the mixture was stirred at rt for 30 min. The formed suspension was filtered through a plug of celite and the celite washed with a small amount of DCM. The volatiles of the filtrate were evaporated under reduced pressure and the residue dried in vacuo, to yield complex **1** (62 mg, 0.039 mmol, 92% yield) as white solid. ^1^H NMR (500 MHz, CD_2_Cl_2_) δ 7.54–7.48 (m, 8H), 7.46–7.39 (m, 8H), 7.08–6.93 (m, 16H), 5.90 (s, 4H), 5.19 (s, 4H), 4.12 (t, *J* = 6.8 Hz, 4H), 2.11–2.05 (m, 4H), 2.03 (s, 6H), 1.83–1.74 (m, 4H), 1.19 (td, *J* = 7.4, 1.7 Hz, 6H); ^13^C NMR (126 MHz, CD_2_Cl_2_) δ 166.41, 151.25, 146.21, 145.15, 144.71, 144.08, 142.03, 138.12, 128.93, 126.38, 125.98, 125.48, 124.67, 124.04, 123.98, 123.94, 76.83, 51.06, 48.99, 48.92, 33.13, 20.23, 14.44, 10.38; HRESIMS (*m/z*): [NHC(Au) + CH_3_CN]^+^ calcd for C_83_H_67_AuN_3_O_2_, 1334.48935; found, 1334.48923.

**General procedure for the hydrohydrazidation of alkynes.** In a small Schlenk flask equipped with a small stirring bar the corresponding alkyne (0.5 mmol) and the corresponding benzhydrazide (0.5 mmol) were mixed with anisole or chlorobenzene (2 mL). For the internal standard mesitylene (69 µL, 0.5 mmol) was added. A stock solution of the corresponding gold-triflimide catalyst (0.005 M in anisole or chlorobenzene, 200 µL, 0.001 mmol, 0.2 mol %) was introduced to the mixture. The reaction mixture was stirred at 60 °C or 80 °C for the respective time and the progress of the reaction was monitored by GC. After the reaction was completed, the solvent was removed in vacuo and the residue triturated in pentane. Column chromatography (DCM/ethyl acetate 5:1) afforded the respective benzohydrazone in good to excellent yields.

**Hydrohydrazidation of phenylacetylene with benzhydrazide on a larger scale.** In a 50 mL Schlenk flask equipped with a stirring bar phenylacetylene (5.00 mmol, 511 mg, 1 equiv) and benzhydrazide (5.00 mmol, 681 mg, 1 equiv) were mixed with anisole (20 mL). Gold-triflimide catalyst **1** (15.5 mg, 0.01 mmol, 0.2 mol %) was added and the reaction mixture was stirred at 60 °C for 24 h. The mixture was cooled to 0 °C and the formed precipitate was collected and washed with a small amount of pentane to afford N'-(1-phenylethylidene)benzohydrazide (**6d**, 1.15 g, 4.83 mmol, 97% yield) as colorless needles.

## Supporting Information

File 1 Characterization data and copies of NMR spectra and mass spectrometric data.
