# Supplementary material for: Efficient [(NHC)Au(NTf2)]-catalyzed hydrohydrazidation of terminal and internal alkynes
Source: Beilstein J Org Chem. 2020 Aug 26;16:2080–6. doi: 10.3762/bjoc.16.175 (PMC7476583; doi:10.3762/bjoc.16.175)
Supplement: File 1 — Characterization data and copies of NMR spectra and mass spectrometric data. [file Beilstein_J_Org_Chem-16-2080-s001.pdf]

BEILSTEIN JOURNAL OF ORGANIC CHEMISTRY

Supporting Information

for

Efficient [(NHC)Au(NTf_2)]-catalyzed hydrohydrazidation of terminal and internal alkynes

Maximillian Heidrich and Herbert Plenio

Beilstein J. Org. Chem. **2020,** *16,* 2080–2086. doi:10.3762/bjoc.16.175

Characterization data and copies of NMR spectra and mass spectrometric data

License and Terms: This is a supporting information file under the terms of the Creative Commons Attribution License (https://creativecommons.org/licenses/by/4.0). Please note that the reuse, redistribution and reproduction in particular requires that the authors and source are credited.
The license is subject to the *Beilstein Journal of Organic Chemistry* terms and conditions: (https://www.beilstein-journals.org/bjoc)

Table of contents

1. Experimental section S2
   1.1. Synthesis of gold complexes **2a** and **2** S2
   1.2. Terminal alkynes S4
   1.3. Internal alkynes S9
2. NMR spectra S13
   2.1. NMR spectra of complexes S13
   2.2. NMR spectra of hydrohydrazidation products S18
      2.2.1. Terminal alkynes as substrates S18
      2.2.2. Internal alkynes as substrates S34
3. Mass spectra S44
4. Gas chromatographic measurements S67
5. References S68

1. Experimental section

1.1. Synthesis of gold complexes 2a and 2

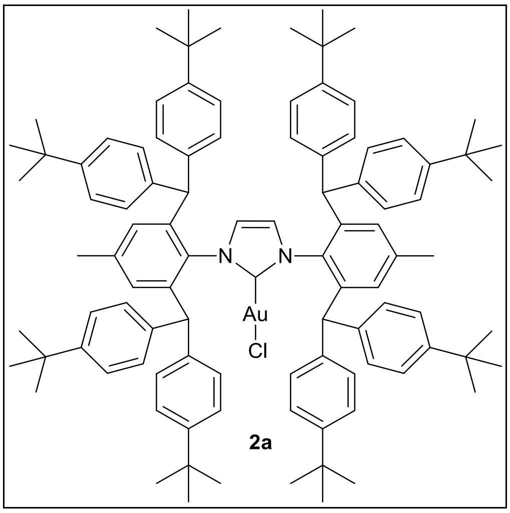

To a solution of bis(4-*tert*-butyl-diphenylmethyl)phenyl)imidazolium chloride salt (100 mg, 0.072 mmol, 1 equiv) in acetone (2 mL) [AuCl(Me_2S)] (23 mg, 0.079 mmol, 1.1 equiv) and finely powdered potassium carbonate (69 mg, 0.50 mmol, 7.0 equiv) were added. The mixture was stirred at 50 °C for 5 h. The solvent was evaporated under reduced pressure and the residue suspended in 2 mL of DCM. The suspension was filtered through a pad of celite and then the celite washed with CH_2Cl_2. The filtrate was concentrated and pentane was added. The precipitate was collected by filtration and purified via column chromatography (cyclohexane/ethyl acetate 2:1, v/v) to obtain the corresponding gold complex **2a** (77 mg, 0.048 mmol, 67% yield) as white solid. The ^{1}H NMR and ^{13}C NMR data were in accord with the literature data.[1]

^{1}H NMR (500 MHz, $CDCl_3$) δ 7.25 – 7.21 (m, 8H), 7.18 – 7.11 (m, 16H), 6.87 (s, 4H), 6.79 (d, *J* = 8.0 Hz, 8H), 5.79 (s, 2H), 5.32 (s, 4H), 2.24 (s, 6H), 1.25 (d, *J* = 2.2 Hz, 72H).

^{13}C NMR (126 MHz, $CDCl_3$) δ 174.79, 149.11, 149.04, 141.19, 139.98, 139.76, 133.66, 130.09, 129.27, 128.98, 125.23, 124.97, 123.11, 50.21, 34.36, 34.34, 31.39, 31.36, 21.88.

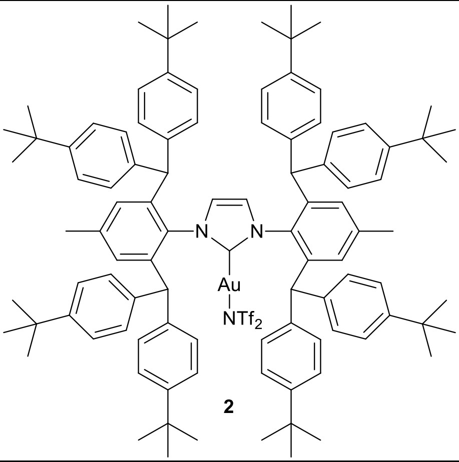

2

To a solution of the (NHC)AuCl complex **2a** (77 mg, 0.048 mmol, 1 equiv) in DCM (5 mL) silver bis(trifluoromethanesulfonyl)amide (20 mg, 0.048 mmol, 1 equiv) was added and the mixture was stirred at rt for 30 min. The formed suspension was filtered through a plug of celite and the celite washed with a small amount of DCM. The filtrate was evaporated under reduced pressure and the residue dried in vacuo to yield complex **2** (85 mg, 0.046 mmol, 96% yield) as white solid. The ^{1}H NMR and ^{13}C NMR data are in accord with the literature data.[2]
^{1}H NMR (500 MHz, $CDCl_3$) δ 7.26 (d, *J* = 8.4 Hz, 8H), 7.11 (d, *J* = 8.8 Hz, 16H), 6.87 (s, 4H), 6.74 (d, *J* = 8.3 Hz, 8H), 5.38 (s, 2H), 5.21 (s, 4H), 2.26 (s, 6H), 1.28 (s, 36H), 1.22 (s, 36H).
^{13}C NMR (126 MHz, $CDCl_3$) δ 167.61, 149.41, 141.54, 140.33, 140.11, 139.48, 133.33, 129.92, 129.54, 129.09, 125.42, 125.05, 123.44, 50.82, 34.52, 34.46, 31.50, 31.47, 21.95.

1.2. Terminal alkynes

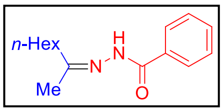

***N'*-(Octan-2-ylidene)benzohydrazide (6a)**: 1-Octyne (55 mg, 0.50 mmol) and benzhydrazide (68 mg, 0.50 mmol) were reacted and purified according to the general procedure a) for the hydrohydrazidation of alkynes to afford **6a** (115 mg, 0.47 mmol, 94% yield) as light yellow solid. The ^{1}H NMR data are in accord with the literature data.[3]

^{1}H NMR (300 MHz, DMSO-d_6): δ 10.37 (s, 1H), 7.89 – 7.67 (m, 2H), 7.64 – 7.37 (m, 3H), 2.43 – 2.16 (m, 2H), 2.03 – 1.85 (m, 3H), 1.62 – 1.38 (m, 2H), 1.38 – 1.17 (m, 6H), 0.97 – 0.76 (m, 3H).

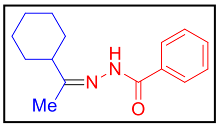

***N'*-(1-Cyclohexylethylidene)benzohydrazide (6b)**: Cyclohexylacetylene (54 mg, 0.50 mmol) and benzhydrazide (68 mg, 0.50 mmol) were reacted and purified according to the general procedure for the hydrohydrazidation of alkynes to afford **6b** (90 mg, 0.37 mmol, 74% yield) as colorless solid. The ^{1}H NMR, ^{13}C NMR and MS data are in accord with the literature data.[4]

^{1}H NMR (500 MHz, DMSO-d_6): δ 10.33 (s, 1H), 7.81 (d, J = 7.6 Hz, 2H), 7.50 (m, 3H), 2.24 (s, 1H), 1.91 (s, 3H), 1.71 (m, 5H), 1.39 – 1.15 (m, 5H).

^{13}C NMR (126 MHz, DMSO-d_6): δ 165.85, 163.16, 134.27, 131.15, 128.22, 127.57, 46.59, 29.73, 25.64, 25.57, 14.92.

HRMS (APCI): *m/z* calcd. for $C_{15}H_{21}N_2O$: 245.16484 [M+H]$^+$; found: 245.16509.

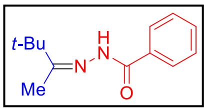

***N'*-(3,3-Dimethylbutan-2-ylidene)benzohydrazide (6c)**: *tert.*-Butylacetylene (41 mg, 0.50 mmol) and benzhydrazide (68 mg, 0.50 mmol) were reacted and purified according to the general procedure for the hydrohydrazidation of alkynes to afford **6c** (80 mg, 0.37 mmol, 73% yield) as a colorless solid. The ^{1}H NMR, ^{13}C NMR and MS data are in accord with the literature data.[5]

^{1}H NMR (300 MHz, DMSO-d_6): δ 10.29 (s, 1H), 7.82 (d, J = 7.1 Hz, 2H), 7.61 – 7.38 (m, 3H), 1.93 (s, 3H), 1.14 (s, 9H).

^{13}C NMR (75 MHz, DMSO-d_6): δ 168.26, 163.23, 134.35, 131.19, 128.27, 127.59, 38.70, 27.64, 12.88.

HRMS (APCI): *m/z* calcd. for $C_{13}H_{19}N_2O$: 219.14919 [M+H]$^+$; found: 219.14897.

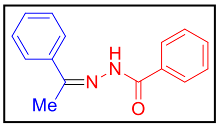

***N'*-(1-Phenylethylidene)benzohydrazide (6d):** Phenylacetylene (51 mg, 0.50 mmol) and benzhydrazide (68 mg, 0.50 mmol) were reacted and purified according to the general procedure for the hydrohydrazidation of alkynes to afford **6d** (117 mg, 0.49 mmol, 98% yield) as colorless solid. The ^{1}H NMR data are in accord with the literature data.[3]

^{1}H NMR (500 MHz, DMSO-d_6): δ 10.78 (s, 1H), 8.00 – 7.70 (m, 4H), 7.60 (t, J = 7.3 Hz, 1H), 7.53 (t, J = 7.5 Hz, 2H), 7.50 – 7.39 (m, 3H), 2.39 (s, 3H).

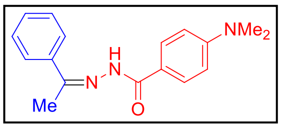

4-(Dimethylamino)-*N*'-(1-phenylethylidene)benzohydrazide (6e): Phenylacetylene (51 mg, 0.50 mmol) and 4-dimethylaminobenzhydrazide (90 mg, 0.50 mmol) were reacted and purified according to the general procedure for the hydrohydrazidation of alkynes to afford **6e** (135 mg, 0.48 mmol, 96% yield) as light yellow solid.

^{1}H NMR (500 MHz, DMSO-d_6): δ 10.37 (s, 1H), 7.82 (d, J = 6.5 Hz, 4H), 7.48 – 7.37 (m, 3H), 6.75 (d, J = 8.7 Hz, 2H), 3.00 (s, 6H), 2.35 (s, 3H).

^{13}C NMR (126 MHz, DMSO-d_6): δ 152.42, 138.40, 129.66, 129.15, 128.34, 126.28, 120.13, 110.75, 39.73, 14.21. (Because of overlapping signals, some aromatic C signals could not be observed.)

HRMS (ESI): m/z calcd. for $C_{17}H_{20}N_3O$: 282.16009 $[M+H]^+$; found: 282.15997.

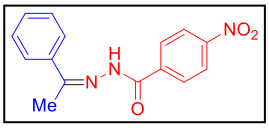

4-Nitro-*N*'-(1-phenylethylidene)benzohydrazide (6f): Phenylacetylene (51 mg, 0.50 mmol) and 4-nitrobenzhydrazide (90 mg, 0.50 mmol) were reacted and purified according to the general procedure for the hydrohydrazidation of alkynes to afford **6f** (102 mg, 0.55 mmol, 72% yield) as yellow solid. The ^{1}H NMR data are in accord with the literature data.[3]

^{1}H NMR (300 MHz, DMSO-d_6): δ 11.09 (s, 1H), 8.34 (d, J = 8.3 Hz, 2H), 8.21 – 7.26 (m, 7H), 2.39 (s, 3H).

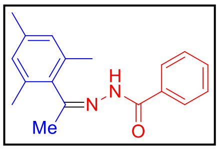

***N*'-(1-Mesitylethylidene)benzohydrazide (6g)**: Mesitylacetylene (72 mg, 0.50 mmol) and benzhydrazide (68 mg, 0.50 mmol) were reacted and purified according to the general procedure for the hydrohydrazidation of alkynes to afford **6g** (156 mg, 0.55 mmol, 97% yield) as colorless solid. The ^{1}H NMR data are in accord with the literature data.[3]

^{1}H NMR (500 MHz, DMSO-d_6): δ 9.33 (s, 1H), 7.50 (t, J = 7.3 Hz, 2H), 7.45 – 7.36 (m, 3H), 6.98 (s, 2H), 2.27 (s, 3H), 2.17 (s, 3H), 2.11 (s, 6H).

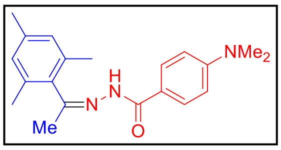

4-(Dimethylamino)-*N*'-(1-mesitylethylidene)benzohydrazide (6h): Mesitylacetylene (72 mg, 0.50 mmol) and 4-dimethylaminobenzhydrazide (90 mg, 0.50 mmol) were reacted and purified according to the general procedure for the hydrohydrazidation of alkynes to afford **6h** (144 mg, 0.45 mmol, 89% yield) as light yellow solid.

^{1}H NMR (300 MHz, DMSO-d_6): δ 10.32 (s, 1H), 7.78 (d, J = 8.8 Hz, 2H), 6.87 (s, 2H), 6.73 (d, J = 8.7 Hz, 2H), 2.99 (s, 6H), 2.24 (s, 3H), 2.17 (s, 3H), 2.16 (s, 6H).

^{13}C NMR (75 MHz, DMSO-d_6): δ 152.28, 137.43, 136.45, 134.37, 129.41, 127.88, 120.11, 110.69, 39.68, 20.60, 19.09, 18.75. (Because of low sample concentration, the amide C signal and some aromatic C signals could not be observed.)

HRMS (ESI): m/z calcd. for $C_{20}H_{26}N_3O$: 324.20704 $[M+H]^+$; found: 324.20717.

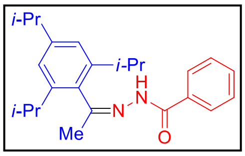

***N*'-(1-(2,4,6-Triisopropylphenyl)ethylidene)benzohydrazide (6i)**: 2,4,6-Triisopropylphenylacetylene (114 mg, 0.50 mmol) and benzhydrazide (68 mg, 0.50 mmol) were reacted and purified according to the general procedure for the hydrohydrazidation of alkynes to afford **6i** (150 mg, 0.41 mmol, 82% yield) as colorless solid.

^{1}H NMR (500 MHz, DMSO-d_6): δ 10.74 (s, 1H), 7.93 (s, 2H), 7.64 – 7.43 (m, 3H), 7.04 (s, 2H), 2.95 – 2.77 (m, 3H), 2.24 (s, 3H), 1.22 (s, 3H), 1.20 (s, 6H), 1.19 (s, 3H), 1.18 – 1.12 (m, 6H).

^{13}C NMR (126 MHz, DMSO-d_6): δ 163.12, 161.54, 148.20, 145.05, 135.53, 133.88, 131.37, 128.24, 127.78, 120.44, 33.62, 30.00, 24.51, 23.94, 23.84, 20.67.

HRMS (APCI): *m/z* calcd. for $C_{24}H_{33}N_2O$: 365.25874 [M+H]$^+$; found: 365.25903.

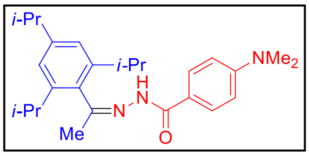

4-(Dimethylamino)-*N*'-(1-(2,4,6-triisopropylphenyl)ethylidene)-benzohydrazide: (6j): 2,4,6-Triisopropylphenylacetylene (114 mg, 0.50 mmol) and 4-dimethylaminobenzhydrazide (90 mg, 0.50 mmol) were reacted and purified according to the general procedure for the hydrohydrazidation of alkynes to afford **6j** (97 mg, 0.24 mmol, 48% yield) as colorless solid.

^{1}H NMR (500 MHz, DMSO-d_6): δ 10.33 (s, 1H), 7.79 (d, J = 8.4 Hz, 2H), 7.03 (s, 2H), 6.72 (d, J = 8.4 Hz, 2H), 2.98 (s, 6H), 2.92 – 2.81 (m, 3H), 2.21 (s, 3H), 1.21 (d, J = 6.9 Hz, 6H), 1.19 (d, J = 6.9 Hz, 6H), 1.15 (d, J = 6.8 Hz, 6H).

^{13}C NMR (126 MHz, DMSO-d_6): δ 152.24, 148.08, 145.14, 135.80, 129.43, 120.41, 120.09, 110.65, 33.62, 29.97, 24.50, 23.96, 23.88, 20.34. (Because of low sample concentration, the amide C signal and some aromatic C signals could not be observed.)

HRMS (ESI): *m/z* calcd. for $C_{26}H_{38}N_3O$: 408.30094 [M+H]$^+$; found: 408.30119.

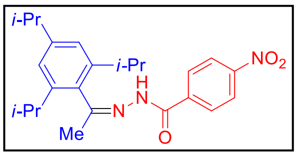

4-Nitro-*N*'-(1-(2,4,6-triisopropylphenyl)ethylidene)benzohydrazide (6k): 2,4,6-Triisopropylphenylacetylene (114 mg, 0.50 mmol) and 4-nitrobenzhydrazide (90 mg, 0.50 mmol) were reacted and purified according to the general procedure for the hydrohydrazidation of alkynes to afford **6k** (150 mg, 0.37 mmol, 73% yield) as colorless solid.

^{1}H NMR (500 MHz, DMSO-d_6) δ 11.08 (s, 1H), 8.35 (d, J = 8.3 Hz, 2H), 8.18 (d, J = 8.3 Hz, 2H), 7.05 (s, 2H), 2.95 – 2.79 (m, 3H), 2.25 (s, 3H), 1.25 – 1.14 (m, 18H).

^{13}C NMR (126 MHz, DMSO-d_6) δ 163.26, 161.52, 149.08, 148.33, 144.97, 139.60, 135.30, 129.34, 123.40, 120.49, 33.62, 30.03, 24.52, 23.92, 23.80, 20.98.

HRMS (ESI): *m/z* calcd. for $C_{24}H_{32}N_3O_3$: 410.24382 [M+H]$^+$; found: 410.24408.

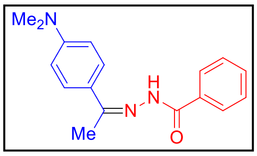

***N*'-(1-(4-(Dimethylamino)phenyl)ethylidene)benzohydrazide (6l)**: 4-Dimethylaminophenylacetylene (73 mg, 0.50 mmol) and benzhydrazide (68 mg, 0.50 mmol) were reacted and purified according to the general procedure for the hydrohydrazidation of alkynes to afford **6l** (139 mg, 0.49 mmol, 98% yield) as light yellow solid.

^{1}H NMR (500 MHz, DMSO-d_6): δ 10.60 (s, 1H), 7.88 (d, J = 7.5 Hz, 2H), 7.73 (d, J = 8.5 Hz, 2H), 7.59 – 7.53 (m, 1H), 7.50 (t, J = 7.6 Hz, 2H), 6.74 (d, J = 8.5 Hz, 2H), 2.96 (s, 6H), 2.28 (s, 3H).

^{13}C NMR (126 MHz, DMSO-d_6): δ 163.28, 156.98, 151.16, 134.32, 131.22, 128.28, 127.63, 127.59, 125.13, 111.38, 39.78, 14.22.

HRMS (APCI): *m/z* calcd. for $C_{17}H_{20}N_3O$: 282.16009 [M+H]$^+$; found: 282.16011.

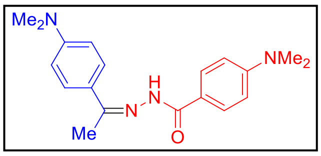

4-(Dimethylamino)-*N*'-(1-(4-(dimethylamino)phenyl)ethylidene)-benzohydrazide (6m): 4-Dimethylaminophenylacetylene (73 mg, 0.50 mmol) and 4-dimethylaminobenzhydrazide (90 mg, 0.50 mmol) were reacted and purified according to the general procedure for the hydrohydrazidation of alkynes to afford **6m** (150 mg, 0.46 mmol, 92% yield) as yellow solid.

^{1}H NMR (500 MHz, DMSO-d_6): δ 10.20 (s, 1H), 7.83 – 7.76 (m, 2H), 7.69 (d, J = 8.3 Hz, 2H), 6.77 – 6.71 (m, 4H), 2.99 (s, 7H), 2.95 (s, 6H), 2.26 (s, 3H).

^{13}C NMR (126 MHz, DMSO-d_6): δ 163.98, 153.99, 152.39, 151.14, 129.42, 127.31, 125.97, 120.95, 111.58, 110.86, 39.78, 39.66, 13.64.

HRMS (ESI): m/z calcd. for $C_{19}H_{25}N_4O$: 325.20229 $[M+H]^+$; found: 325.20241.

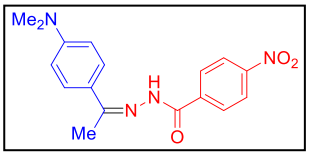

***N*'-(1-(4-(Dimethylamino)phenyl)ethylidene)-4-nitrobenzohydrazide (6n)**: 4-Dimethylphenylacetylene (73 mg, 0.50 mmol) and 4-nitrobenzhydrazide (90 mg, 0.50 mmol) were reacted and purified according to the general procedure for the hydrohydrazidation of alkynes to afford **6n** (159 mg, 0.55 mmol, 97% yield) as orange solid.

^{1}H NMR (500 MHz, DMSO-d_6): δ 11.05 – 10.88 (m, 1H), 8.36 – 8.28 (m, 3H), 8.11 (d, J = 8.3 Hz, 2H), 7.94 (d, J = 8.0 Hz, 1H), 7.74 (d, J = 8.5 Hz, 2H), 7.43 (d, J = 7.9 Hz, 1H), 6.74 (d, J = 8.5 Hz, 2H), 6.64 (d, J = 8.1 Hz, 1H), 2.97 (s, 6H), 2.90 (s, 2H), 2.30 (s, 3H), 2.26 (s, 1H).

^{13}C NMR (126 MHz, DMSO-d_6): δ 161.79, 158.28, 151.35, 148.95, 140.12, 130.48, 129.21, 127.72, 127.13, 124.78, 123.43, 122.72, 111.36, 39.76, 14.42.

HRMS (ESI): m/z calcd. for $C_{17}H_{19}N_4O_3$: 327.14517 $[M+H]^+$; found: 327.14546.

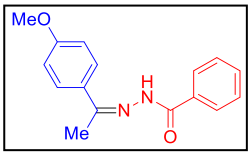

***N*'-(1-(4-Methoxyphenyl)ethylidene)benzohydrazide (6o)**: 4-Methoxyphenylacetylene (66 mg, 0.50 mmol) and benzhydrazide (68 mg, 0.50 mmol) were reacted and purified according to the general procedure for the hydrohydrazidation of alkynes to afford **6o** (131 mg, 0.49 mmol, 98% yield) as colorless solid. The ^{1}H NMR data are in accord with the literature data.[3]

^{1}H NMR (500 MHz, DMSO-d_6): δ 10.69 (s, 1H), 7.87 (d, J = 30.3 Hz, 4H), 7.71 – 7.41 (m, 4H), 7.01 (d, J = 7.9 Hz, 2H), 3.82 (s, 3H), 2.35 (s, 3H).

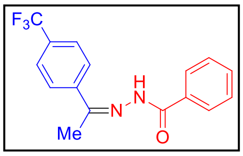

***N*'-(1-(4-(Trifluoromethyl)phenyl)ethylidene)benzohydrazide (6p)**: 4-Trifluormethylphenylacetylene (85 mg, 0.50 mmol) and benzhydrazide (68 mg, 0.50 mmol) were reacted and purified according to the general procedure for the hydrohydrazidation of alkynes to afford **6p** (136 mg, 0.44 mmol, 89% yield) as colorless solid. The ^{1}H NMR, ^{13}C NMR and MS data are in accord with the literature data.[5]

^{1}H NMR (500 MHz, DMSO-d_6): δ 10.89 (s, 1H), 8.13 – 7.96 (m, 2H), 7.93 – 7.85 (m, 2H), 7.79 (d, J = 8.0 Hz, 2H), 7.59 (t, J = 7.4 Hz, 1H), 7.52 (t, J = 7.5 Hz, 2H), 2.41 (s, 3H).

^{13}C NMR (126 MHz, DMSO-d_6): δ 164.18, 153.14, 142.00, 133.92, 131.53, 129.70 – 129.13 (m), 128.23, 127.41, 127.04, 125.24, 125.21, 123.09, 120.92, 14.40.

HRMS (APCI): m/z calcd. for $C_{16}H_{14}F_3N_2O$: 307.10527 $[M+H]^+$; found: 307.10522.

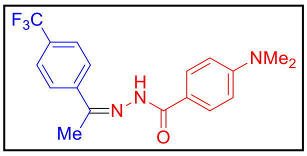

4-(Dimethylamino)-*N*'-(1-(4-(trifluoromethyl)phenyl)ethylidene)-benzohydrazide (6q): 4-Trifluormethylphenylacetylene (85 mg, 0.50 mmol) and 4-dimethylaminobenzhydrazide (90 mg, 0.50 mmol) were reacted and purified according to the general procedure for the hydrohydrazidation of alkynes to afford **6q** (99 mg, 0.28 mmol, 57% yield) as light yellow solid.
^{1}H NMR (500 MHz, DMSO-d_6): δ 10.52 (s, 1H), 8.04 (d, J = 8.1 Hz, 2H), 7.84 – 7.77 (m, 4H), 6.77 (d, J = 9.0 Hz, 2H), 3.01 (s, 6H), 2.41 (s, 3H).
^{13}C NMR (126 MHz, DMSO-d_6): δ 152.46, 142.29, 129.82, 129.09, 128.82, 128.19, 126.88, 125.22, 123.15, 119.85, 110.80, 110.68, 39.69, 14.04. (Because of overlapping signals, some aromatic C signals and signals of the CF_3 multiplet could not be observed.)
HRMS (ESI): *m/z* calcd. for $C_{18}H_{19}F_3N_3O$: 350.14747 $[M+H]^+$; found: 350.14751.

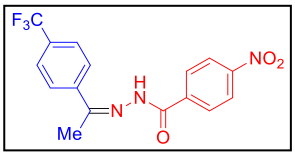

4-Nitro-*N*'-(1-(4-(trifluoromethyl)phenyl)ethylidene)benzo-hydrazide (6r): 4-Trifluormethylphenylacetylene (85 mg, 0.50 mmol) and 4-nitrobenzhydrazide (90 mg, 0.50 mmol) were reacted and purified according to the general procedure for the hydrohydrazidation of alkynes to afford **6r** (70 mg, 0.55 mmol, 76% yield) as colorless solid.
^{1}H NMR (300 MHz, DMSO-d_6): δ 11.23 (s, 1H), 8.35 (d, J = 8.3 Hz, 2H), 8.20 – 7.65 (m, 6H), 2.43 (s, 3H).
^{13}C NMR (75 MHz, DMSO-d_6): δ 162.71, 154.55, 149.21, 141.76, 139.61, 130.73 – 129.92 (m), 129.56, 127.21, 126.98, 125.95, 125.31, 125.25, 123.38, 122.34, 14.73.
HRMS (ESI): *m/z* calcd. for $C_{16}H_{13}F_3N_3O_3$: 352.09035 $[M+H]^+$; found: 352.09046.

1.3. Internal alkynes

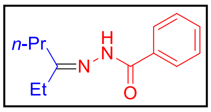

***N*'-(Hexan-3-ylidene)benzohydrazide (7a)**: 3-Hexyne (41 mg, 0.50 mmol) and benzhydrazide (68 mg, 0.50 mmol) were reacted and purified according to the general procedure for the hydrohydrazidation of alkynes to afford **7a** (104 mg, 0.48 mmol, 95% yield) as colorless solid. The ^{1}H NMR data are in accord with the literature data.[3]

^{1}H NMR (300 MHz, DMSO-d_6): δ 10.46 (s, 1H), 7.86 – 7.68 (m, 2H), 7.57 – 7.37 (m, 3H), 2.44 – 2.18 (m, 4H), 1.64 – 1.41 (m, 2H), 1.15 – 0.98 (m, 3H), 0.91 (t, *J* = 7.1 Hz, 3H).

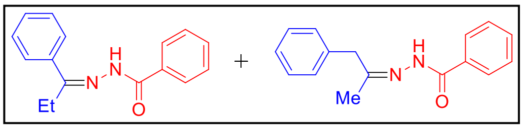

***N*'-(1-Phenylbutylidene)benzohydrazide and *N*'-(1-phenylbutan-2-ylidene)benzohydrazide (7b)**: 1-Phenyl-1-butyne (65 mg, 0.50 mmol) and benzhydrazide (68 mg, 0.50 mmol) were reacted and purified according to the general procedure for the hydrohydrazidation of alkynes to afford a mixture of **7b** (110 mg, 0.55 mmol, 83% yield, 48:52 ratio) as colorless solid.

^{1}H NMR (500 MHz, DMSO-d_6): δ 10.64 (s, 1H), 10.56 (s, 1H), 7.84 – 7.78 (m, 2H), 7.75 – 7.69 (m, 2H), 7.57 – 7.42 (m, 7H), 7.37 – 7.27 (m, 6H), 7.28 – 7.19 (m, 5H), 3.83 (s, 2H), 3.65 (s, 2H), 2.33 (q, *J* = 7.6 Hz, 2H), 2.30 – 2.20 (m, 2H), 1.11 – 1.00 (m, 3H), 0.94 (t, 4H).

^{13}C NMR (126 MHz, DMSO-d_6): δ 165.41, 163.76, 163.39, 137.31, 136.32, 134.13, 131.23, 128.92, 128.73, 128.60, 128.44, 128.22, 126.51, 126.46, 42.19, 35.69, 29.79, 22.21, 10.78, 9.75.

HRMS (APCI): *m/z* calcd. for $C_{17}H_{19}N_2O$: 267.14919 [M+H]$^+$; found: 267.14934.

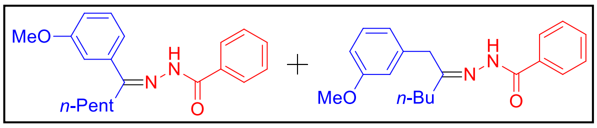

***N*'-(1-(3-Methoxyphenyl)hexylidene)benzohydrazide and *N*'-(1-(3-methoxyphenyl)-hexane-2-ylidene)benzohydrazide (7c)**: 1-(Hex-1-yn-1-yl)-3-methoxybenzene (94 mg, 0.50 mmol) and benzhydrazide (68 mg, 0.50 mmol) were reacted and purified according to the general procedure for the hydrohydrazidation of alkynes to afford a mixture of **7c** (126 mg, 0.39 mmol, 78% yield, 50:50 ratio) as pale yellow, viscous oil.

^{1}H NMR (500 MHz, DMSO-d_6): δ 10.66 (s, 1H), 10.61 (s, 1H), 7.83 – 7.72 (m, 4H), 7.58 – 7.39 (m, 8H), 7.26 – 7.21 (m, 2H), 6.88 – 6.78 (m, 5H), 3.79 (s, 3H), 3.74 (s, 3H), 3.72 (s, 4H), 2.36 – 2.28 (m, 2H), 2.24 (s, 2H), 1.49 – 1.25 (m, 8H), 0.84 – 0.79 (m, 6H).

^{13}C NMR (126 MHz, DMSO-d_6): δ 164.84, 163.33, 159.43, 159.34, 138.87, 137.75, 134.09, 131.26, 129.64, 129.43, 128.23, 127.56, 121.16, 120.91, 119.05, 114.86, 114.66, 114.52, 111.84, 54.92, 42.59, 36.14, 35.75, 31.06, 28.58, 28.09, 27.01, 25.75, 22.11, 21.87, 21.76, 13.79, 13.70, 13.59.

HRMS (EI): *m/z* calcd. for $C_{20}H_{24}N_2O_2$: 324.1832 [M]; found: 324.1823

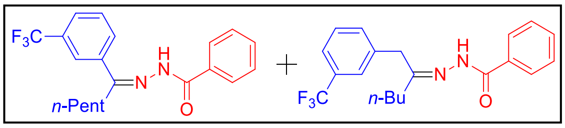

***N*'-(1-(3-(Trifluoromethyl)phenyl)hexylidene)-benzohydrazide and *N*'-(1-(3-(trifluoromethyl)-phenyl)hexane-2-ylidene)benzohydrazide (7d)**: 1-(Hex-1-yn-1-yl)-3-(trifluoromethyl)benzene (113 mg, 0.50 mmol) and benzhydrazide (68 mg, 0.50 mmol) were reacted and purified according to the general procedure for the hydrohydrazidation of alkynes to afford a mixture of **7d** (107 mg, 0.32 mmol, 65% yield, 48:52 ratio) as colorless viscous oil.

^{1}H NMR (500 MHz, DMSO-d_6): δ 10.73 (s, 1H), 10.64 (s, 1H), 7.83 – 7.38 (m, 19H), 3.94 (s, 2H), 3.81 – 3.70 (m, 2H), 2.35 (t, J = 7.9 Hz, 2H), 2.28 – 2.19 (m, 2H), 1.55 – 1.43 (m, 2H), 1.42 – 1.35 (m, 2H), 1.29 – 1.18 (m, 4H), 0.86 – 0.75 (m, 6H).

^{13}C NMR (126 MHz, DMSO-d_6): δ 164.36, 163.53, 162.52, 138.89, 137.80, 134.02, 133.24, 132.81, 131.27, 129.61, 129.37, 128.20, 127.57, 125.45, 125.35, 125.31, 123.24, 123.20, 42.03, 36.24, 35.42, 28.93, 27.99, 26.96, 22.05, 21.68, 13.61, 13.53.

HRMS (EI): *m/z* calcd. for $C_{20}H_{21}N_2OF_2$: 362.1600 [M]; found: 267.1592.

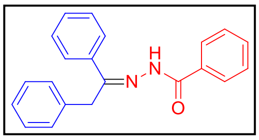

***N*'-(1,2-Diphenylethylidene)benzohydrazide (7e)**: Diphenylacetylene (89 mg, 0.50 mmol) and benzhydrazide (68 mg, 0.50 mmol) were reacted and purified according to the general procedure for the hydrohydrazidation of alkynes to afford **7e** (142 mg, 0.45 mmol, 90% yield) as colorless solid. The ^{1}H NMR data are in accord with the literature data.[3]

^{1}H NMR (500 MHz, DMSO-d_6): δ 10.99 (s, 1H), 7.86 (s, 2H), 7.74 (d, J = 7.6 Hz, 2H), 7.56 (t, J = 7.4 Hz, 1H), 7.48 (t, J = 7.6 Hz, 2H), 7.38 (s, 3H), 7.30 (t, J = 7.5 Hz, 2H), 7.26 – 7.15 (m, 3H), 4.41 (s, 2H).

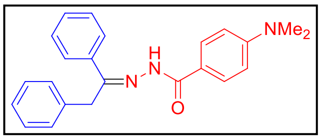

4-(Dimethylamino)-*N*'-(1,2-diphenylethylidene)benzohydrazide (7f): Diphenylacetylene (89 mg, 0.50 mmol) and 4-dimethylamino-benzhydrazide (90 mg, 0.50 mmol) were reacted and purified according to the general procedure for the hydrohydrazidation of alkynes to afford (165 mg, 0.46 mmol, 92% yield) as colorless solid.

^{1}H NMR (300 MHz, DMSO-d_6): δ 10.54 (s, 1H), 7.83 (d, J = 6.7 Hz, 2H), 7.67 (d, J = 8.4 Hz, 2H), 7.39 – 7.17 (m, 8H), 6.72 (d, J = 7.8 Hz, 2H), 4.41 (s, 2H), 2.98 (d, J = 2.0 Hz, 6H).

^{13}C NMR (75 MHz, DMSO-d_6): δ 152.39, 137.78, 136.46, 129.68, 128.97, 128.68, 128.32, 128.11, 126.61, 126.33, 119.87, 110.62, 39.65, 32.03.

HRMS (ESI): *m/z* calcd. for $C_{23}H_{24}N_3O$: 358.19139 $[M+H]^+$; found: 358.19155.

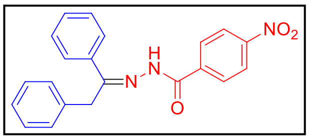

***N*'-(1,2-Diphenylethylidene)-4-nitrobenzohydrazide (7g)**: Diphenylacetylene (89 mg, 0.50 mmol) and 4-nitrobenzhydrazide (90 mg, 0.50 mmol) were reacted and purified according to the general procedure for the hydrohydrazidation of alkynes to afford **7g** (143 mg, 0.55 mmol, 79% yield) as colorless solid.

^{1}H NMR (300 MHz, DMSO-*d*$_6$): δ 11.36 (s, 1H), 8.33 (d, *J* = 8.3 Hz, 2H), 7.99 (d, *J* = 8.4 Hz, 2H), 7.91 – 7.83 (m, 1H), 7.72 – 7.46 (m, 1H), 7.46 – 7.12 (m, 8H), 4.41 (s, 2H).

^{13}C NMR (75 MHz, DMSO-*d*$_6$): δ 156.41, 149.03, 139.69, 137.07, 136.27, 129.44, 128.66, 128.35, 128.19, 126.98, 126.34, 123.32, 32.68.

HRMS (ESI): *m/z* calcd. for $C_{21}H_{18}N_3O_3$: 360.13427 [M+H]$^+$; found: 360.13446.

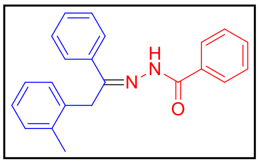

***N*'-(1-Phenyl-2-(*o*-tolyl)ethylidene)benzohydrazide (7h)**: 1-Methyl-2-(phenylethynyl)benzene (96 mg, 0.50 mmol) and benzhydrazide (68 mg, 0.50 mmol) were reacted and purified according to the general procedure for the hydrohydrazidation of alkynes to afford **7h** (80 mg, 0.24 mmol, 49% yield) as colorless solid.

^{1}H NMR (500 MHz, DMSO-*d*$_6$): δ 10.90 (s, 1H), 7.83 – 6.83 (m, 20H), 4.29 (s, 2H), 2.38 (s, 3H).

^{13}C NMR (126 MHz, DMSO-*d*$_6$): δ 163.10, 147.77, 135.94, 134.30, 133.61, 133.40, 131.72, 130.50, 130.24, 130.06, 129.48, 129.33, 129.20, 128.94, 128.82, 128.45, 128.22, 127.57, 127.53, 127.06, 126.59, 126.25, 44.40, 19.19, 18.74.

HRMS (ESI): *m/z* calcd. for $C_{22}H_{21}N_2O$: 329.1648 [M+H]$^+$; found: 329.1648.

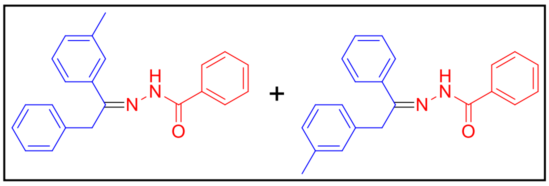

***N*'-(2-Phenyl-1-(*m*-tolyl)ethylidene)benzohydrazide and *N*'-(1-phenyl-2-(*m*-tolyl)ethylidene)benzohydrazide (7i)**: 1-Methyl-3-(phenylethynyl)benzene (96 mg, 0.50 mmol) and benzhydrazide (68 mg, 0.50 mmol) were reacted and purified according to the general procedure for the hydrohydrazidation of alkynes to afford a mixture of **7i** (107 mg, 0.33 mmol, 68% yield, 70:30 ratio) as off-white solid.

^{1}H NMR (500 MHz, DMSO-*d*$_6$): δ 10.95 (s, 2H), 7.96 – 6.94 (m, 26H), 4.41 (s, 1H), 4.38 (s, 2H), 2.31 (s, 2H), 2.24 (s, 3H).

^{13}C NMR (126 MHz, DMSO-*d*$_6$): δ 164.12, 154.92, 137.76, 137.52, 137.43, 136.41, 136.29, 134.01, 131.46, 129.99, 129.27, 128.81, 128.67, 128.61, 128.36, 128.24, 128.16, 127.86, 127.26, 127.04, 126.81, 126.33, 125.18, 124.16, 32.44, 21.06, 20.99.

HRMS (ESI): *m/z* calcd. for $C_{22}H_{21}N_2O$: 329.1648 [M+H]$^+$; found: 329.1649.

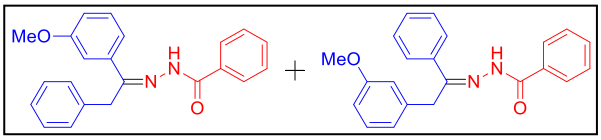

N'-(1-(3-Methoxyphenyl)-2-phenylethylidene)benzohydrazide and N'-(2-(3-methoxyphenyl)-1-phenylethylidene)benzohydrazide (7j): 1-Methoxy-3-(phenylethynyl)benzene (104 mg, 0.50 mmol) and benzhydrazide (68 mg, 0.50 mmol) were reacted and purified according to the general procedure for the hydrohydrazidation of alkynes to afford a mixture of **7j** (125 mg, 0.36 mmol, 73% yield, 60:40 ratio) as pale yellow, viscous oil.

^{1}H NMR (500 MHz, DMSO-d_6): δ 11.01 (s, 1H), 7.93 – 6.73 (m, 14H), 4.41 (s, 2H), 3.85 – 3.54 (m, 3H).

^{13}C NMR (126 MHz, DMSO-d_6): δ 164.26, 159.52, 159.22, 155.07, 138.93, 137.89, 137.51, 136.50, 134.00, 131.56, 129.82, 129.46, 129.39, 128.74, 128.42, 128.27, 128.21, 127.92, 126.88, 126.40, 120.35, 119.42, 114.95, 114.31, 112.41, 111.58, 55.07, 54.95, 32.57.

HRMS (EI): *m/z* calcd. for $C_{22}H_{20}N_2O_2$: 344.1519 [M]; found: 344.1519.

2. NMR spectra

2.1. NMR spectra of complexes

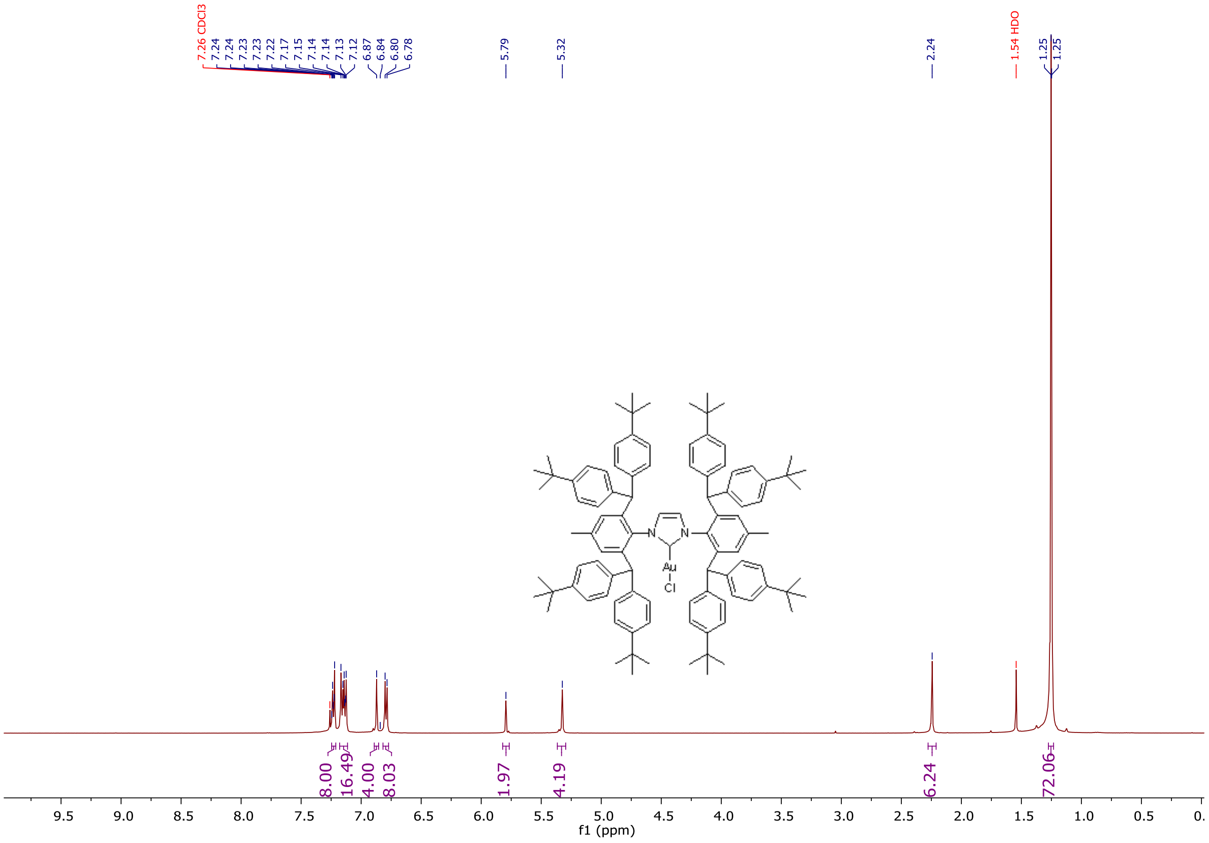

Figure S1: ^{1}H NMR of complex 2a in $CDCl_3$.

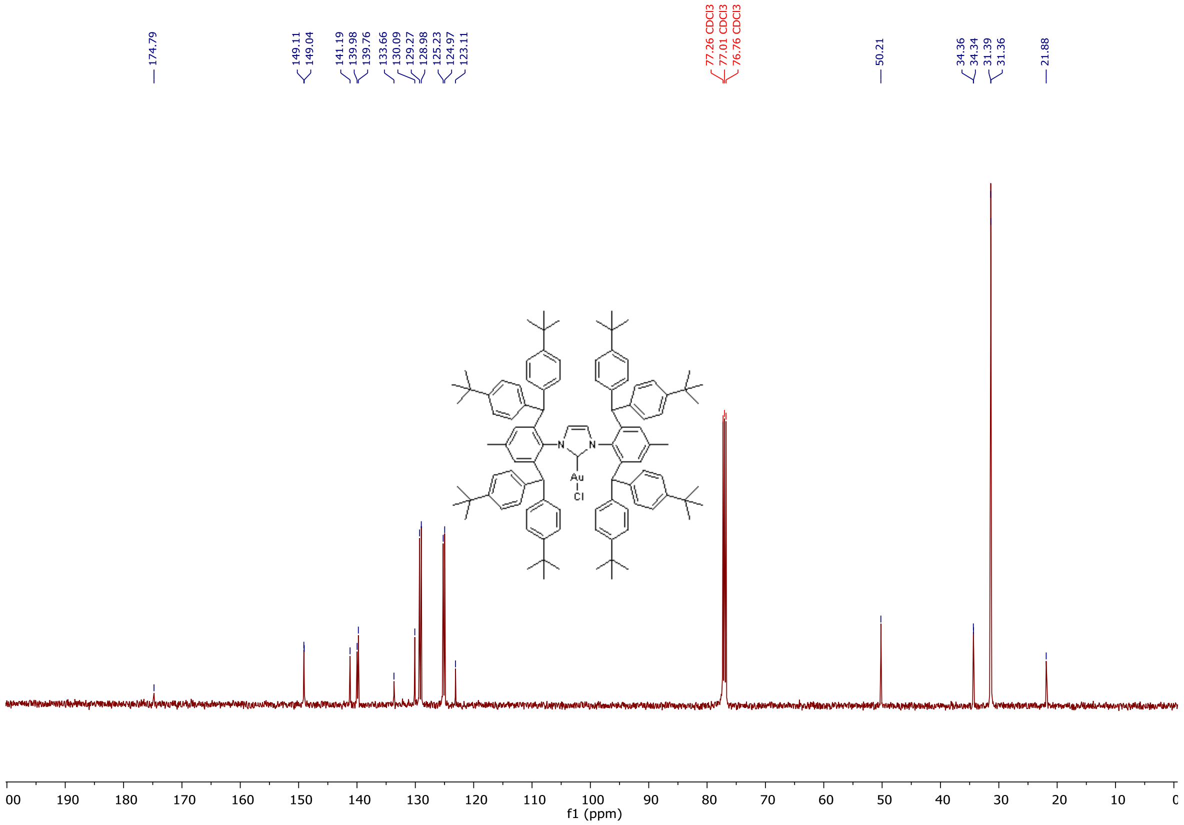

Figure S2: ^{13}C NMR of complex 2a in $CDCl_3$.

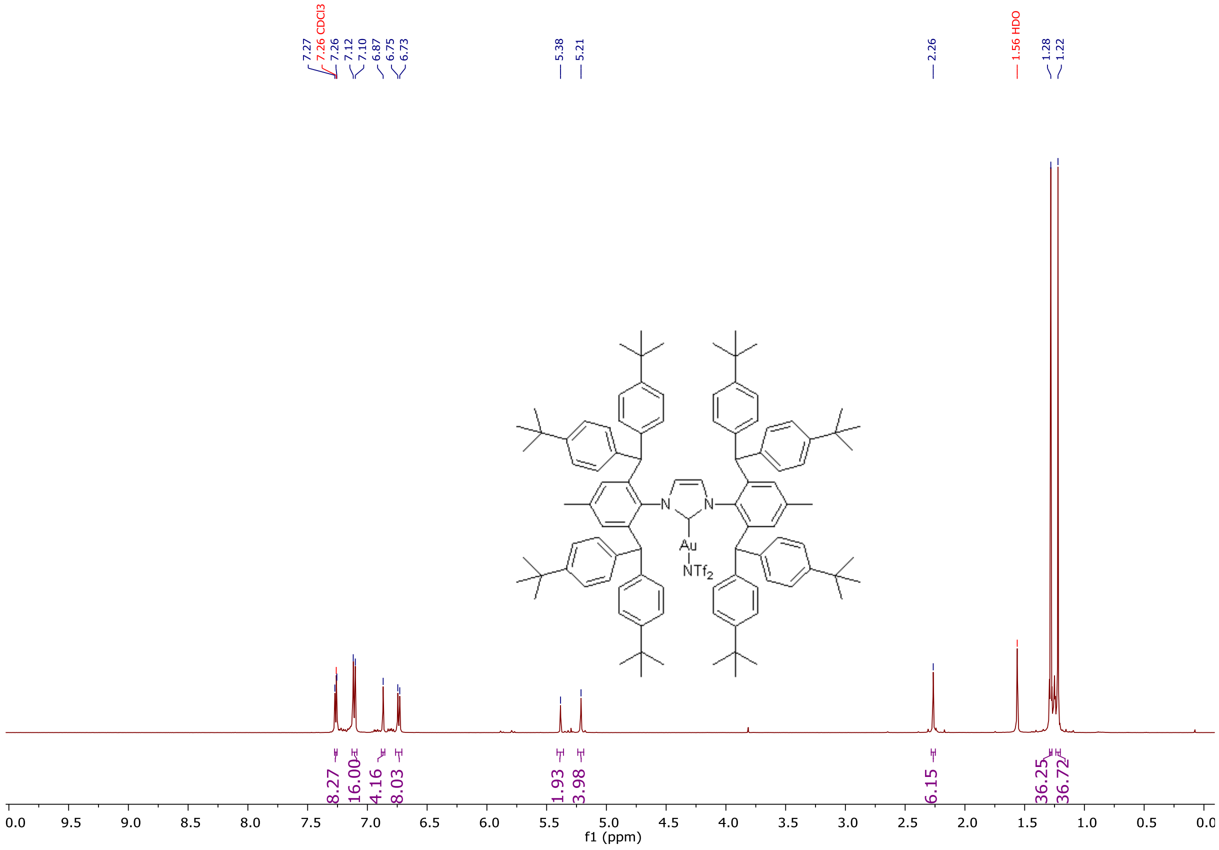

Figure S3: ^{1}H NMR of complex 2 in $CDCl_3$.

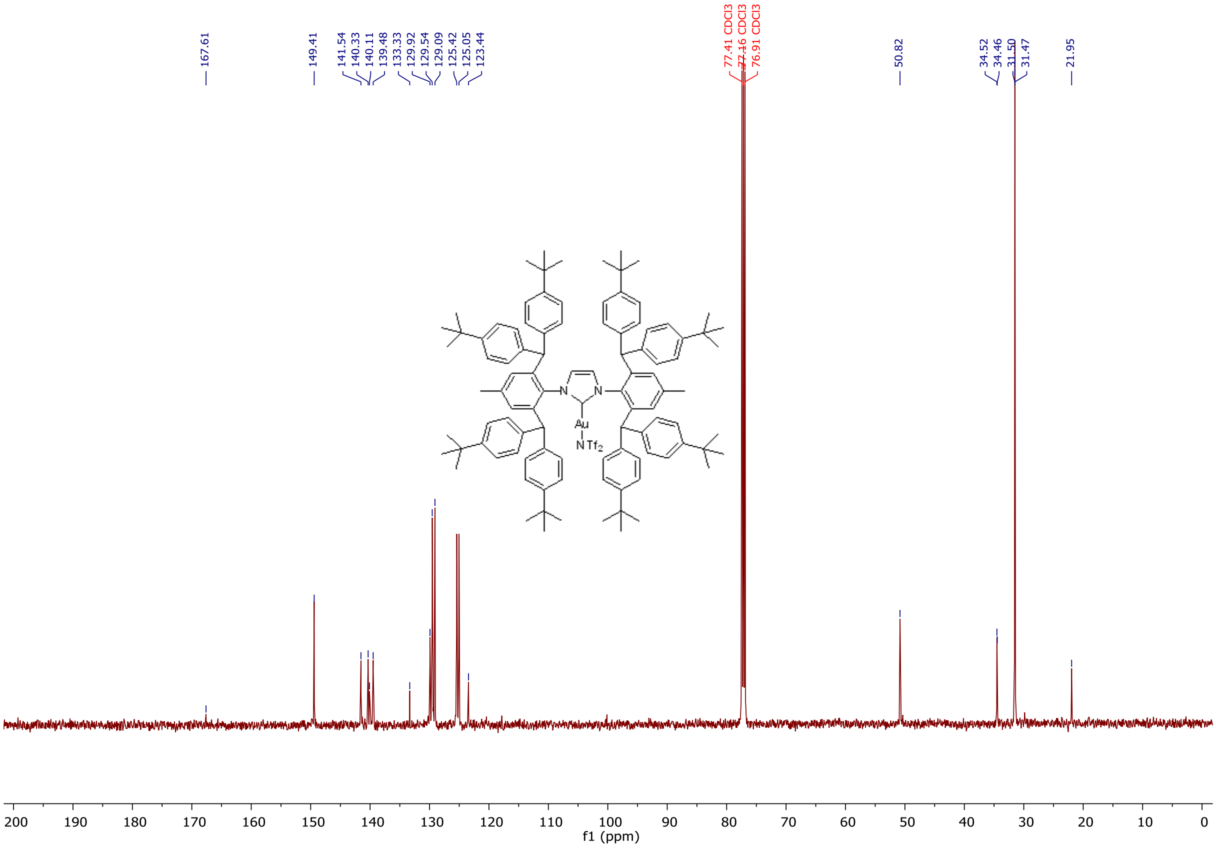

Figure S4: ^{13}C NMR of complex 2 in $CDCl_3$.

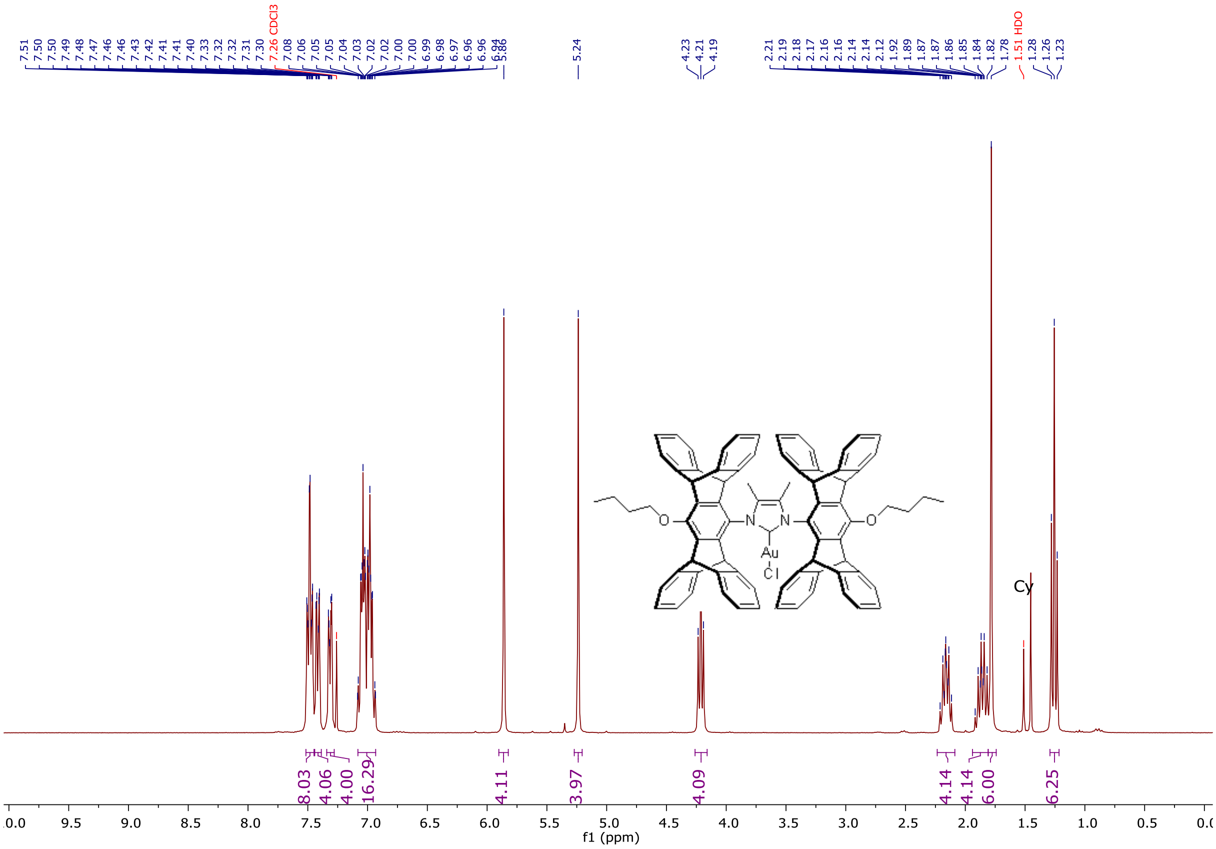

Figure S5: ^{1}H NMR of complex 1a in in $CDCl_3$.

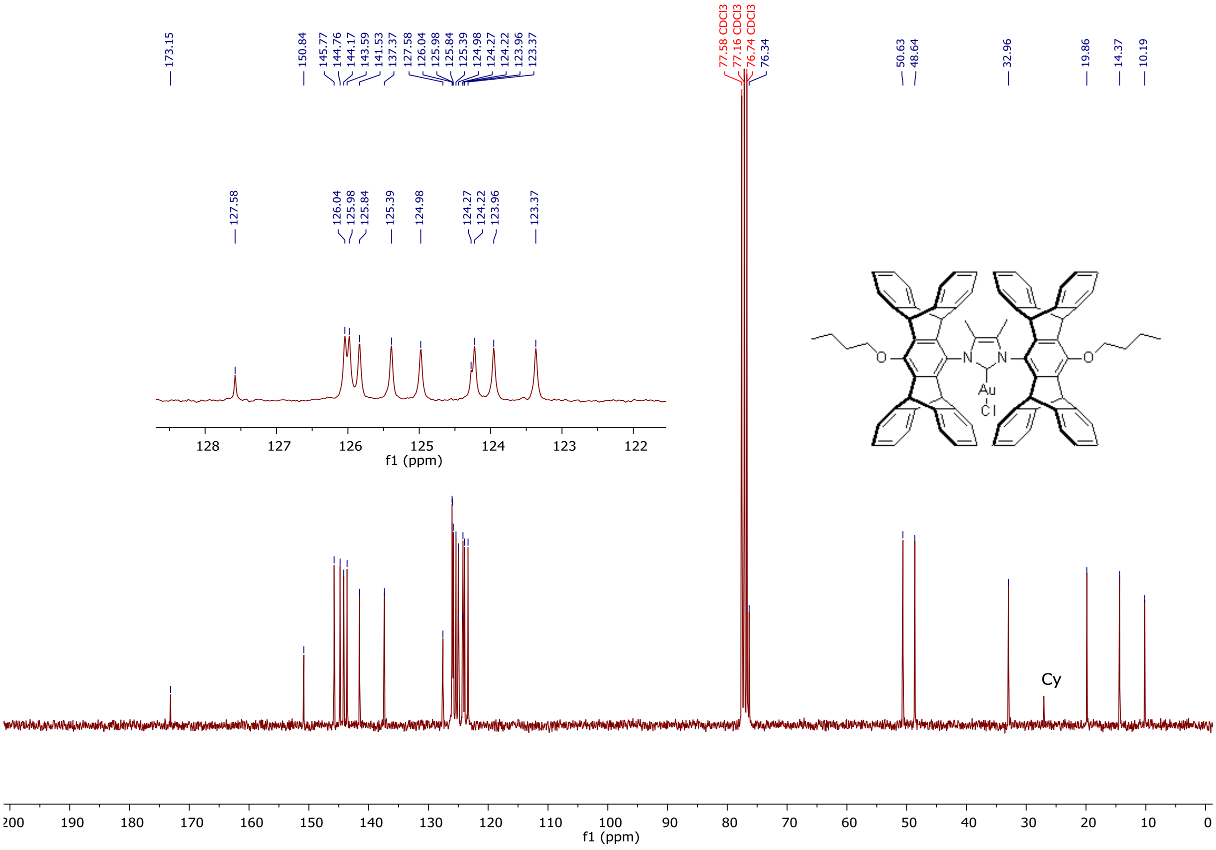

Figure S6: ^{13}C NMR of complex 1a in in $CDCl_3$.

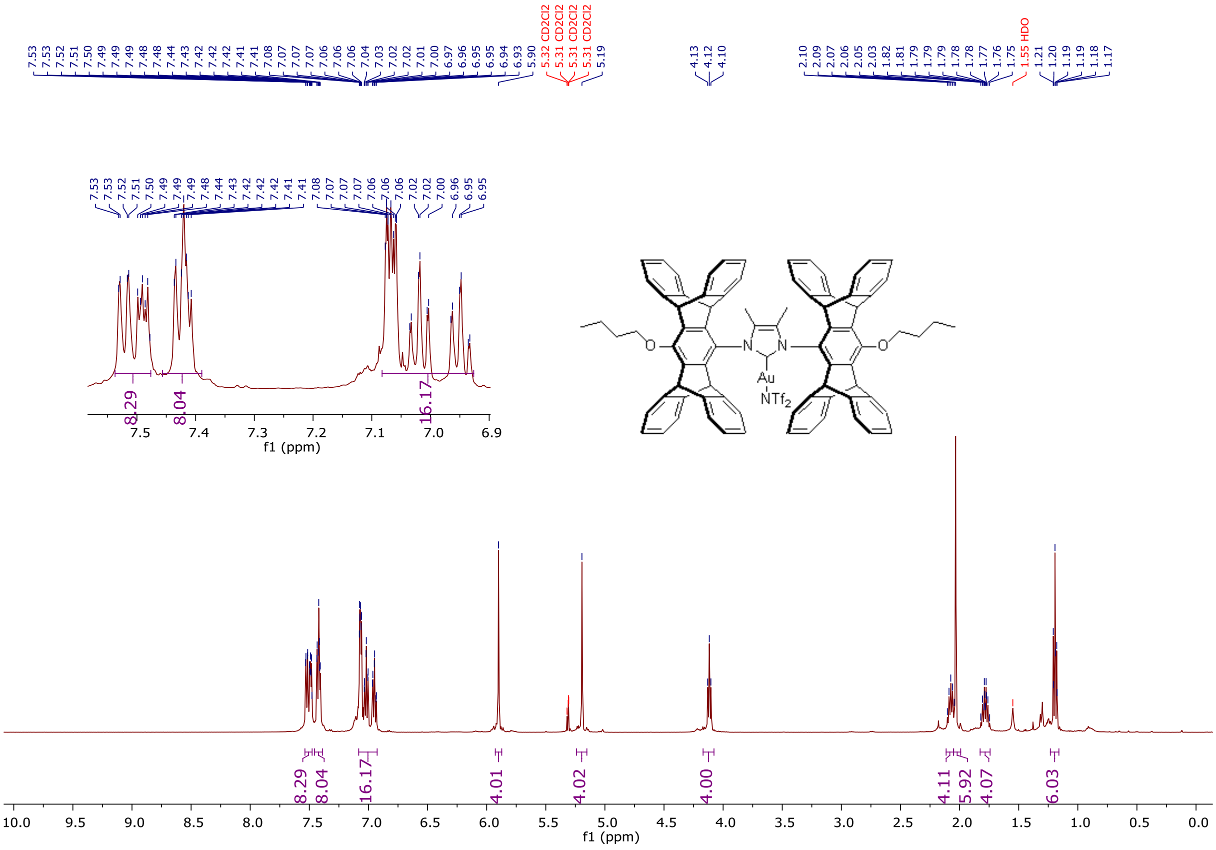

Figure S7: ^{1}H NMR of complex 1 in CD_2Cl_2.

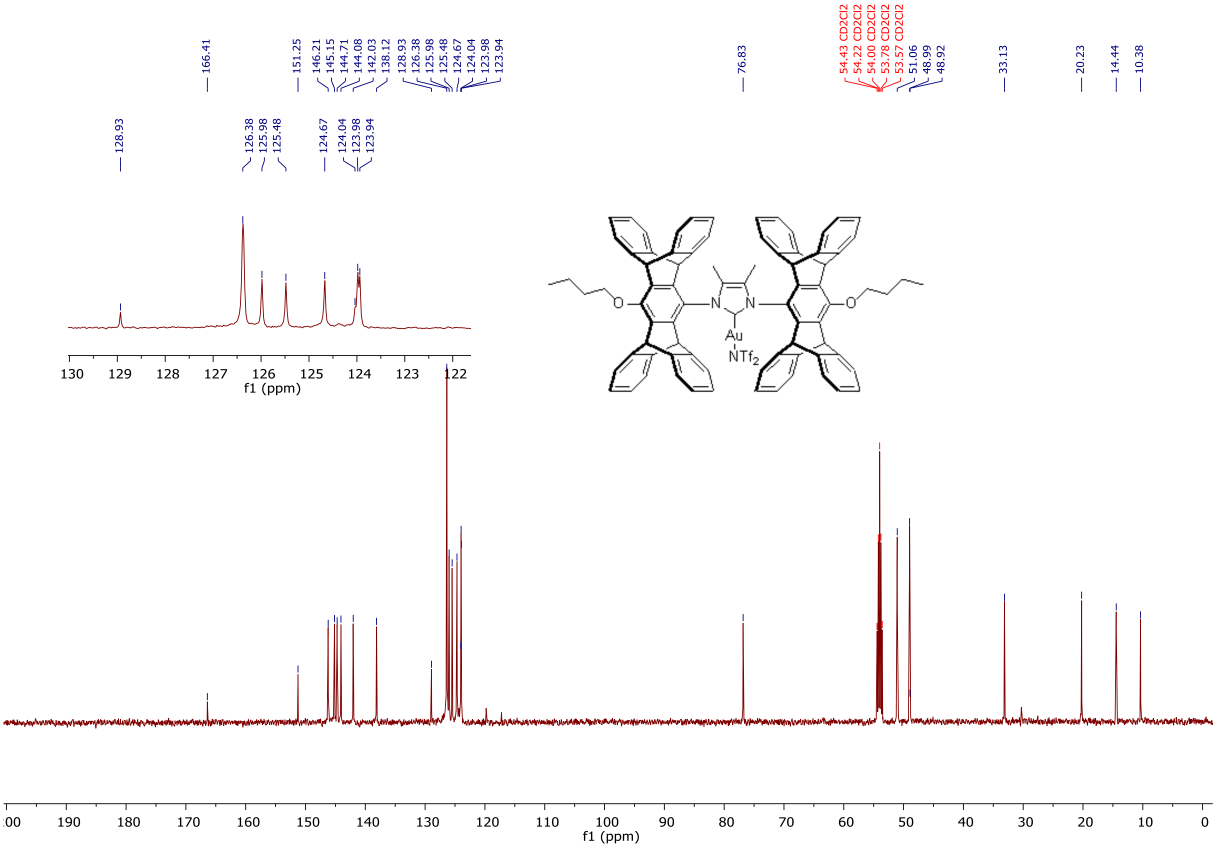

Figure S8: ^{13}C NMR of complex 1 in CD_2Cl_2.

2.2. NMR spectra of hydrohydrazidation products

2.2.1. Terminal alkynes as substrates

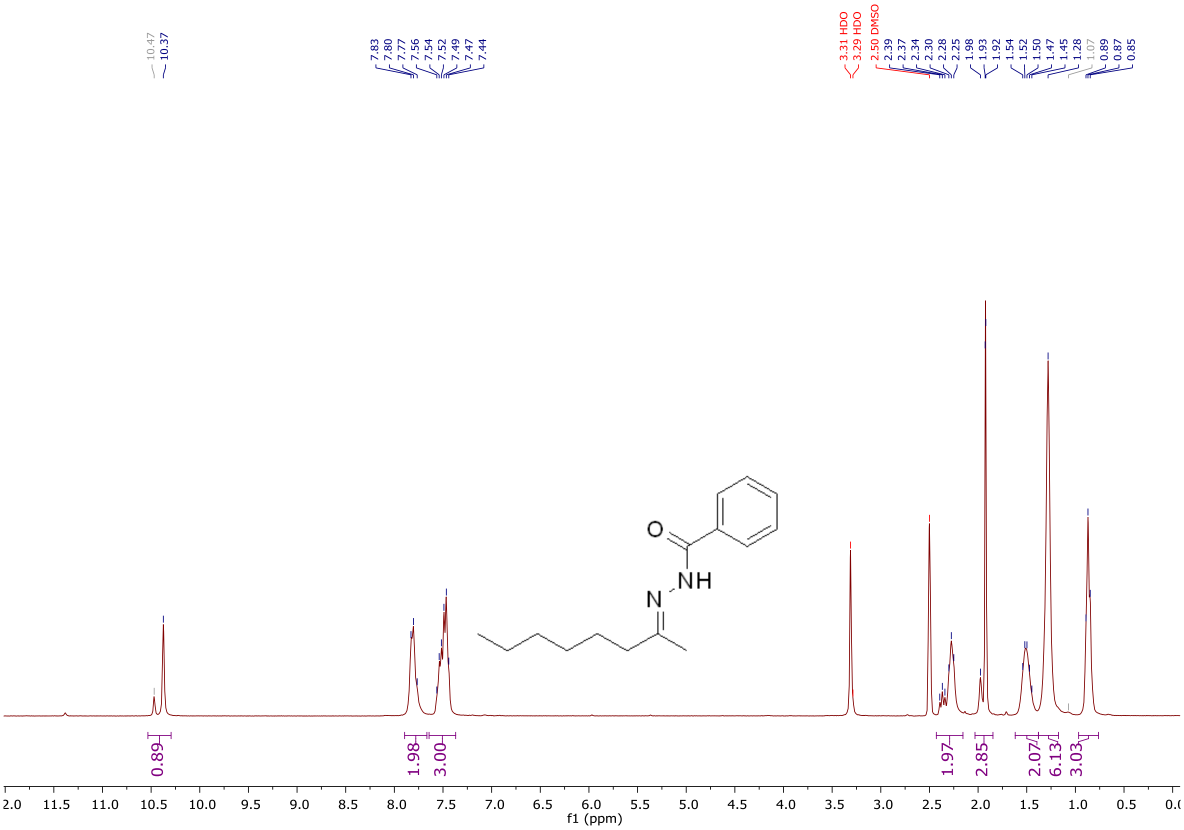

Figure S9: ^{1}H NMR of *N*'-(octan-2-ylidene)benzohydrazide (6a) in DMSO-d_6.

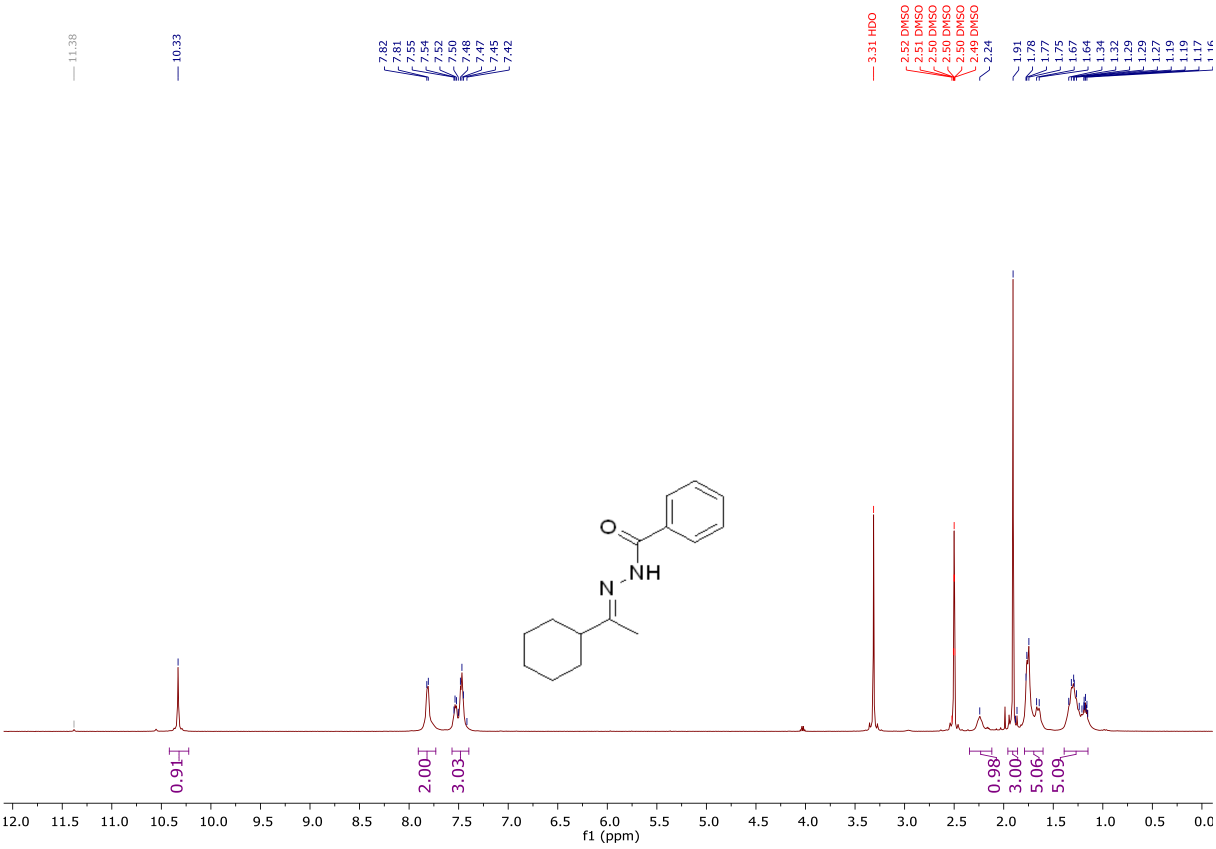

Figure S10: ^{1}H NMR of *N*'-(1-cyclohexylethylidene)benzohydrazide (6b) in DMSO-d_6.

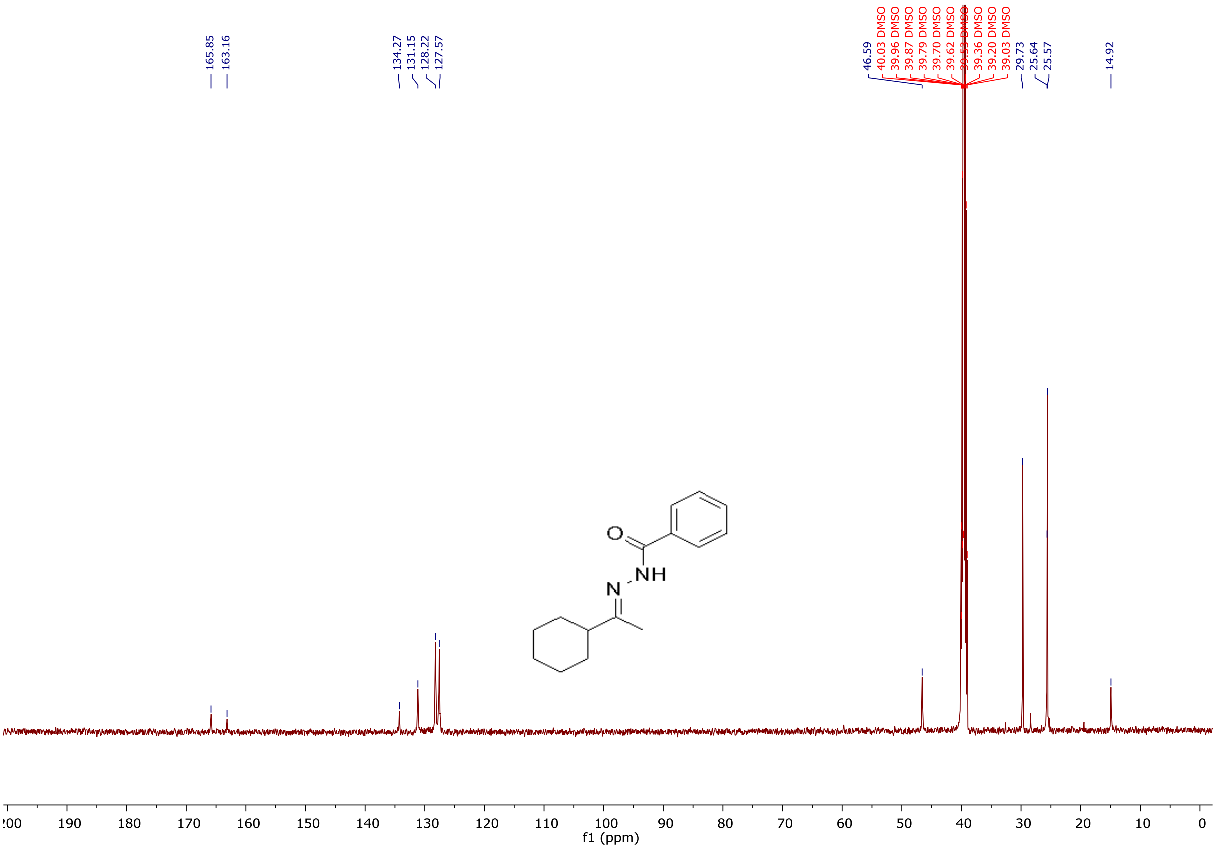

Figure S11: ^{13}C NMR of *N*'-(1-cyclohexylethylidene)benzohydrazide (6b) in DMSO-d_6.

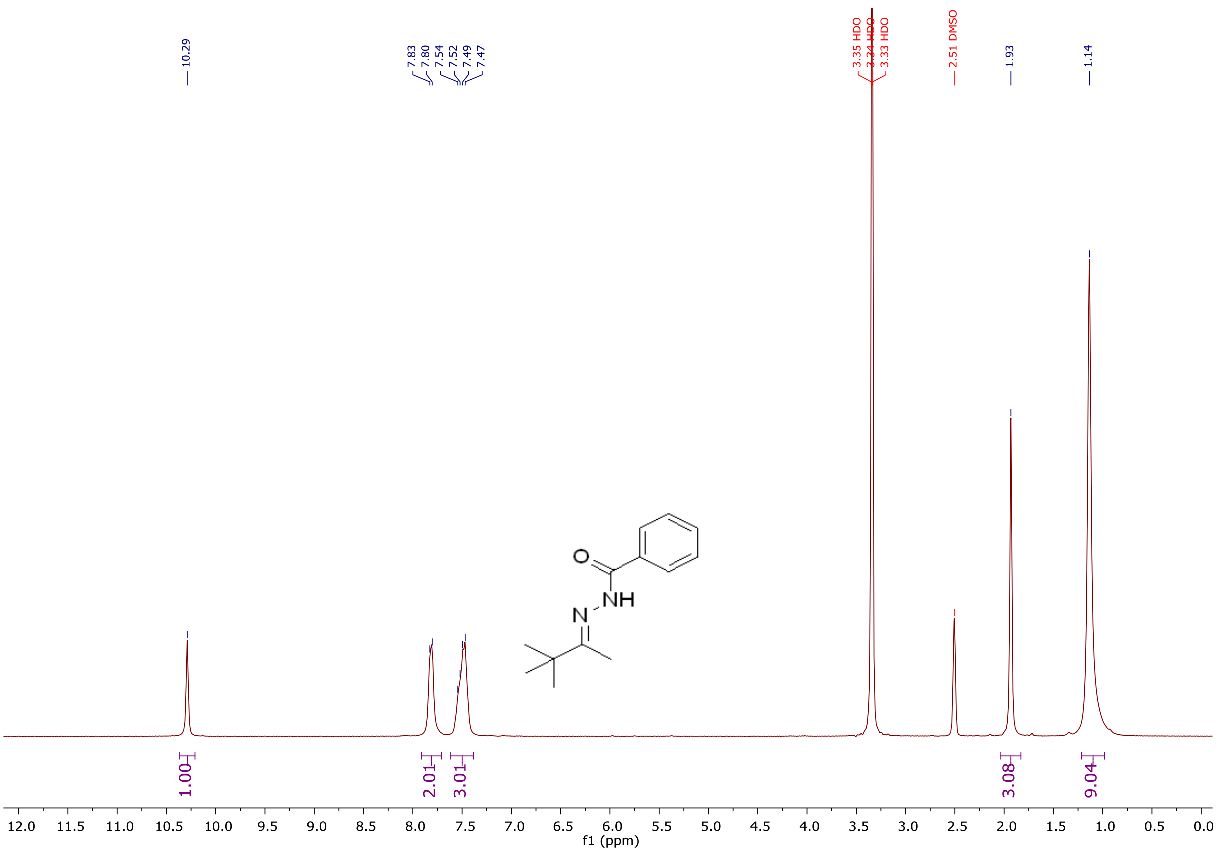

Figure S12: ^{1}H NMR of *N*'-(3,3-dimethylbutan-2-ylidene)benzohydrazide (6c) in DMSO-d_6.

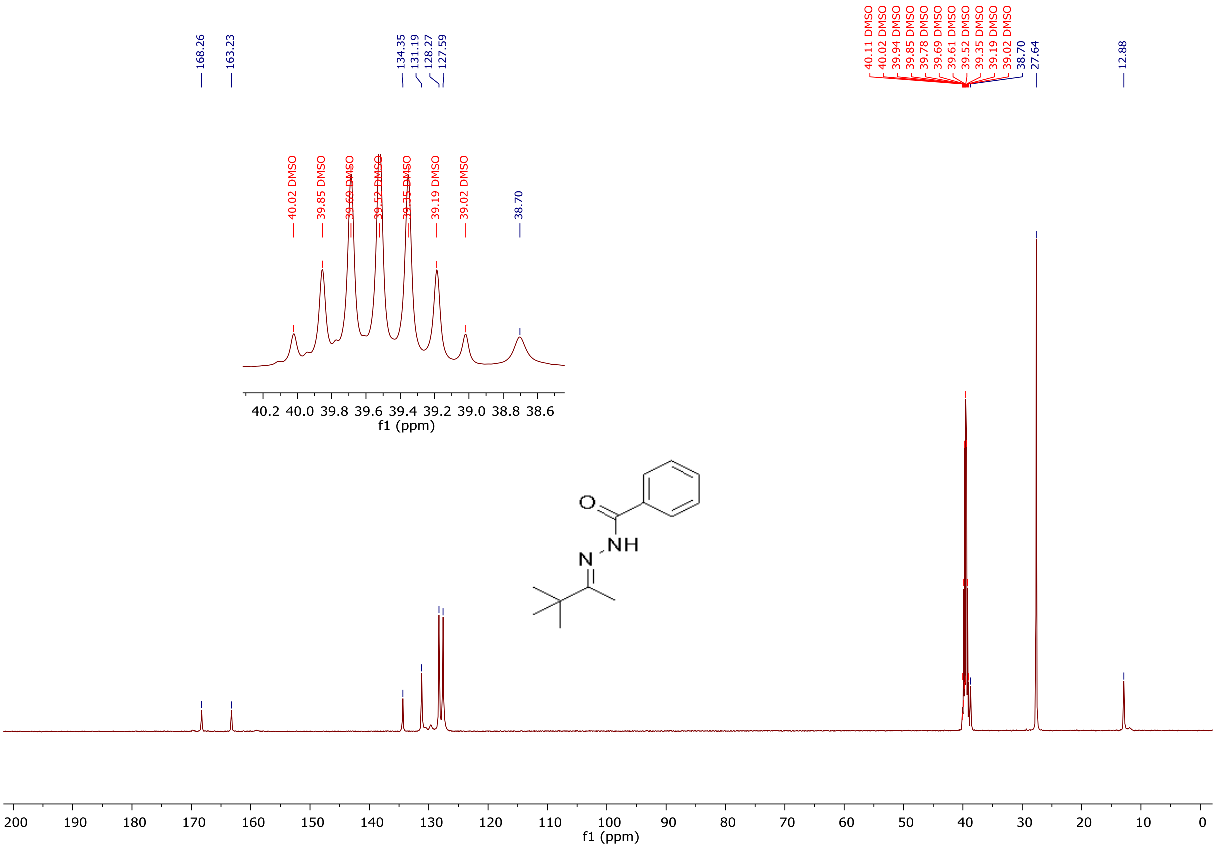

Figure S13: ^{13}C NMR of *N*'-(3,3-dimethylbutan-2-ylidene)benzohydrazide (6c) in DMSO-d_6.

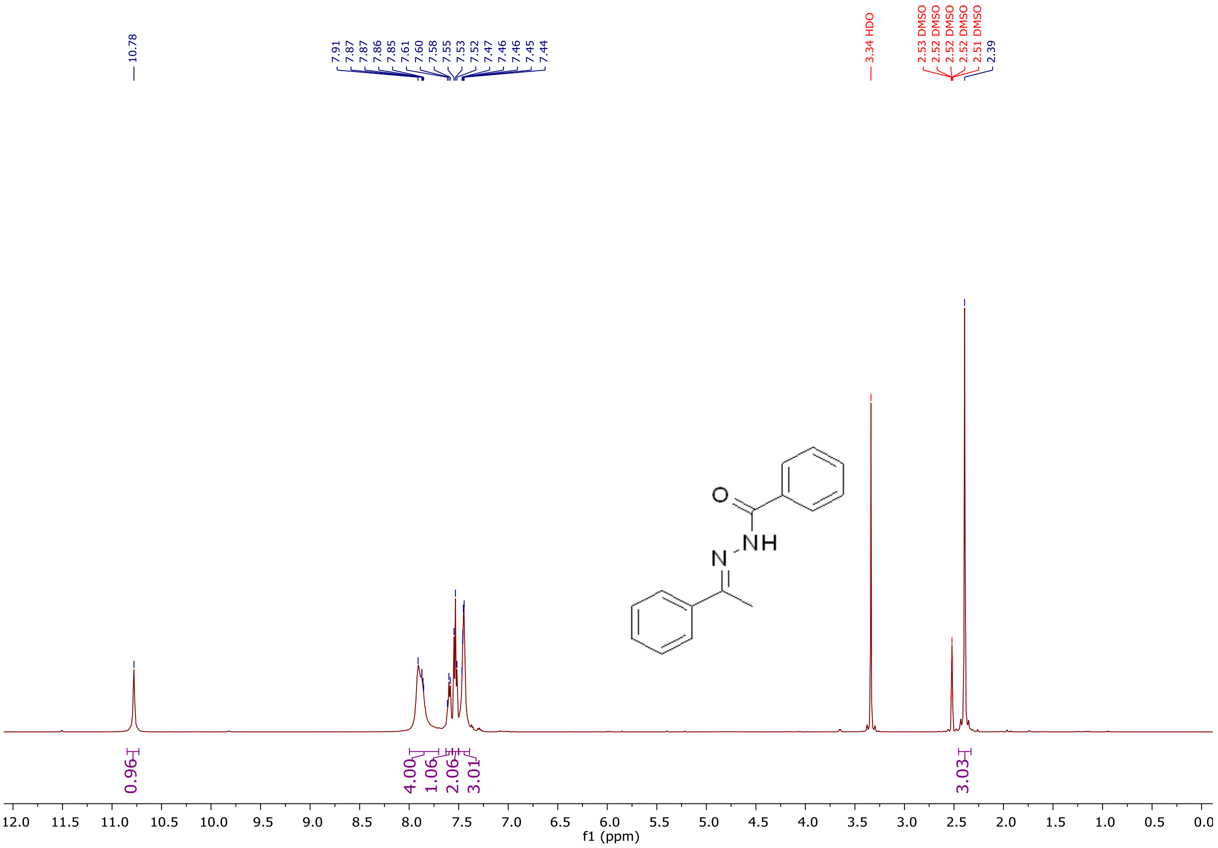

Figure S14: ^{1}H NMR of *N*'-(1-phenylethylidene)benzohydrazide (6d) in DMSO-d_6.

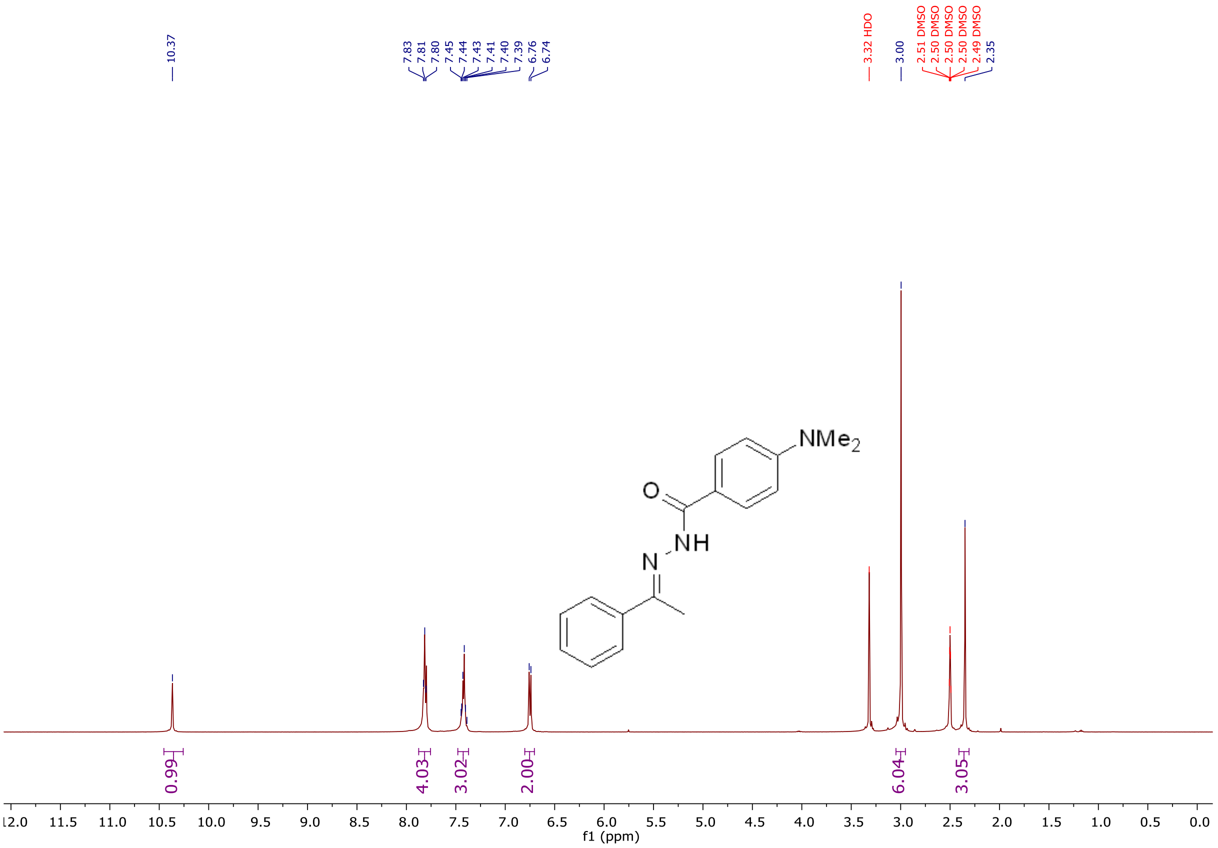

Figure S15: ^{1}H NMR of 4-(dimethylamino)-*N*'-(1-phenylethylidene)benzohydrazide (6e) in DMSO-d_6.

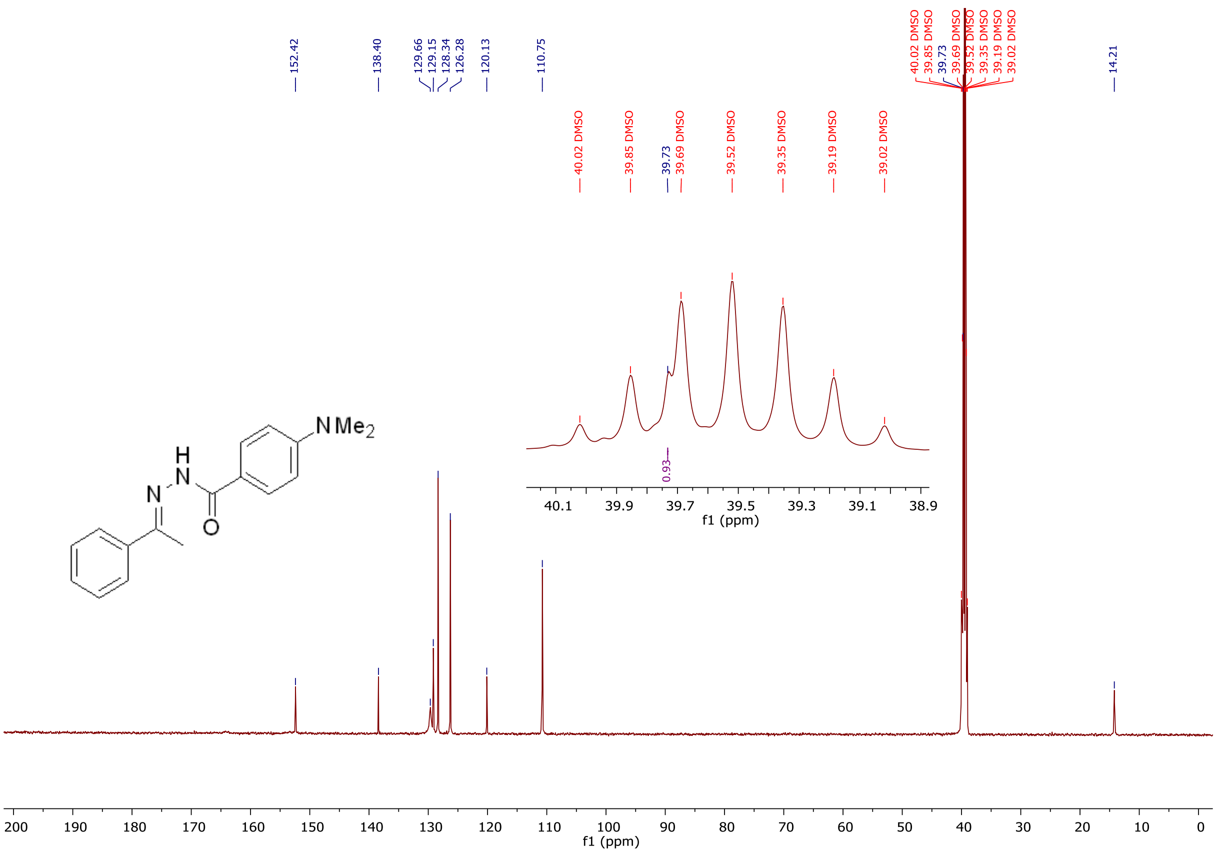

Figure S16: ^{13}C NMR of 4-(dimethylamino)-*N*'-(1-phenylethylidene)benzohydrazide (6e) in DMSO-d_6.

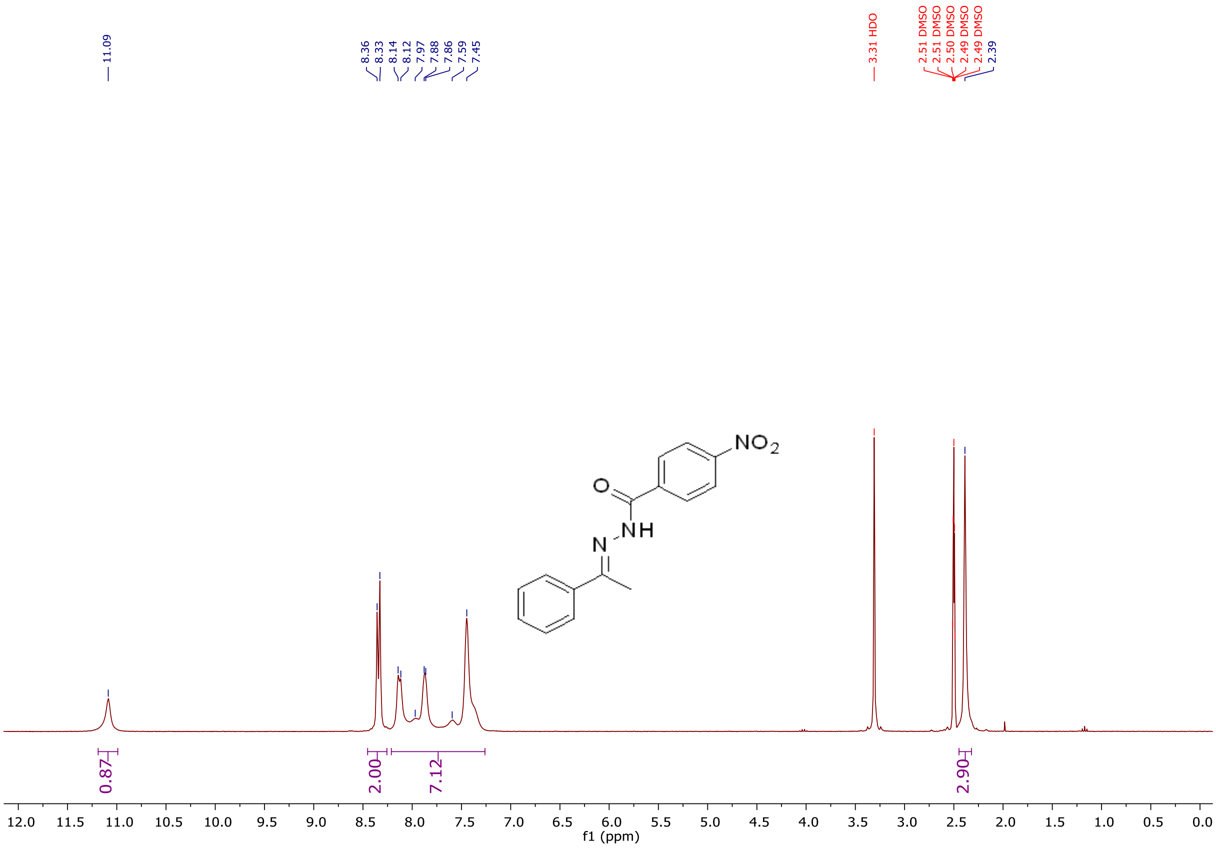

Figure S17: ^{1}H NMR of 4-nitro-*N*'-(1-phenylethylidene)benzohydrazide (6f) in DMSO-d_6.

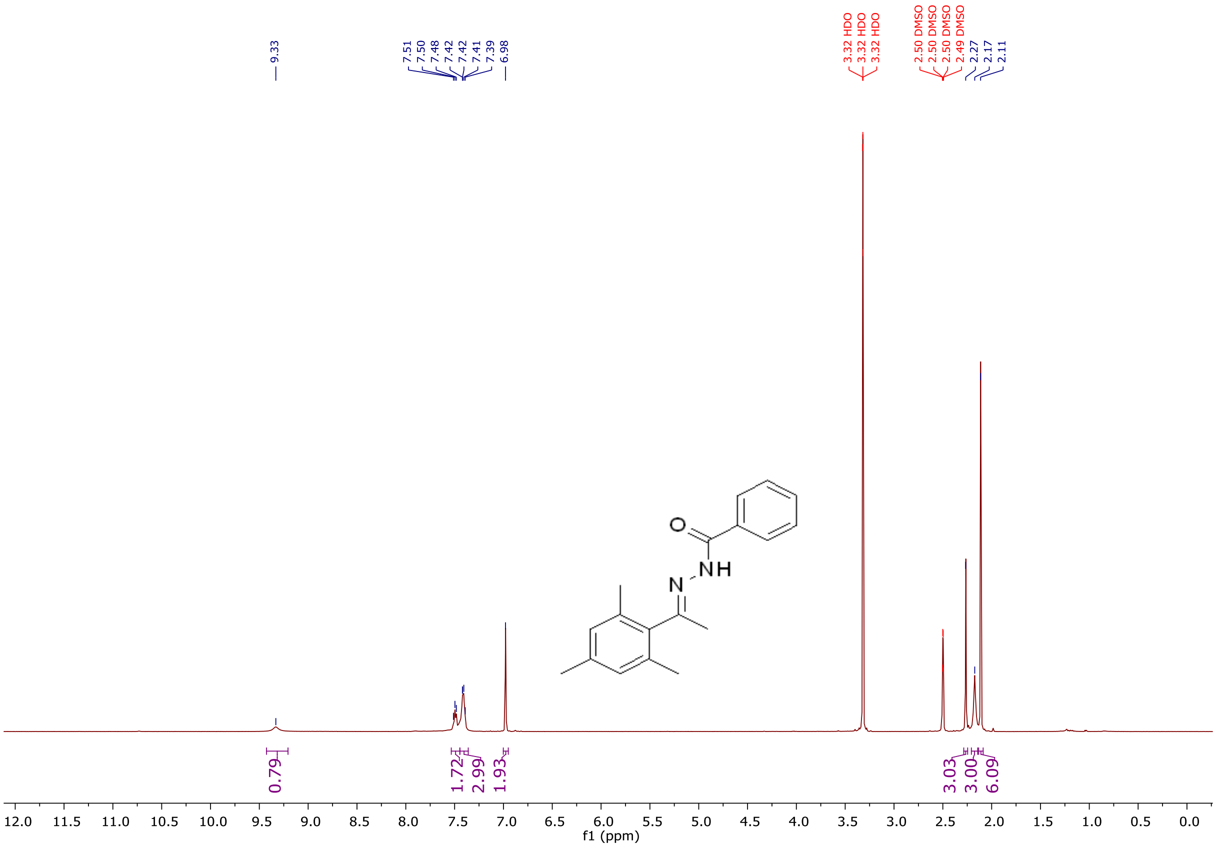

Figure S18: ^{1}H NMR of *N*'-(1-mesitylethylidene)benzohydrazide (6g) in DMSO-d_6.

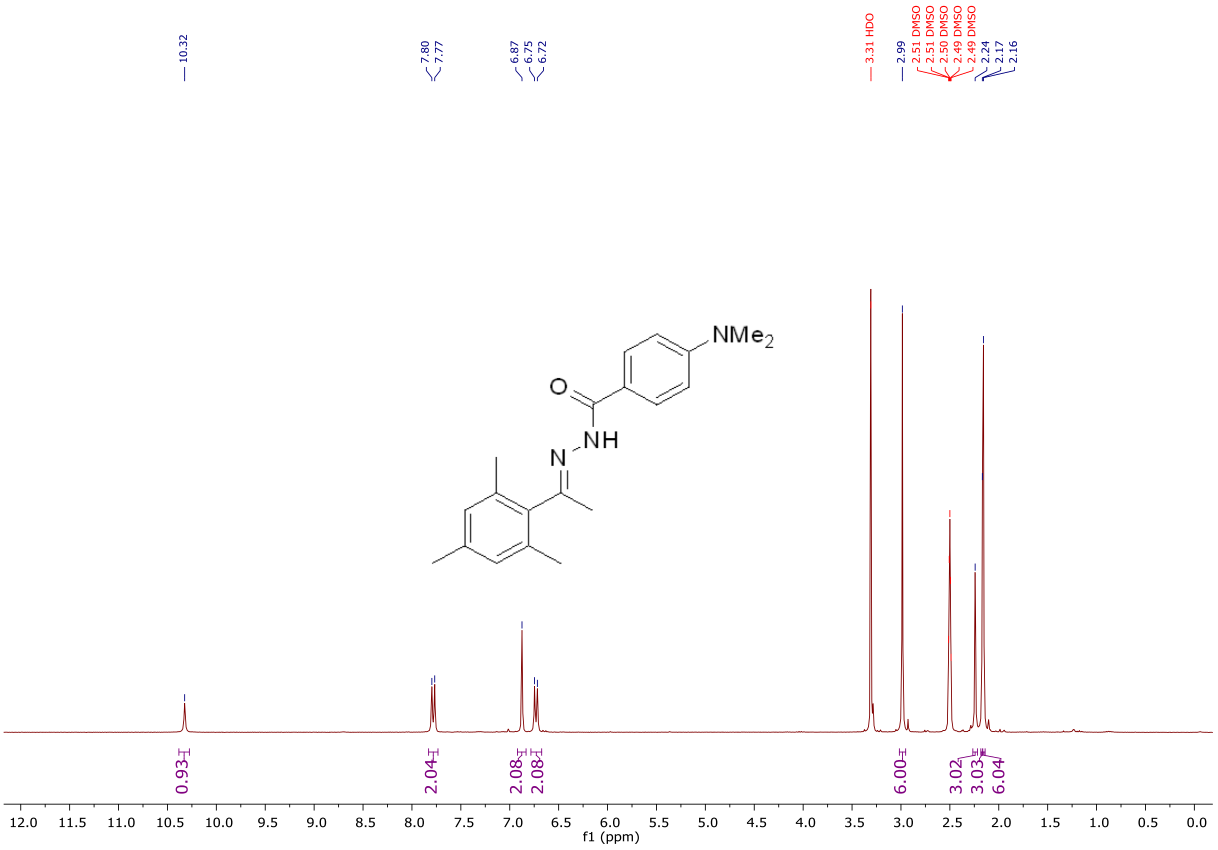

Figure S19: ^{1}H NMR of 4-(dimethylamino)-*N*'-(1-mesitylethylidene)benzohydrazide (6h) in DMSO-d_6.

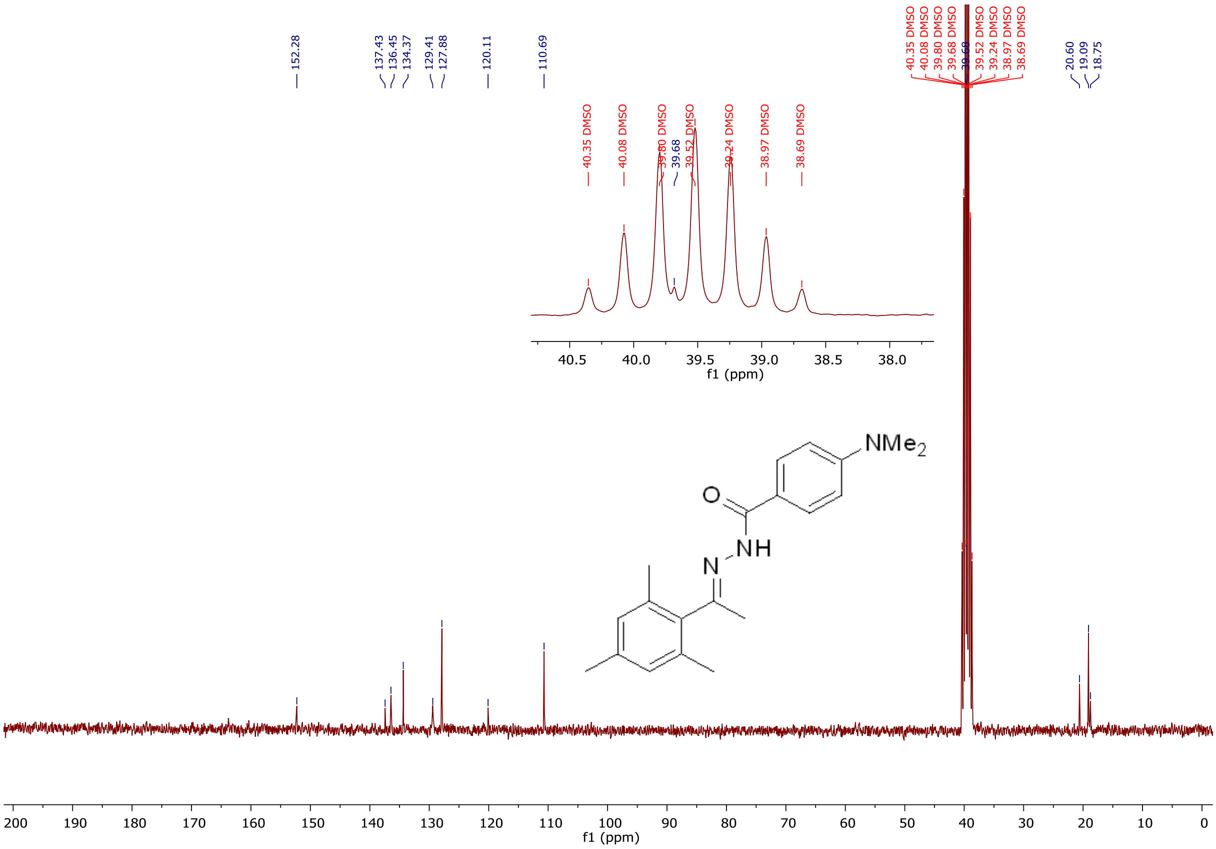

Figure S20: ^{13}C NMR of 4-(dimethylamino)-*N*'-(1-mesitylethylidene)benzohydrazide (6h) in DMSO-d_6.

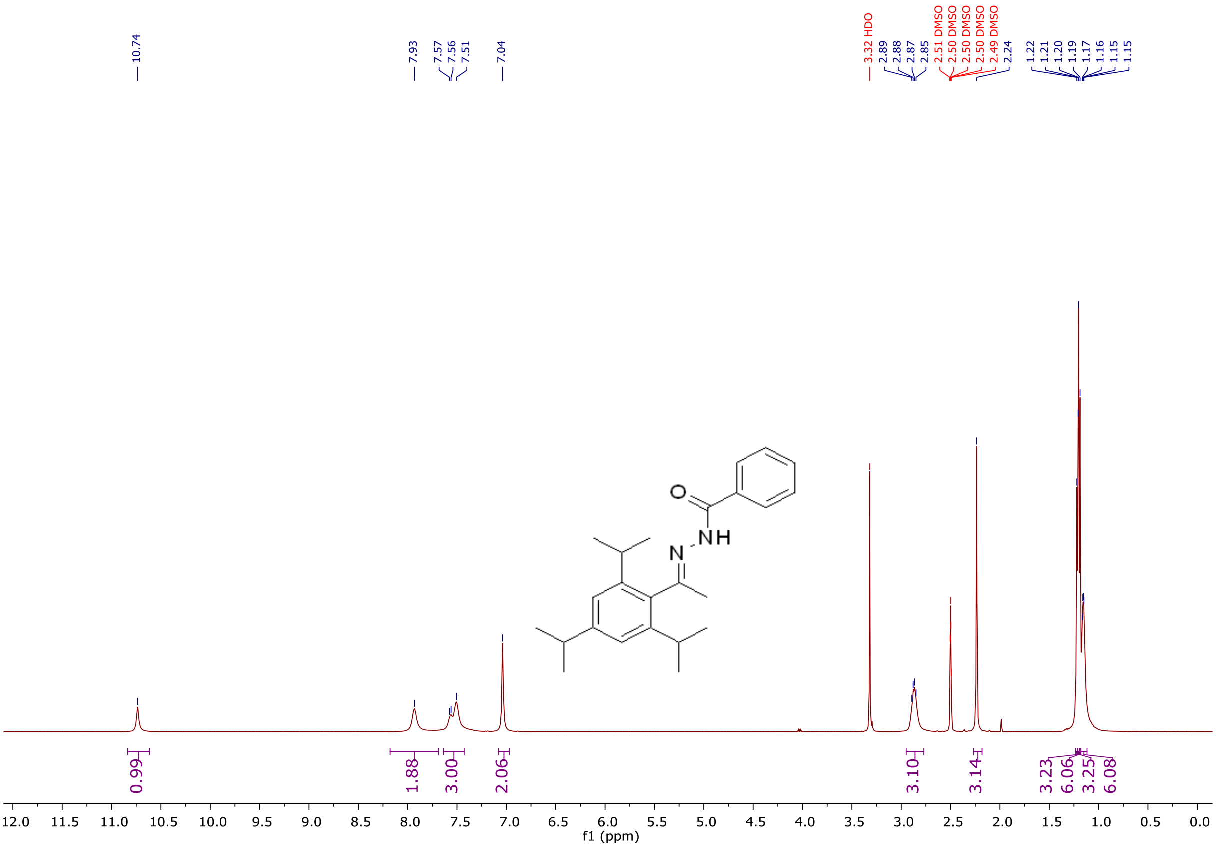

Figure S21: ^{1}H NMR of *N*'-(1-(2,4,6-triisopropylphenyl)ethylidene)benzohydrazide (6i) in DMSO-d_6.

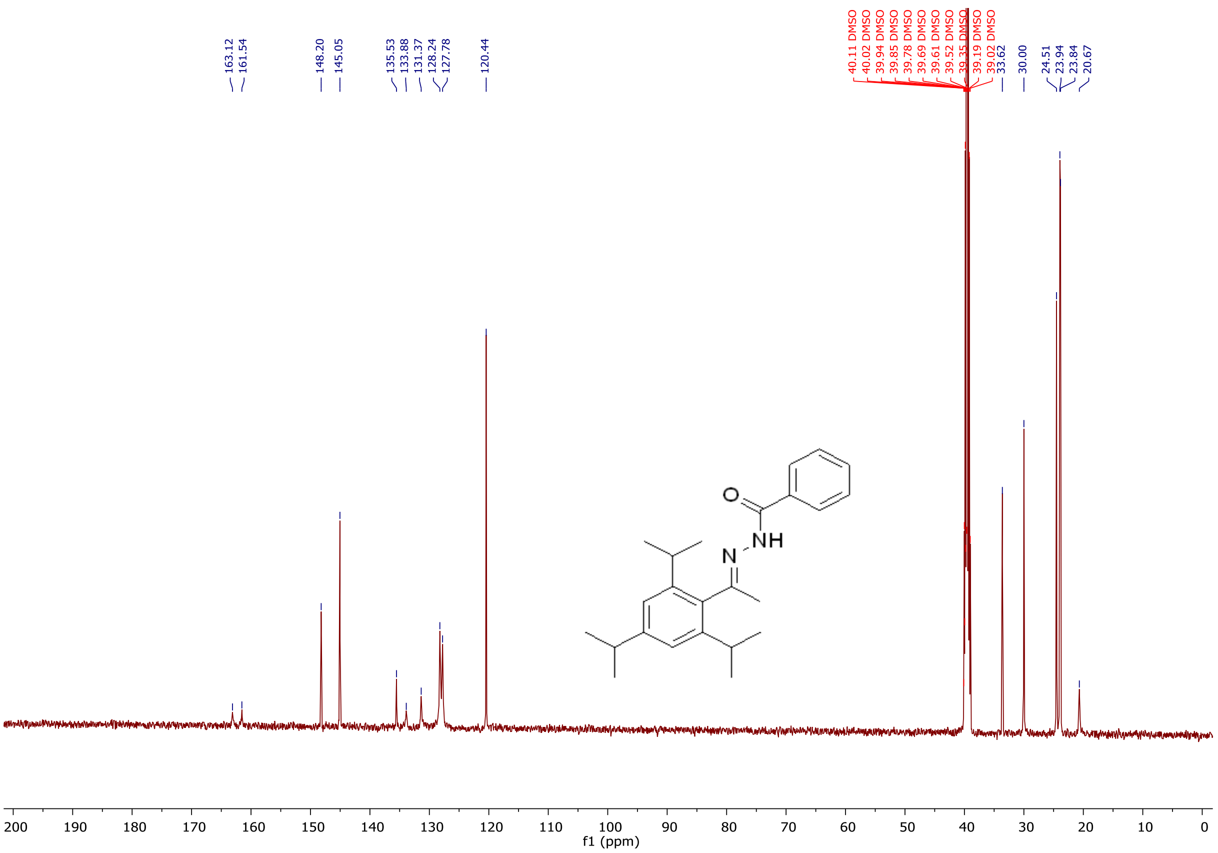

Figure S22: ^{13}C NMR of *N'*-(1-(2,4,6-triisopropylphenyl)ethylidene)benzohydrazide (6i) in DMSO-d_6.

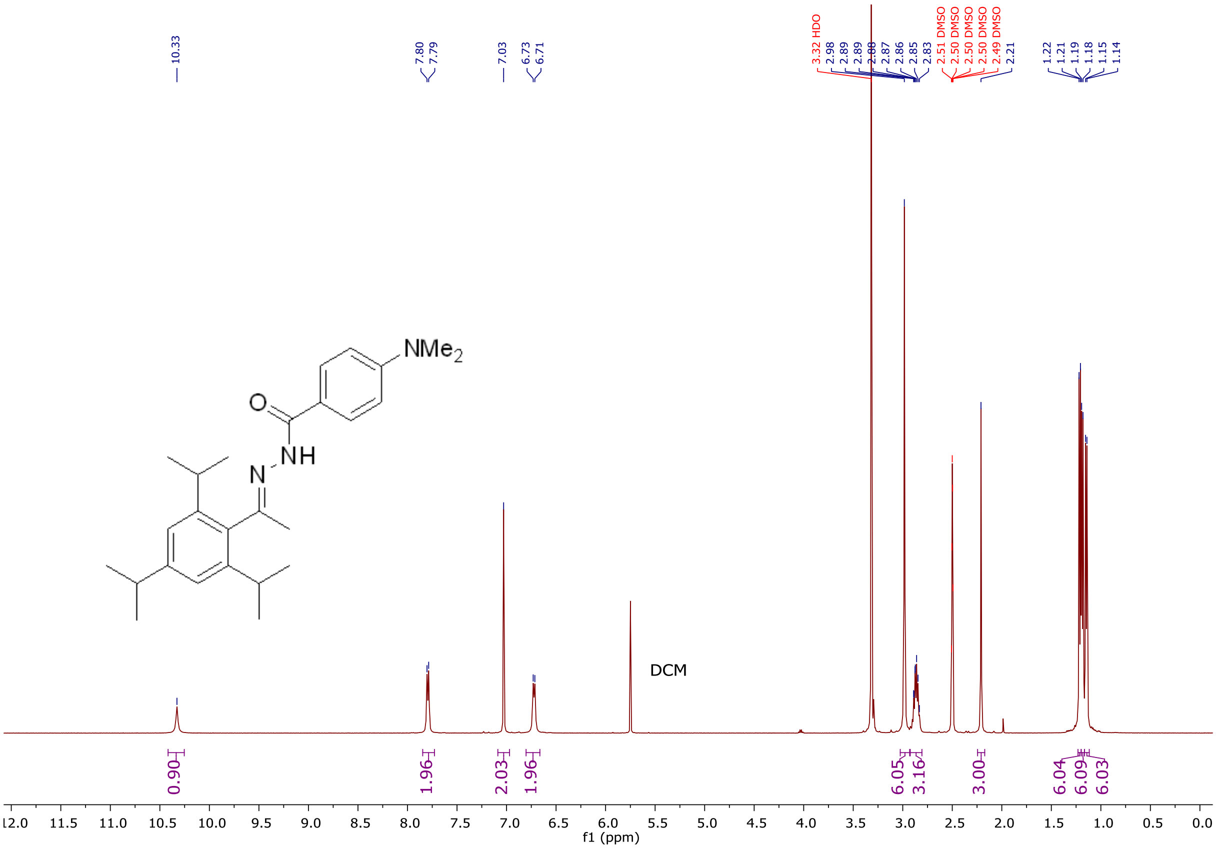

Figure S23: ^{1}H NMR of 4-(dimethylamino)-*N'*-(1-(2,4,6-triisopropylphenyl)ethylidene)benzohydrazide (6j) in DMSO-d_6.

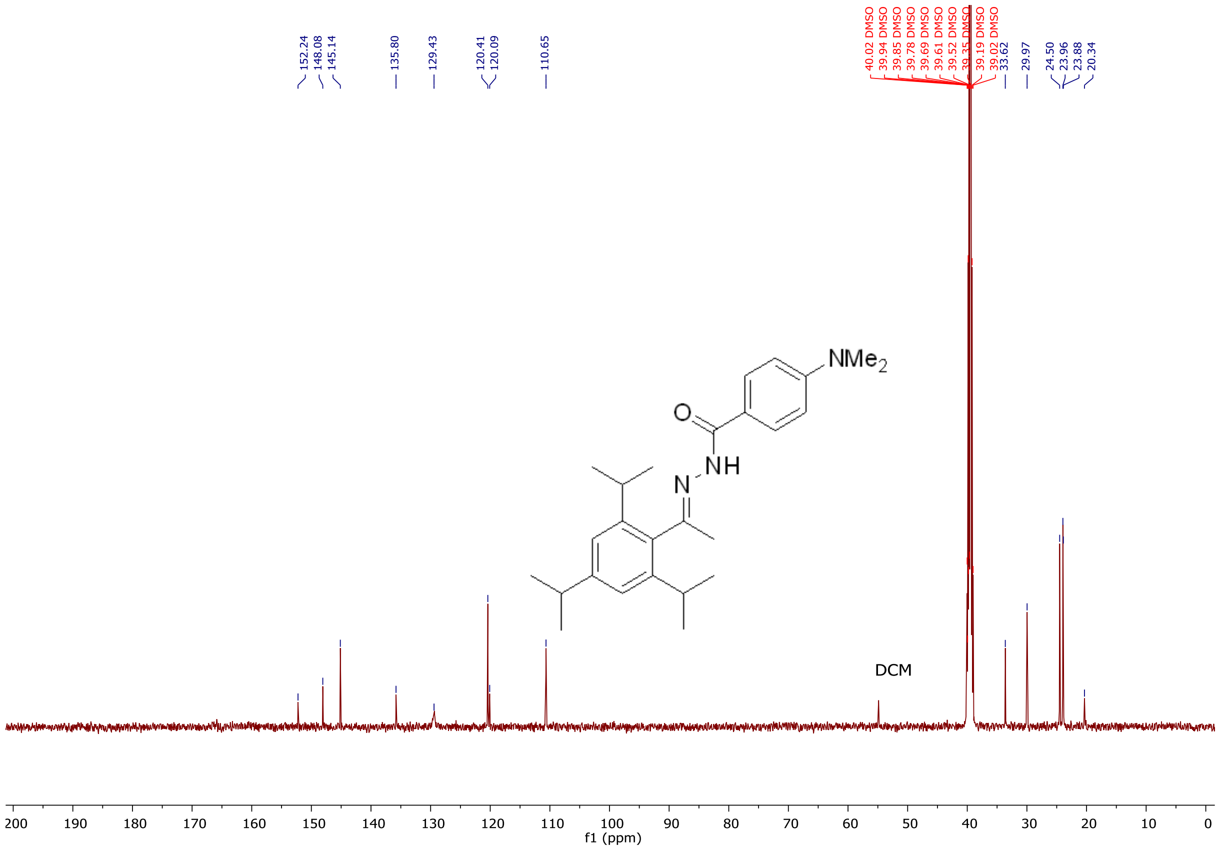

Figure S24: ^{13}C NMR of 4-(dimethylamino)-*N*'-(1-(2,4,6-triisopropylphenyl)ethylidene)benzohydrazide (6j) in DMSO-d_6.

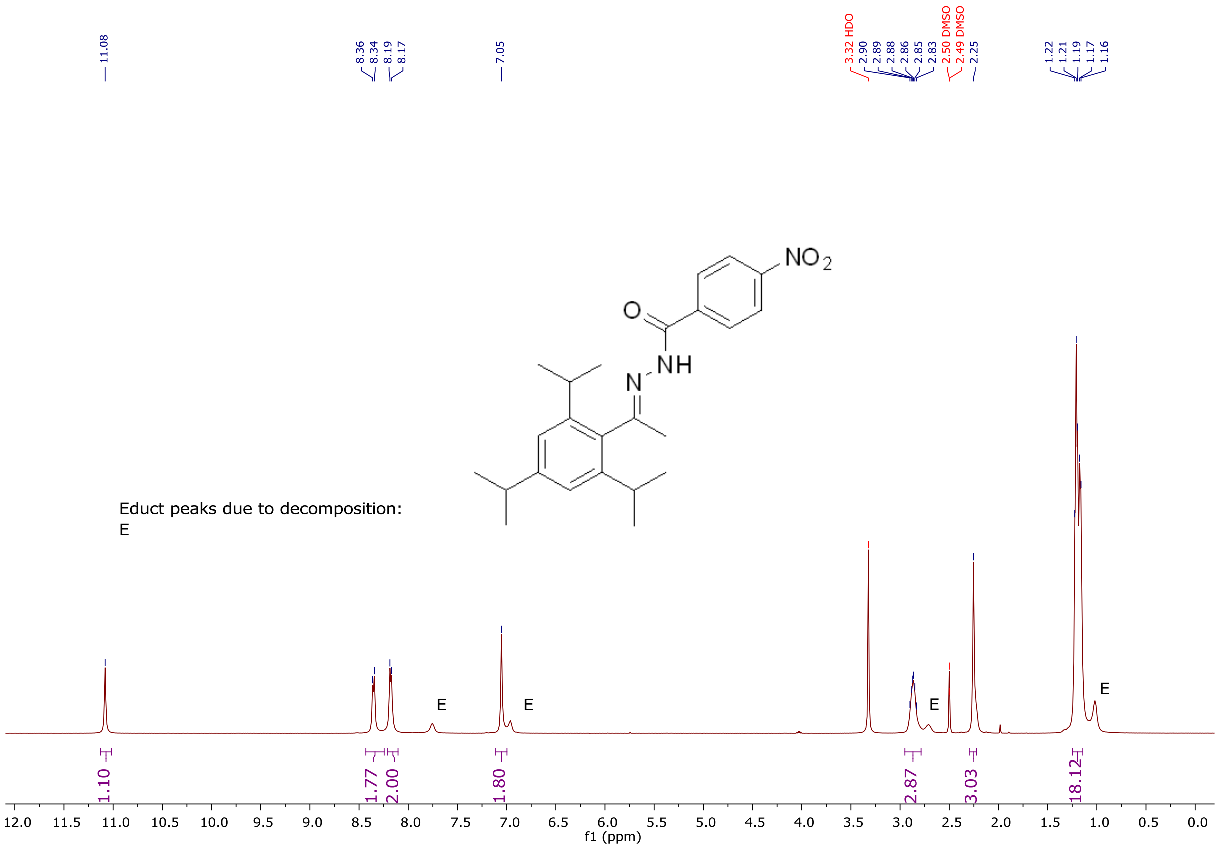

Figure S25: ^{1}H NMR of 4-nitro-*N*'-(1-(2,4,6-triisopropylphenyl)ethylidene)benzohydrazide (6k) in DMSO-d_6.

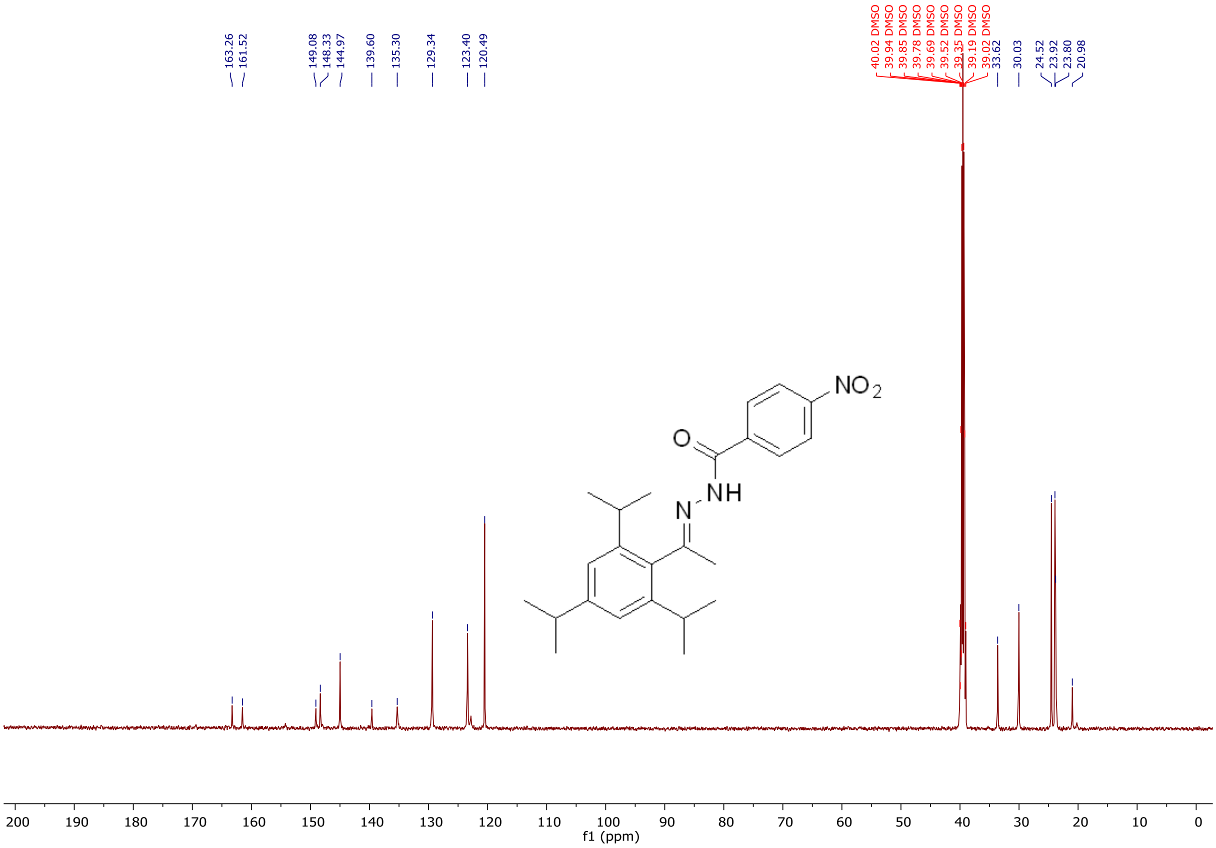

Figure S26: ^{13}C NMR of 4-nitro-*N*'-(1-(2,4,6-triisopropylphenyl)ethylidene)benzohydrazide in (6k) DMSO-d_6.

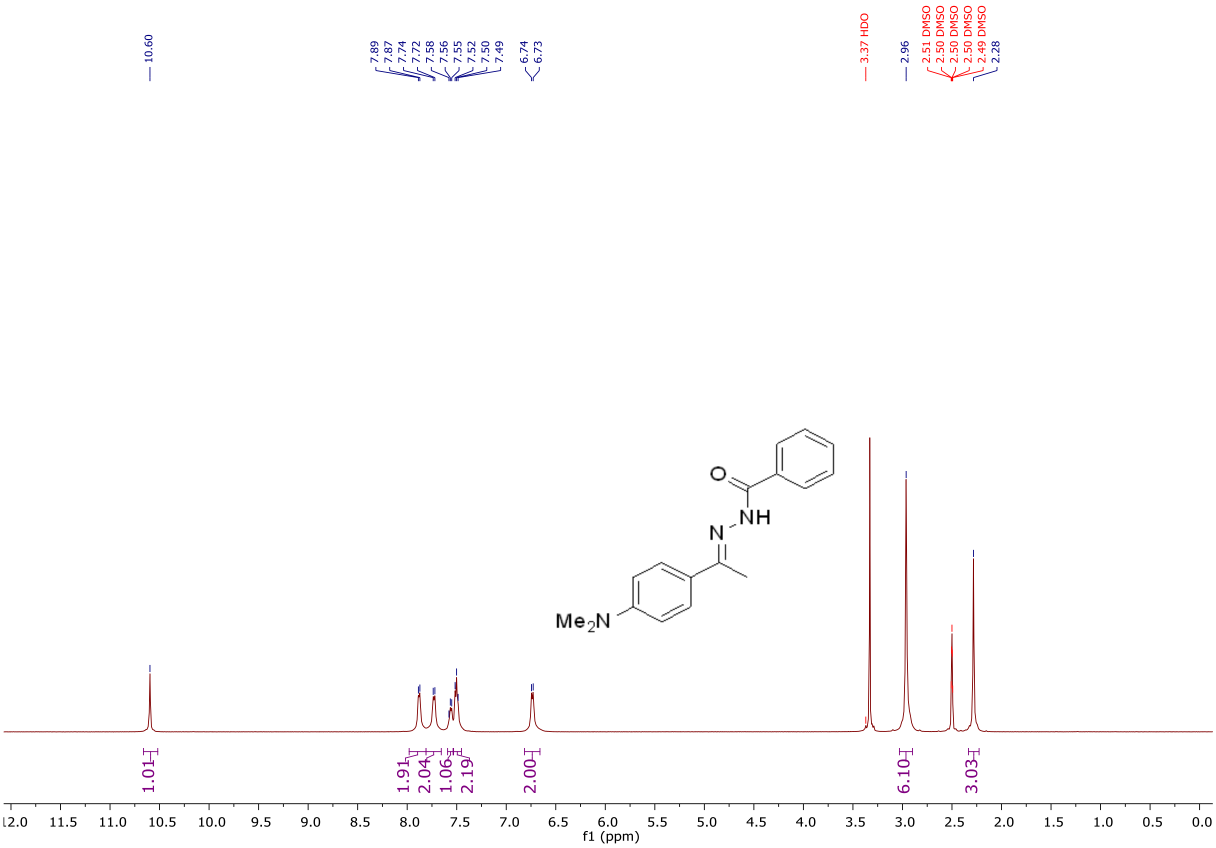

Figure S27: ^{1}H NMR of *N*'-(1-(4-(dimethylamino)phenyl)ethylidene)benzohydrazide (6l) in DMSO-d_6.

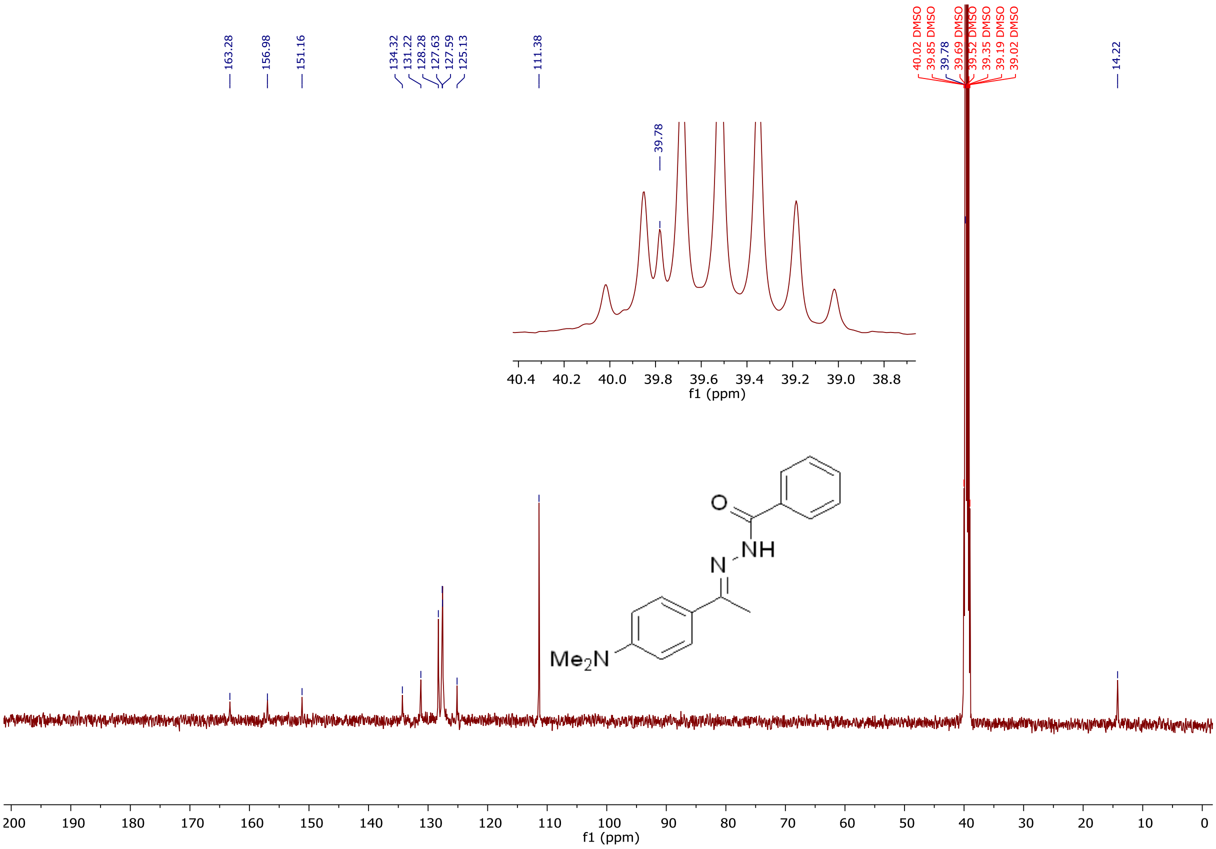

Figure S28: ^{13}C NMR of *N*'-(1-(4-(dimethylamino)phenyl)ethylidene)benzohydrazide (6l) in DMSO-*d*$_6$.

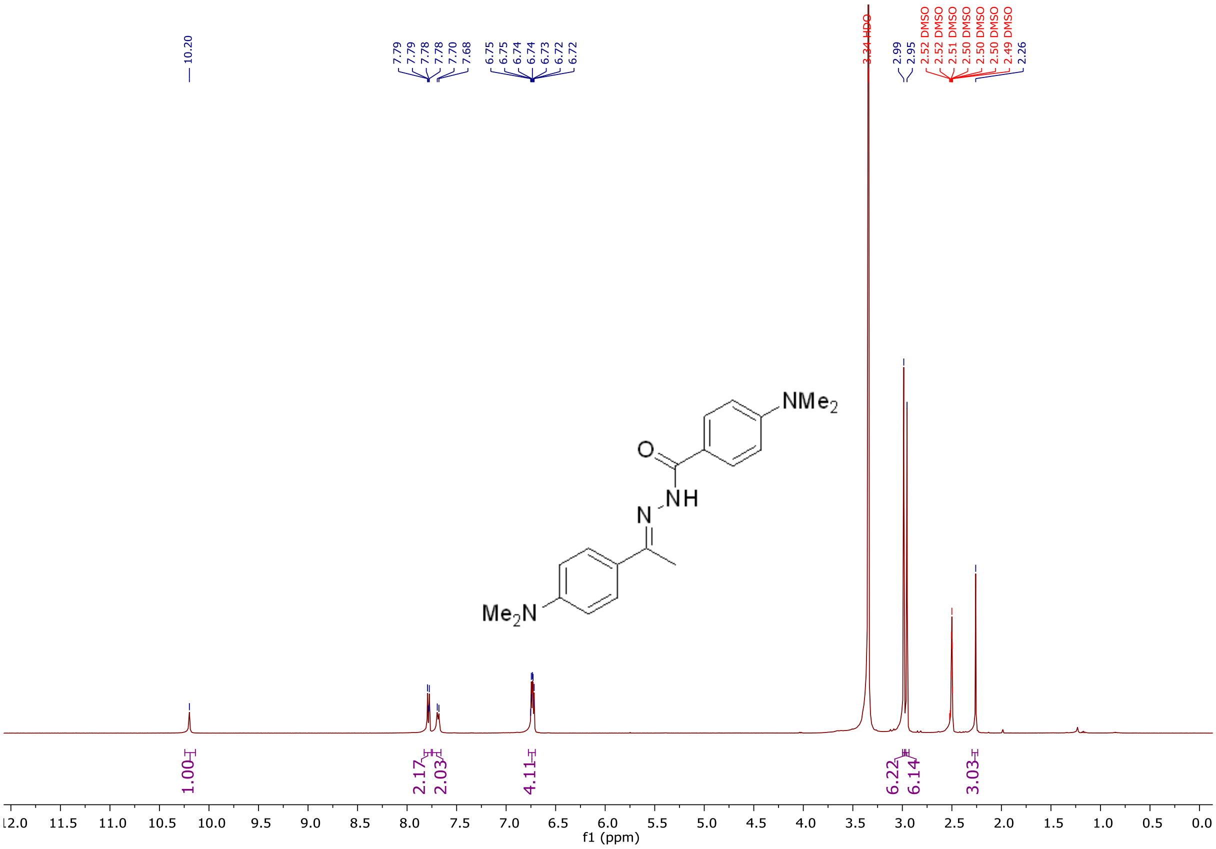

Figure S29: ^{1}H NMR of 4-(dimethylamino)-*N*'-(1-(4-(dimethylamino)phenyl)ethylidene)benzohydrazide (6m) in DMSO-*d*$_6$.

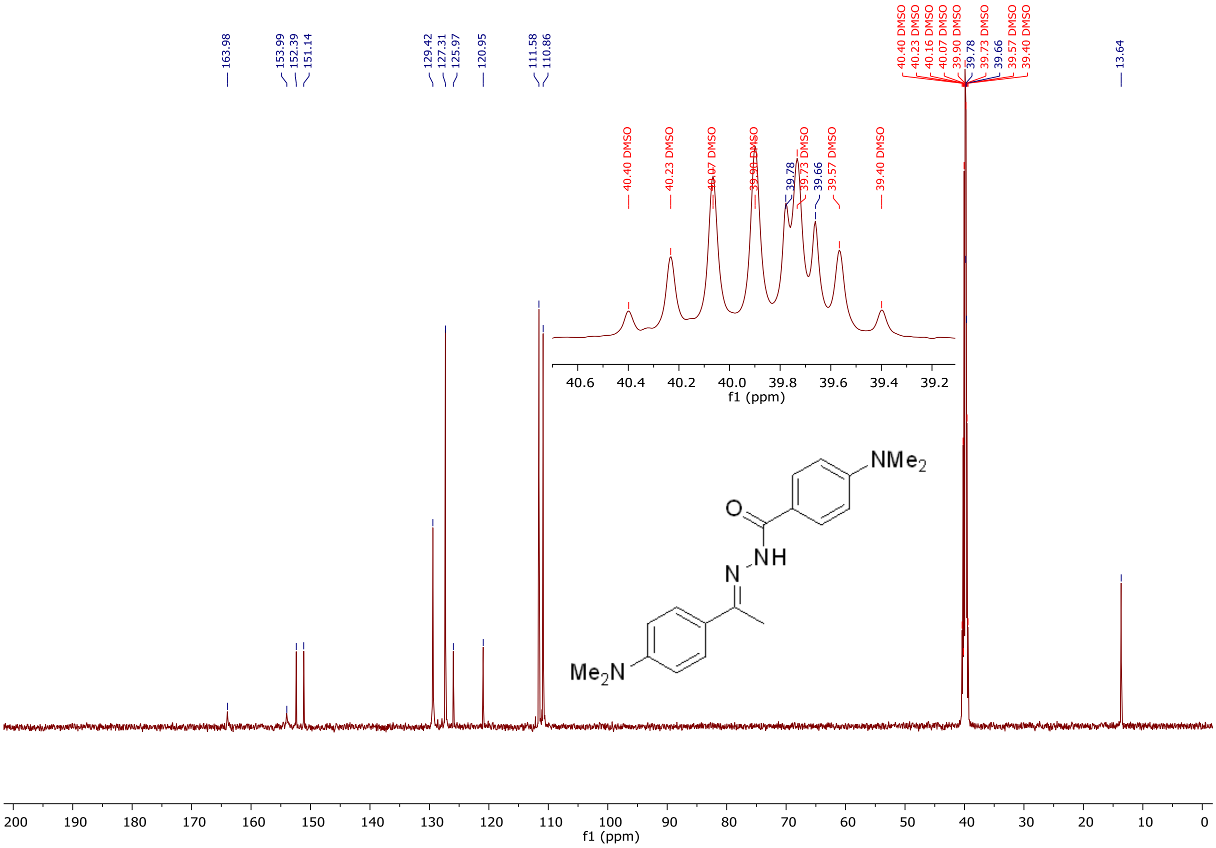

Figure S30: ^{13}C NMR of 4-(dimethylamino)-*N*'-(1-(4-(dimethylamino)phenyl)ethylidene)benzohydrazide (6m) in DMSO-d_6.

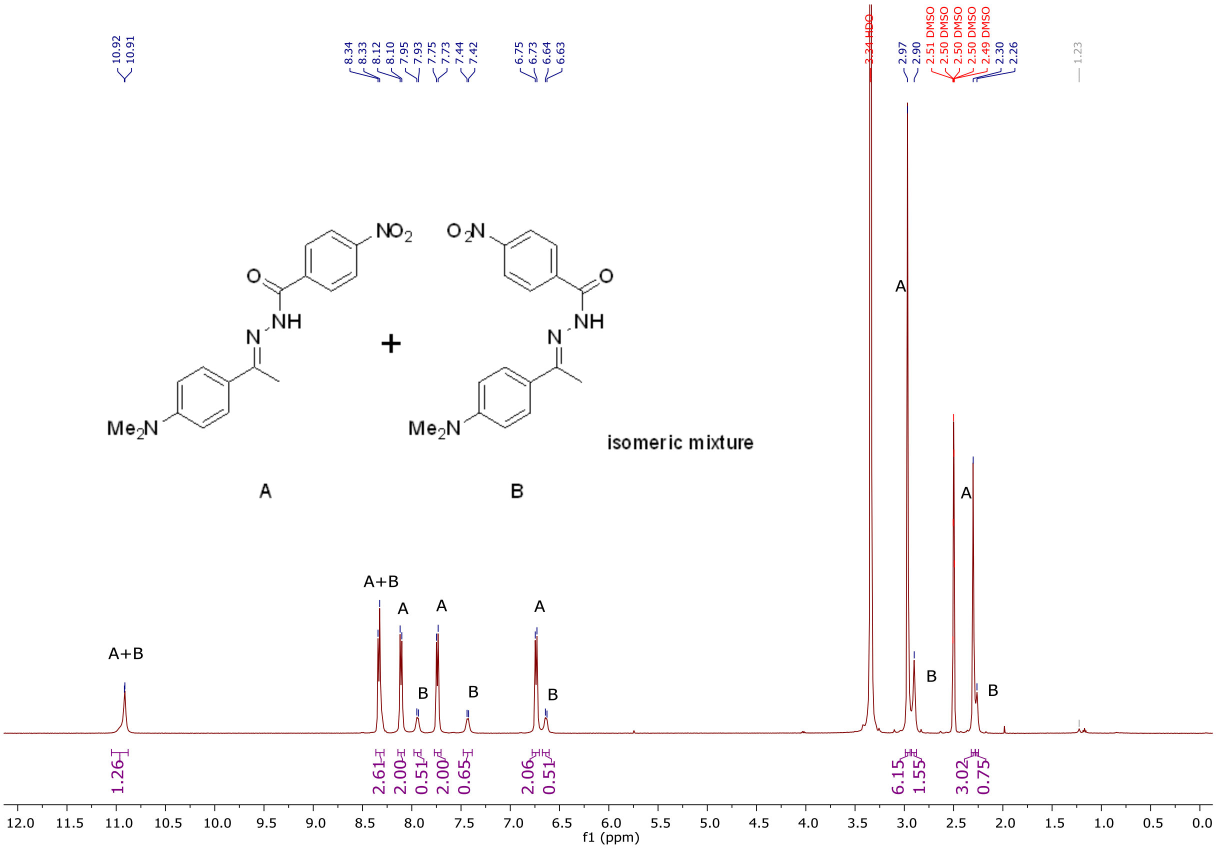

Figure S31: ^{1}H NMR of 4-nitro-*N*'-(1-(4-(dimethylamino)phenyl)ethylidene)benzohydrazide (6n) in DMSO-d_6.

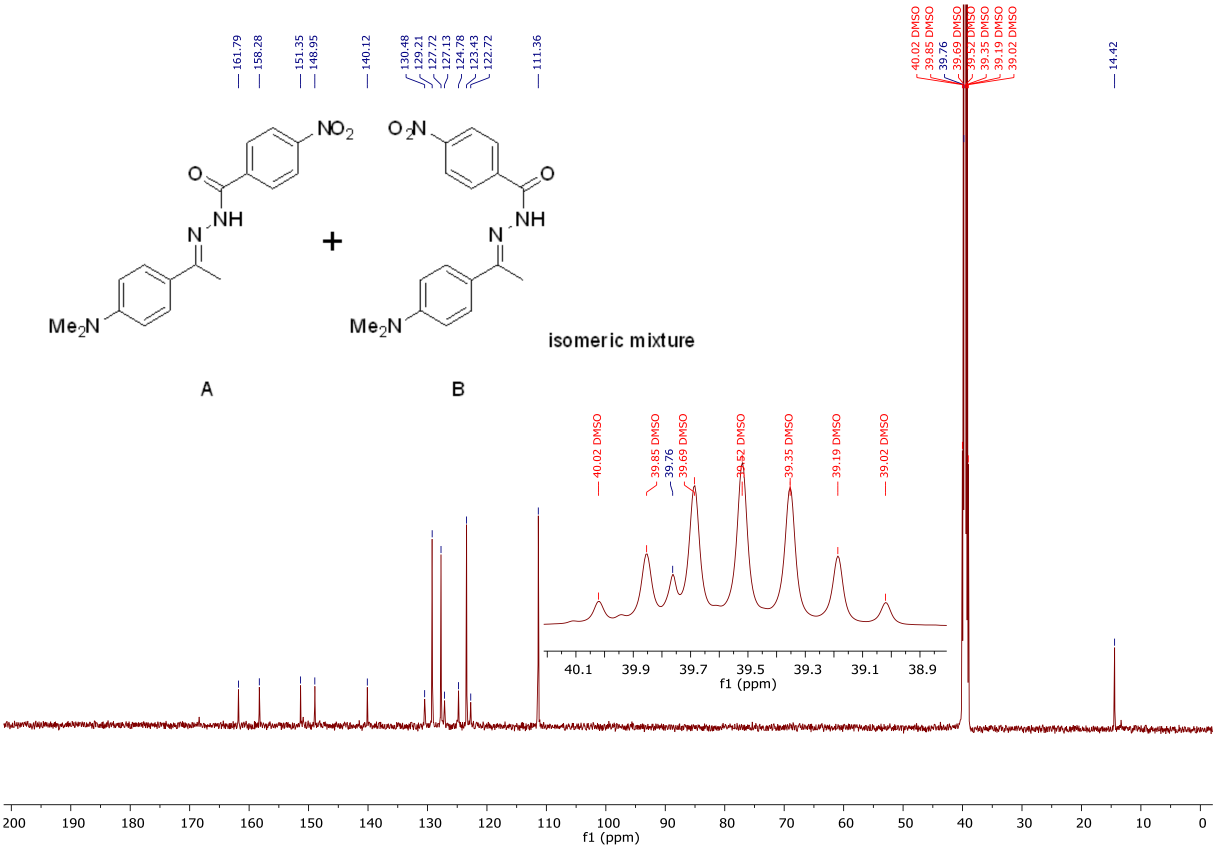

Figure S32: ^{13}C NMR of 4-nitro-*N*'-(1-(4-(dimethylamino)phenyl)ethylidene)benzohydrazide (6n) in DMSO-d_6.

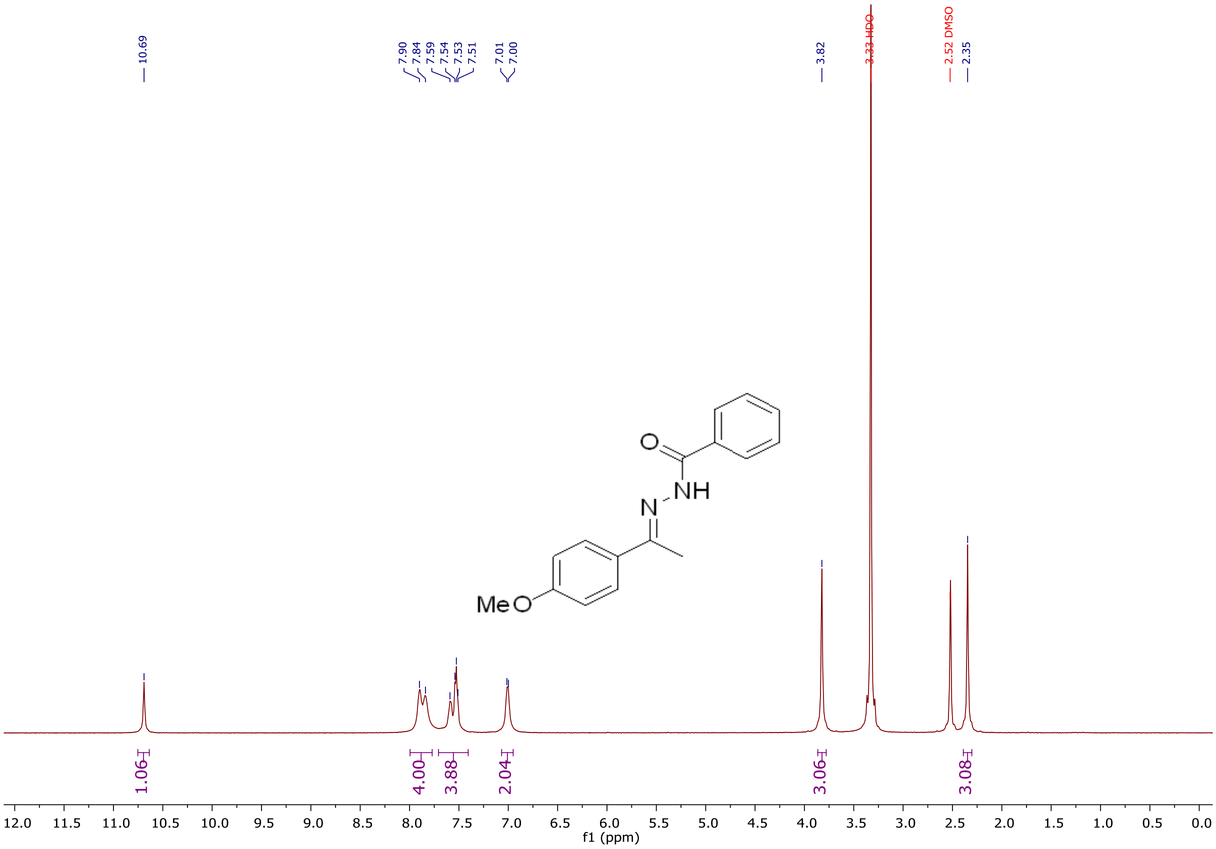

Figure S33: ^{1}H NMR of *N*'-(1-(4-methoxyphenyl)ethylidene)benzohydrazide (6o) in DMSO-d_6.

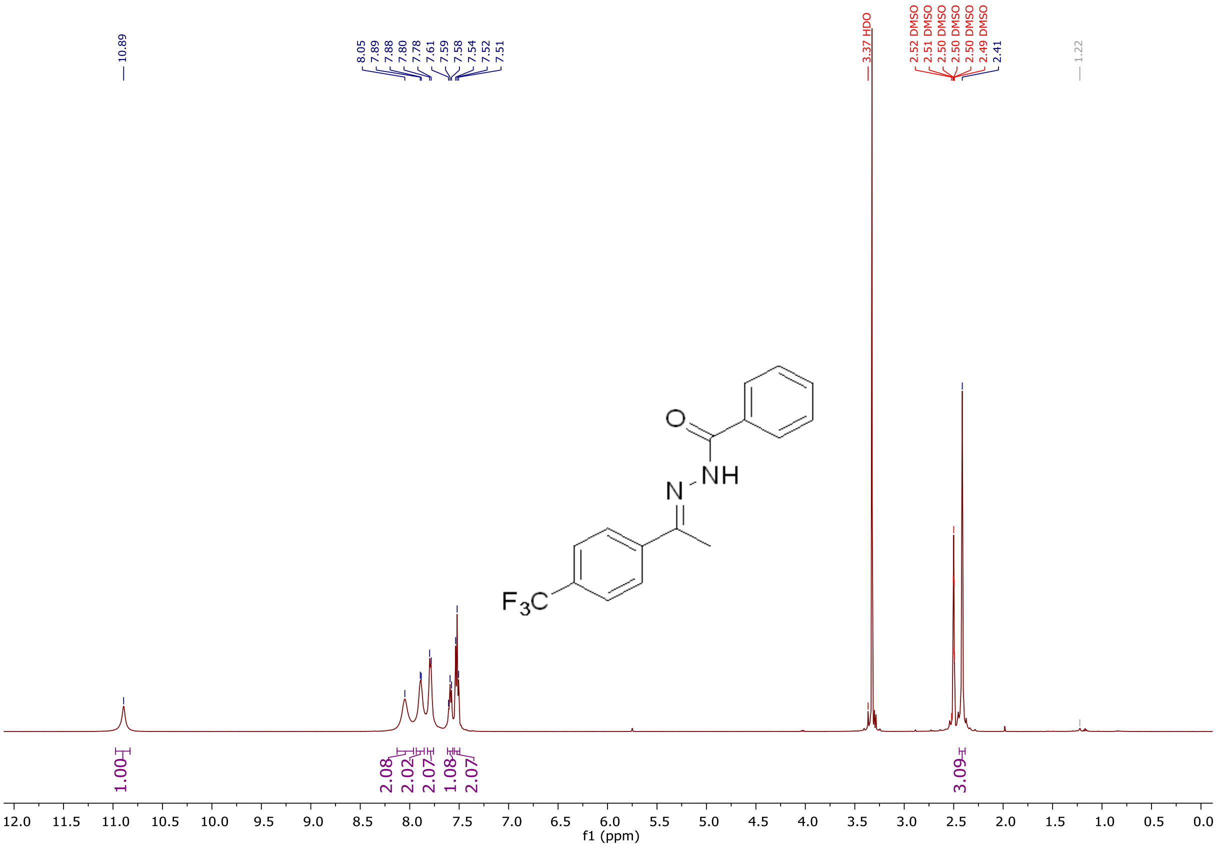

Figure S34: ^{1}H NMR of *N*'-(1-(4-(trifluoromethyl)phenyl)ethylidene)benzohydrazide (6p) in DMSO-d_6.

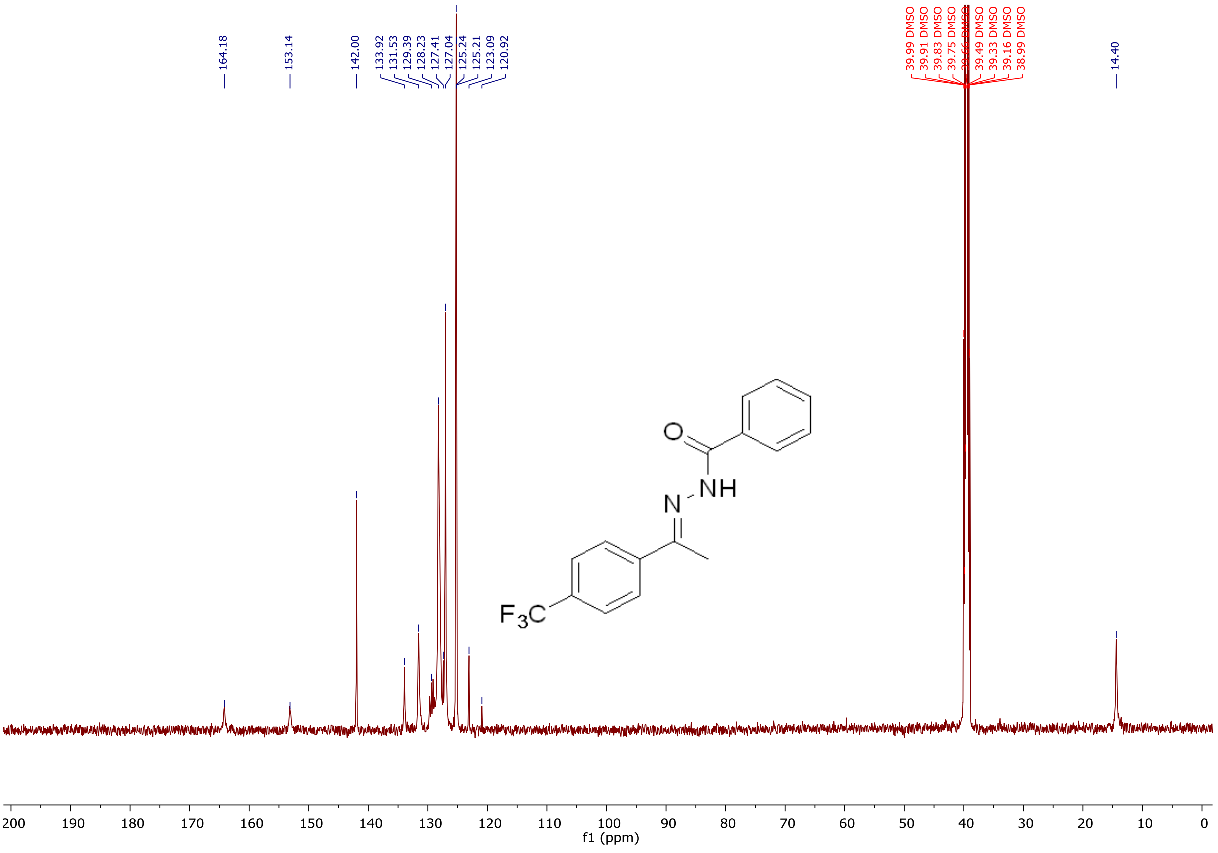

Figure S35: ^{13}C NMR of *N*'-(1-(4-(trifluoromethyl)phenyl)ethylidene)benzohydrazide (6p) in DMSO-d_6.

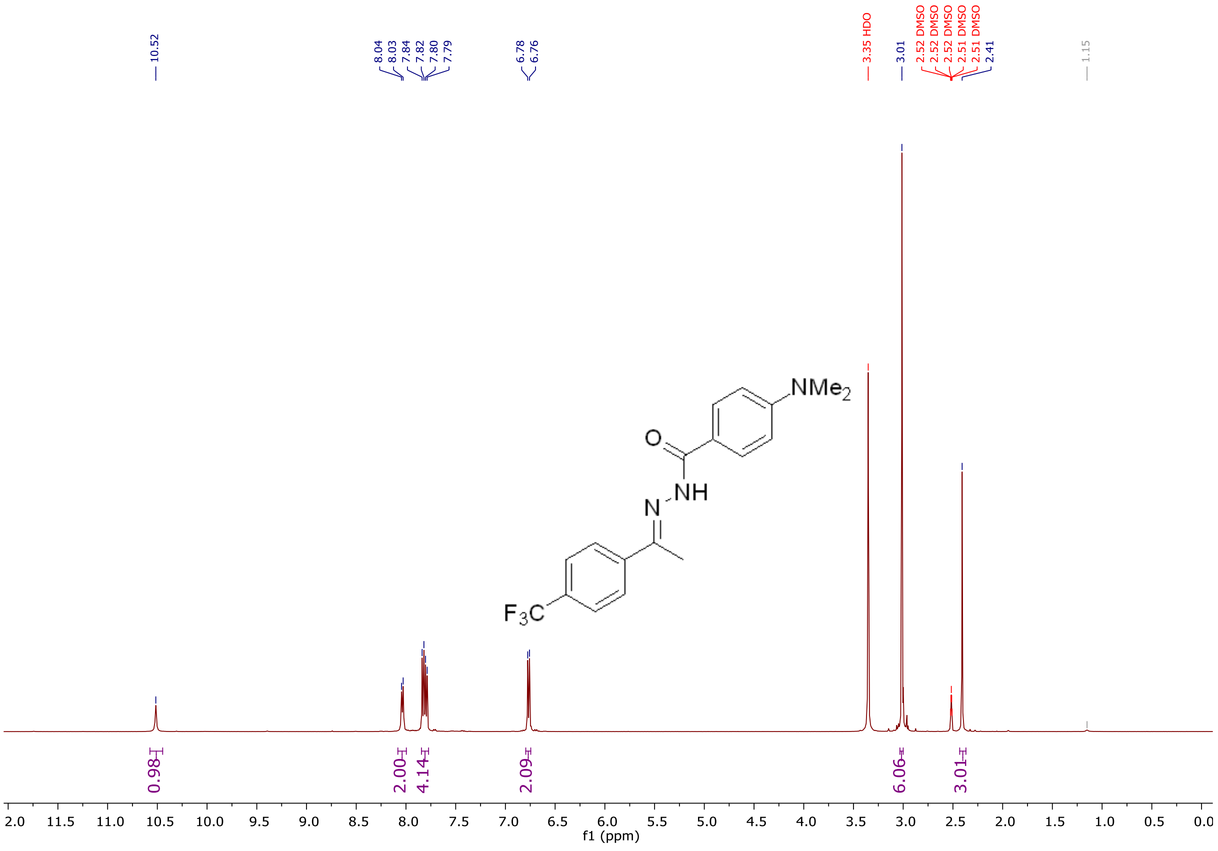

Figure S36: ^{1}H NMR of 4-(dimethylamino)-*N*'-(1-(4-(trifluoromethyl)phenyl)ethylidene)benzohydrazide (6q) in DMSO-d_6.

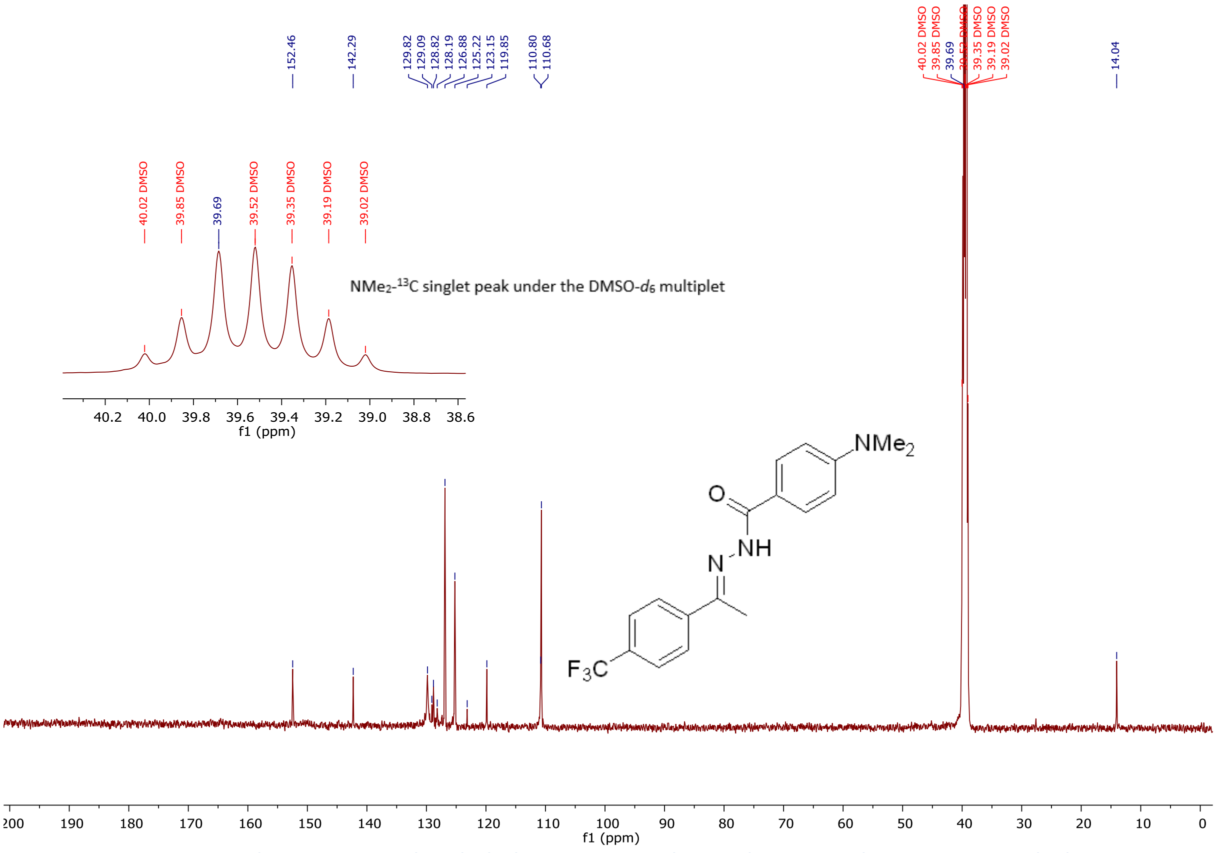

Figure S37: ^{13}C NMR of 4-(dimethylamino)-*N*'-(1-(4-(trifluoromethyl)phenyl)ethylidene)benzohydrazide (6q) in DMSO-d_6.

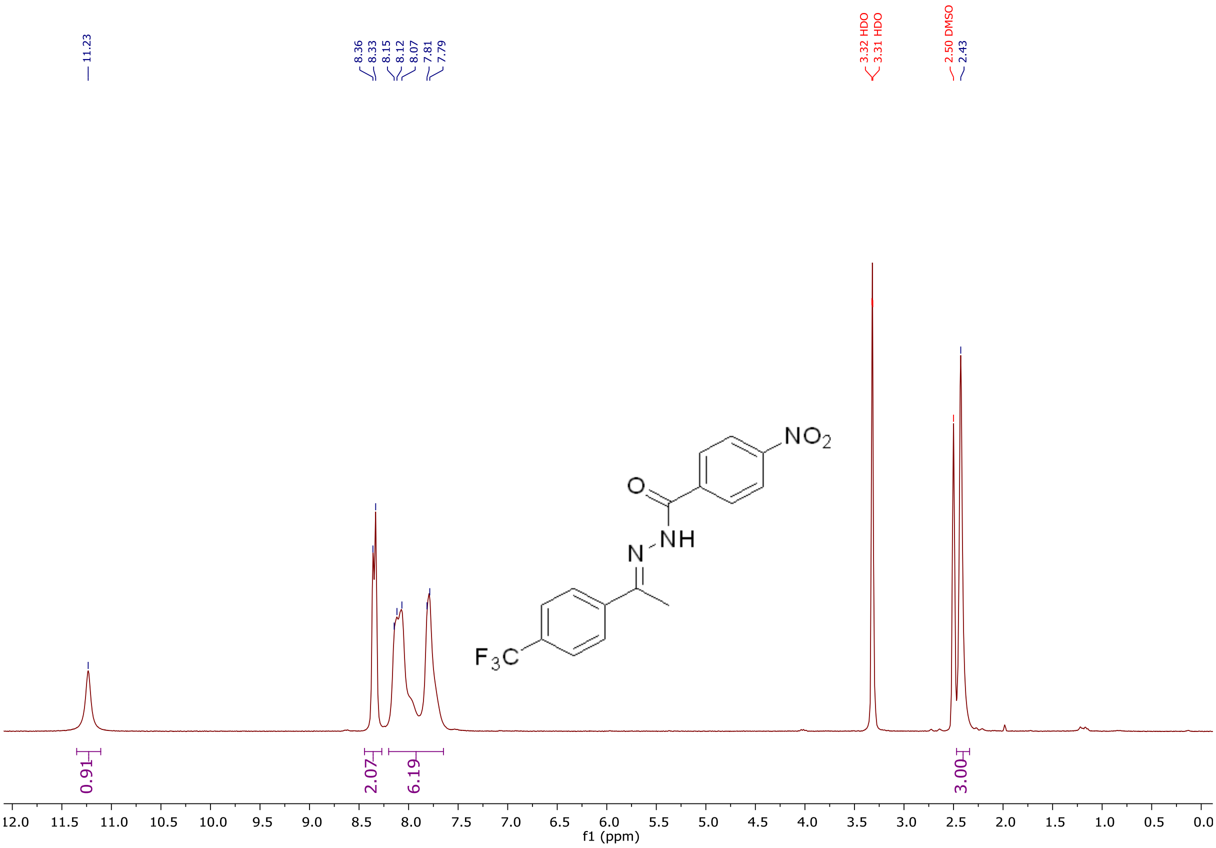

Figure S38: ^{1}H NMR of 4-nitro-*N*'-(1-(4-(trifluoromethyl)phenyl)ethylidene)benzohydrazide (6r) in DMSO-*d*$_6$.

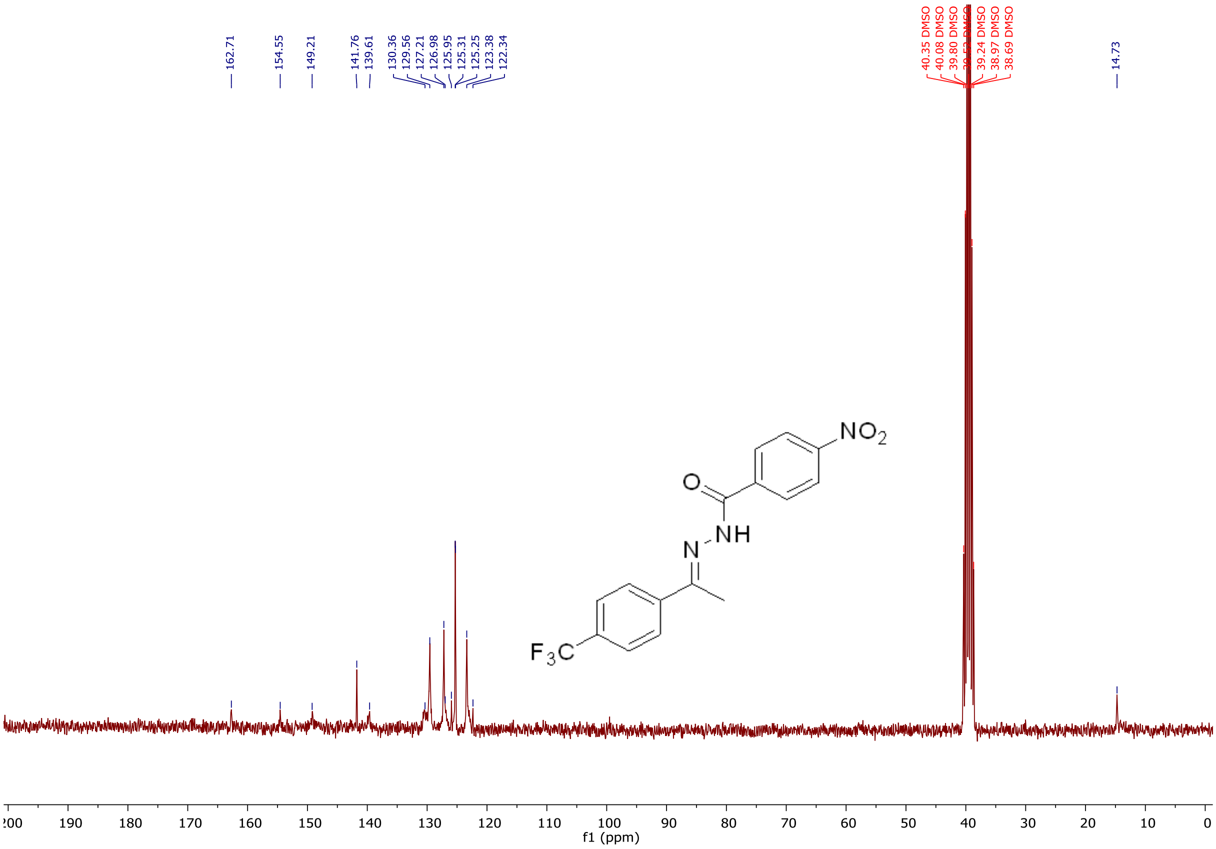

Figure S39: ^{13}C NMR of 4-nitro-*N*'-(1-(4-(trifluoromethyl)phenyl)ethylidene)benzohydrazide (6r) in DMSO-*d*$_6$.

2.2.2. Internal alkynes as substrates

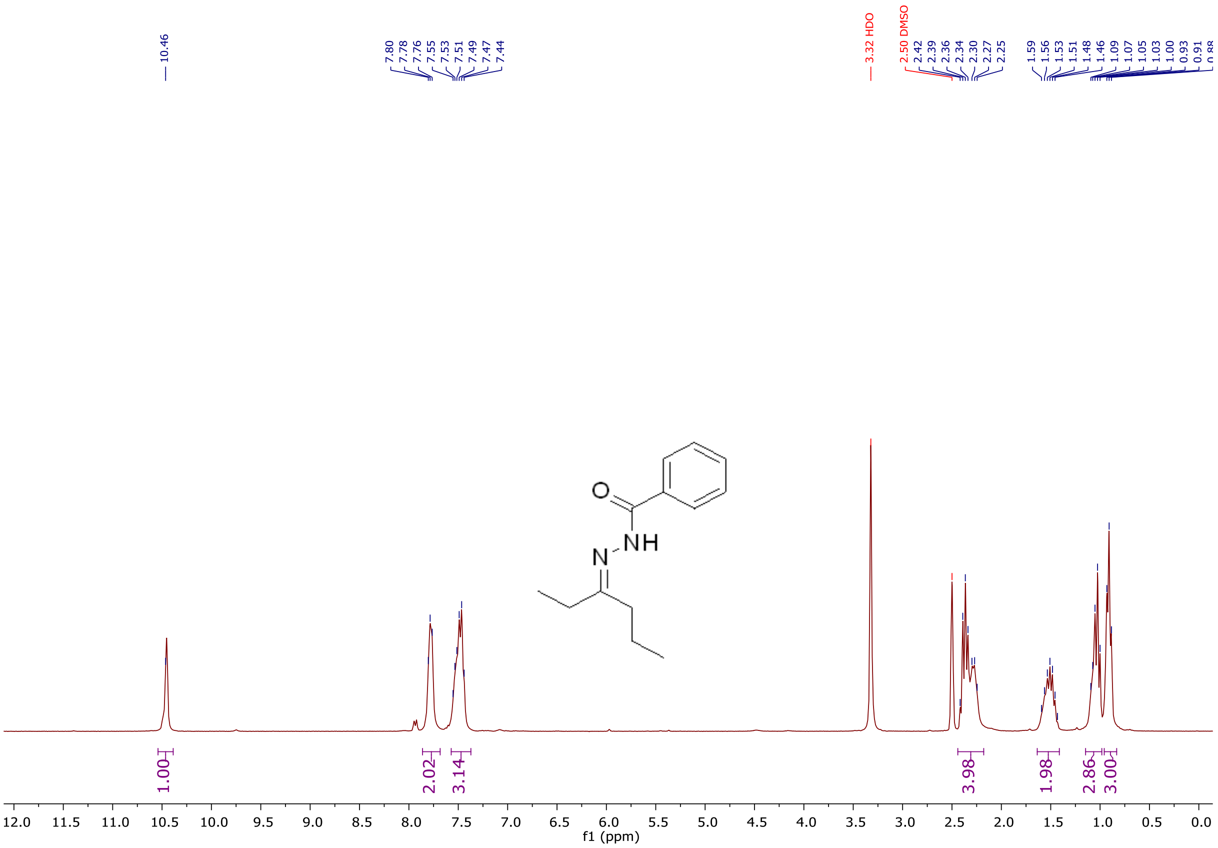

Figure S40: ^{1}H NMR of *N*'-(hexan-3-ylidene)benzohydrazide (7a) in DMSO-*d*$_6$.

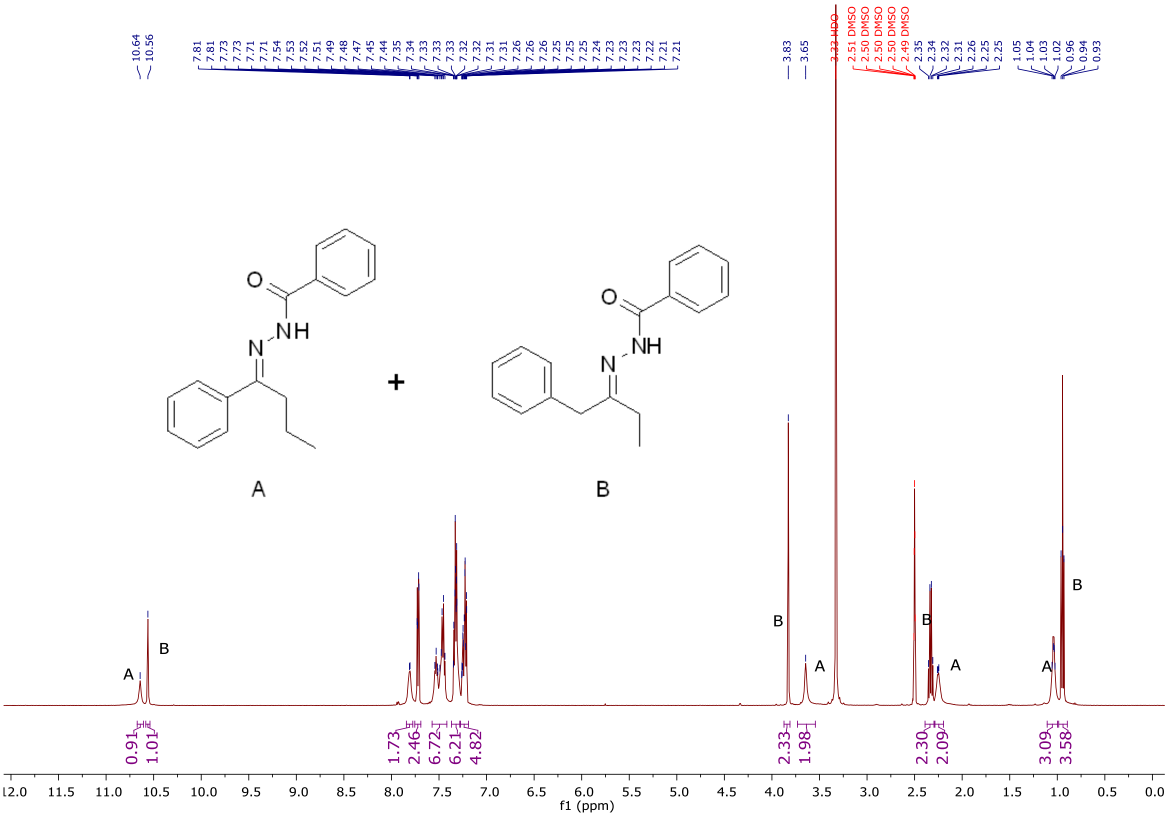

Figure S41: ^{1}H NMR of *N*'-(1-phenylbutylidene)benzohydrazide and *N*'-(1-phenylbutan-2-ylidene)benzohydrazide (7b) in DMSO-d_6.

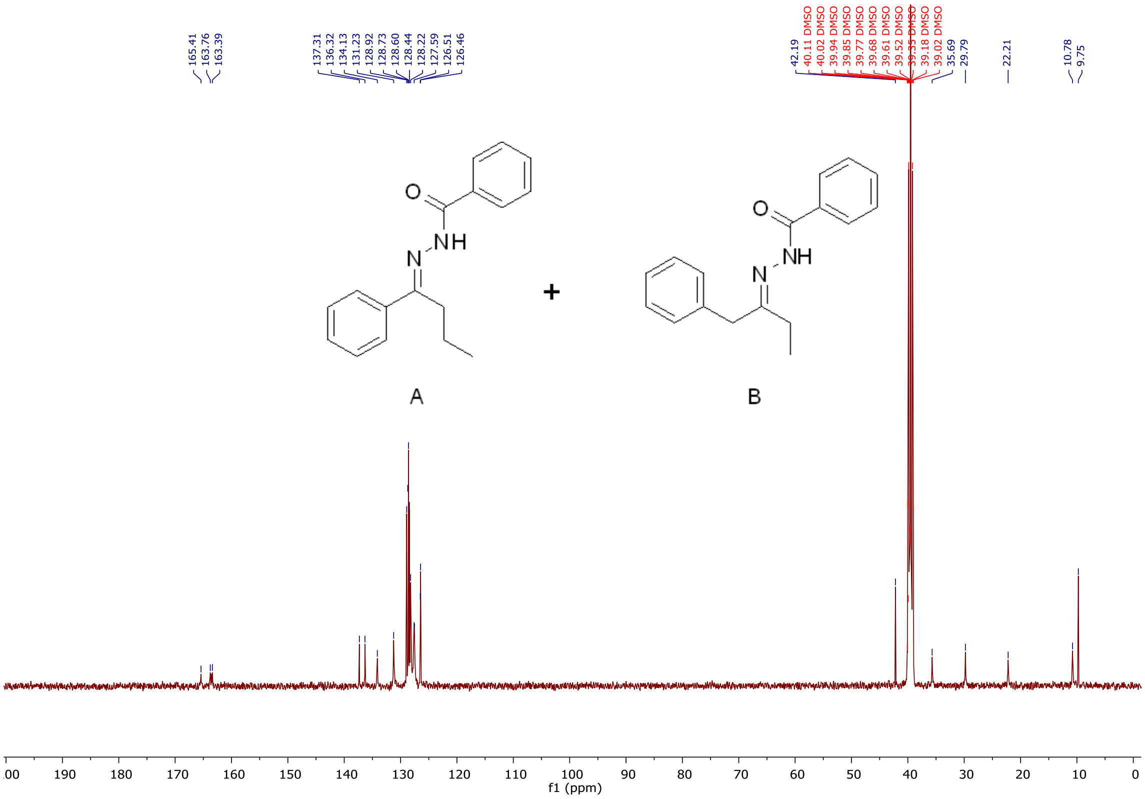

Figure S42: ^{13}C NMR of *N*'-(1-phenylbutylidene)benzohydrazide and *N*'-(1-phenylbutan-2-ylidene)benzohydrazide (7b) in DMSO-d_6.

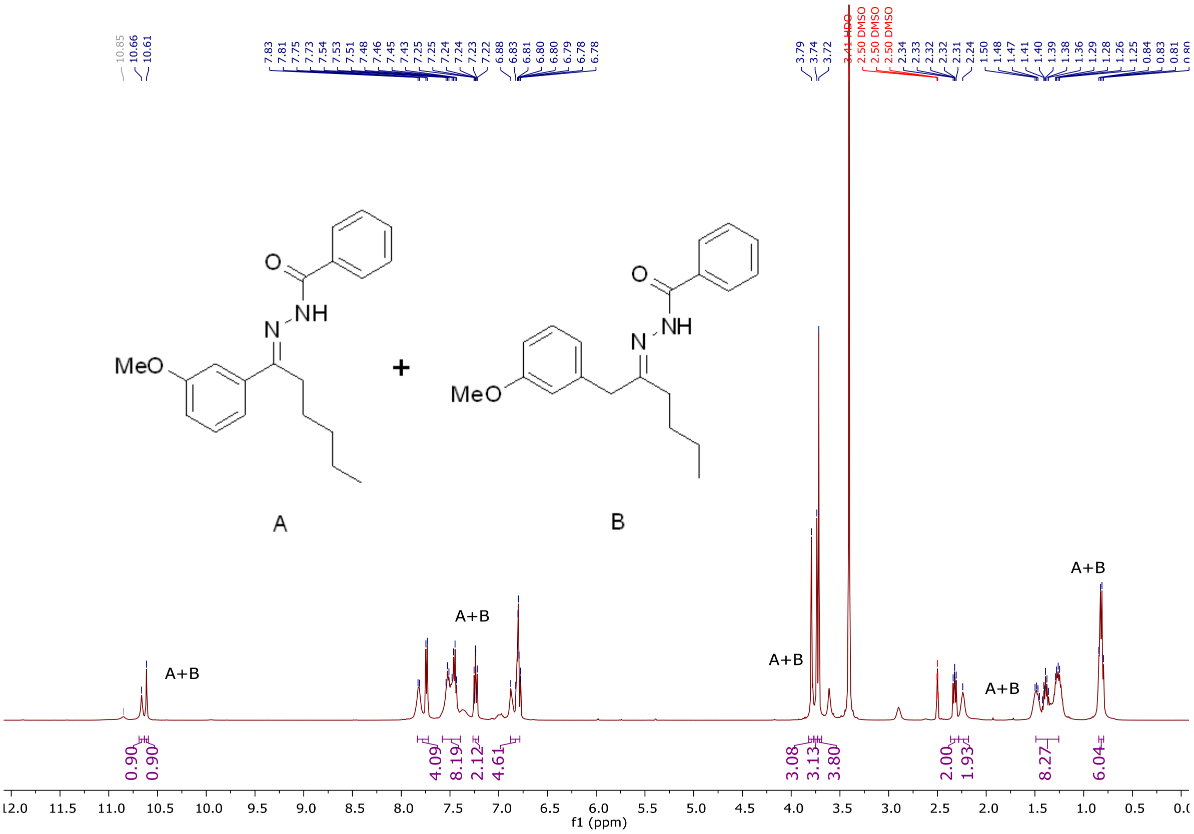

Figure S43: ^{1}H NMR of *N*'-(1-(3-methoxyphenyl)hexylidene)benzohydrazide and *N*'-(1-(3-methoxyphenyl)hexan-2-ylidene)benzohydrazide (7c) in DMSO-d_6.

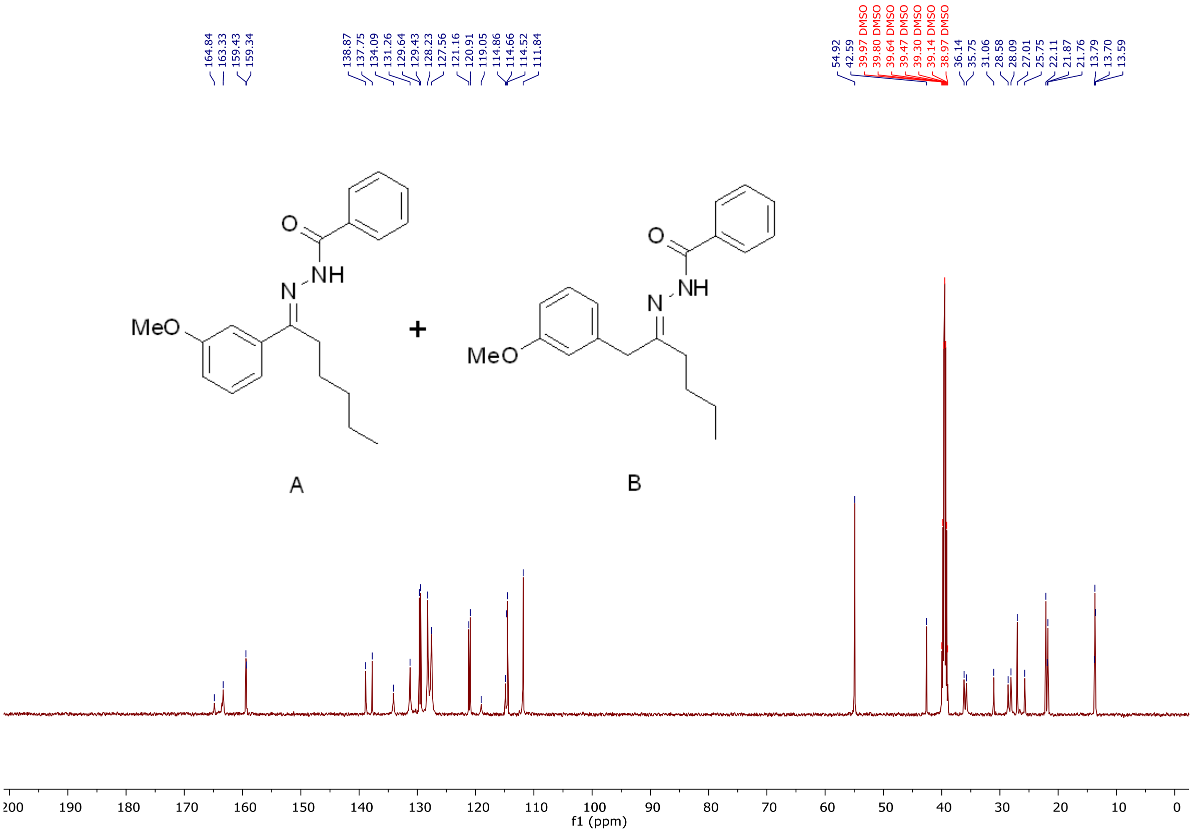

Figure S44: ^{13}C NMR of *N*'-(1-(3-methoxyphenyl)hexylidene)benzohydrazide and *N*'-(1-(3-methoxyphenyl)hexan-2-ylidene)benzohydrazide (7c) in DMSO-d_6.

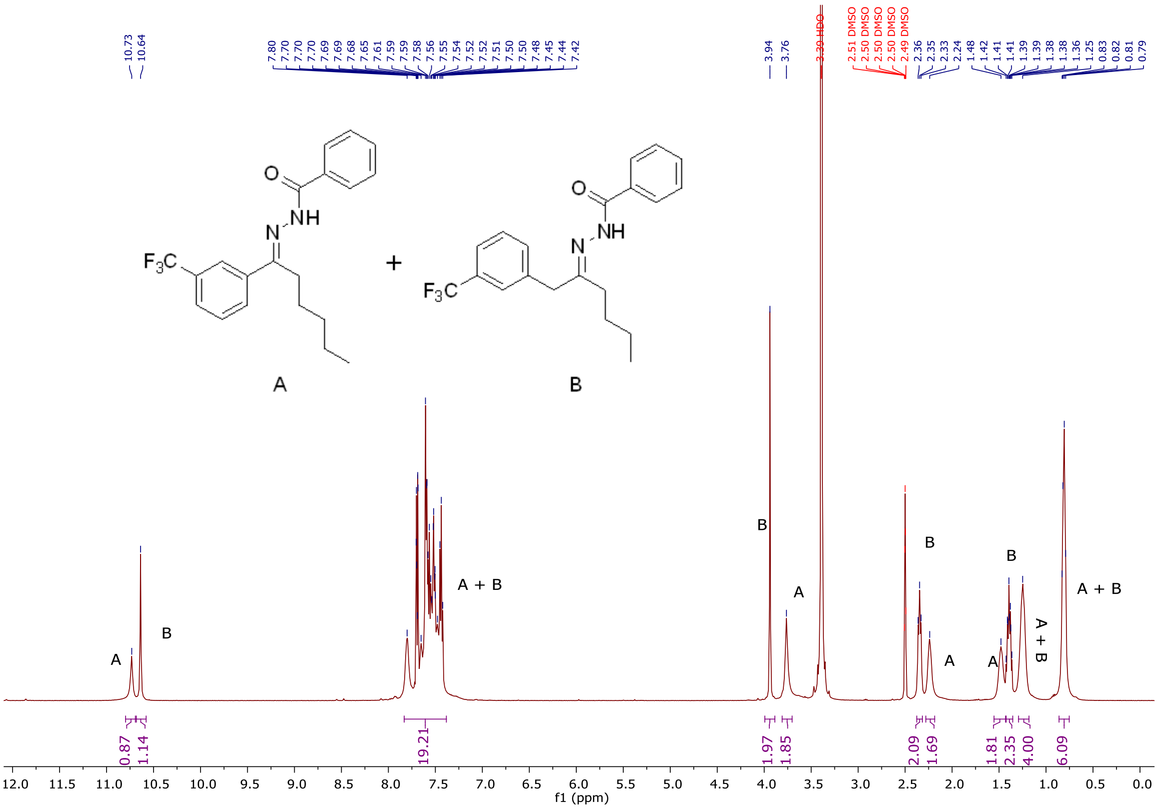

Figure S45: ^{1}H NMR of *N*'-(1-(3-(trifluoromethyl)phenyl)hexylidene)benzohydrazide and *N*'-(1-(3-(trifluoromethyl)phenyl)hexan-2-ylidene)benzohydrazide (7d) in DMSO-d_6.

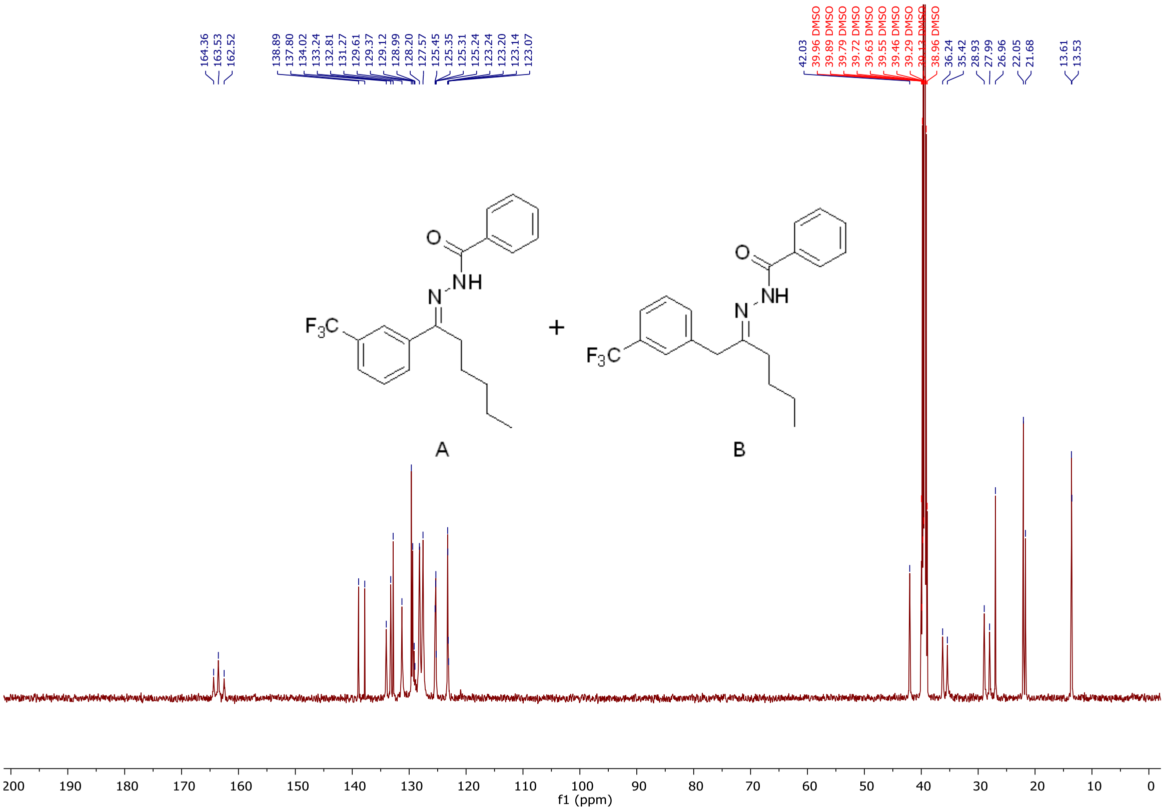

Figure S46: ^{13}C NMR of *N*'-(1-(3-(trifluoromethyl)phenyl)hexylidene)benzohydrazide and *N*'-(1-(3-(trifluoromethyl)phenyl)hexan-2-ylidene)benzohydrazide (7d) in DMSO-d_6.

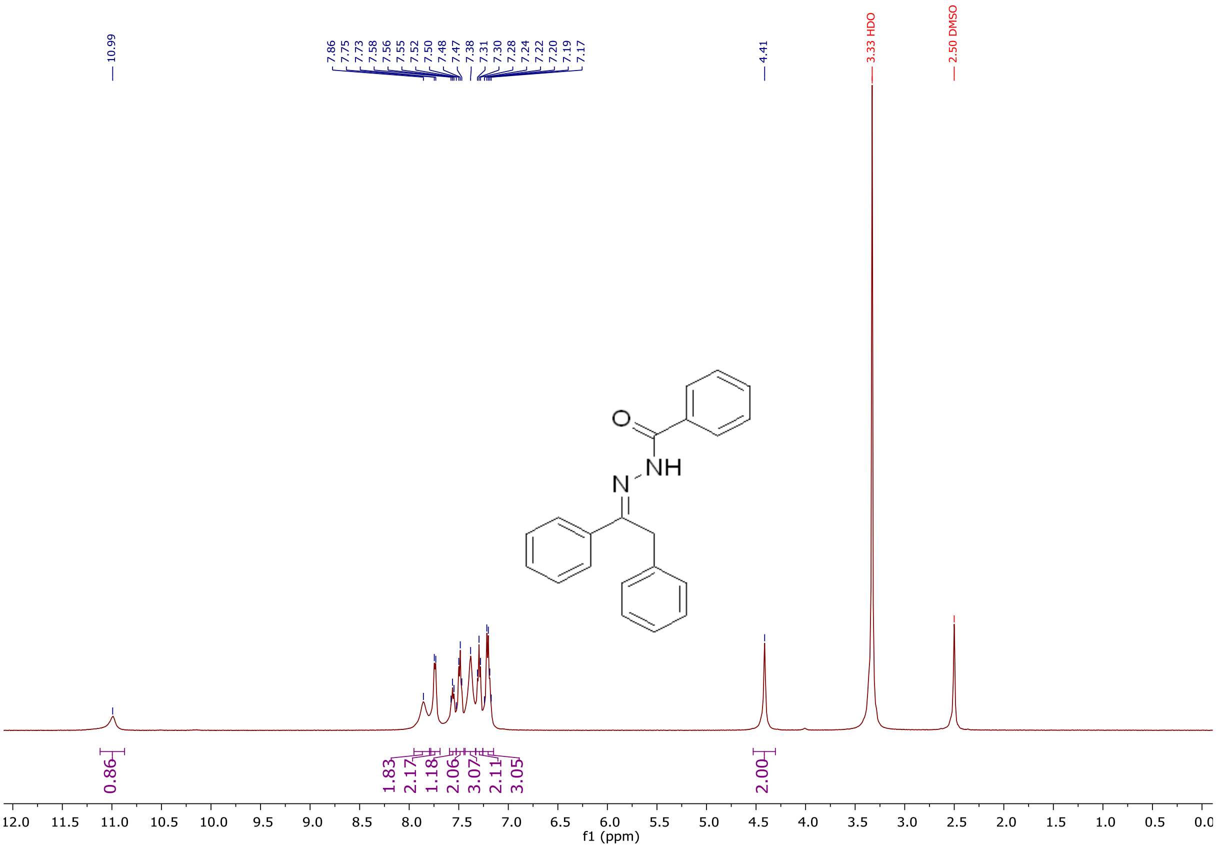

Figure S47: ^{1}H NMR of *N*'-(1,2-diphenylethylidene)benzohydrazide (7e) in DMSO-*d*$_6$.

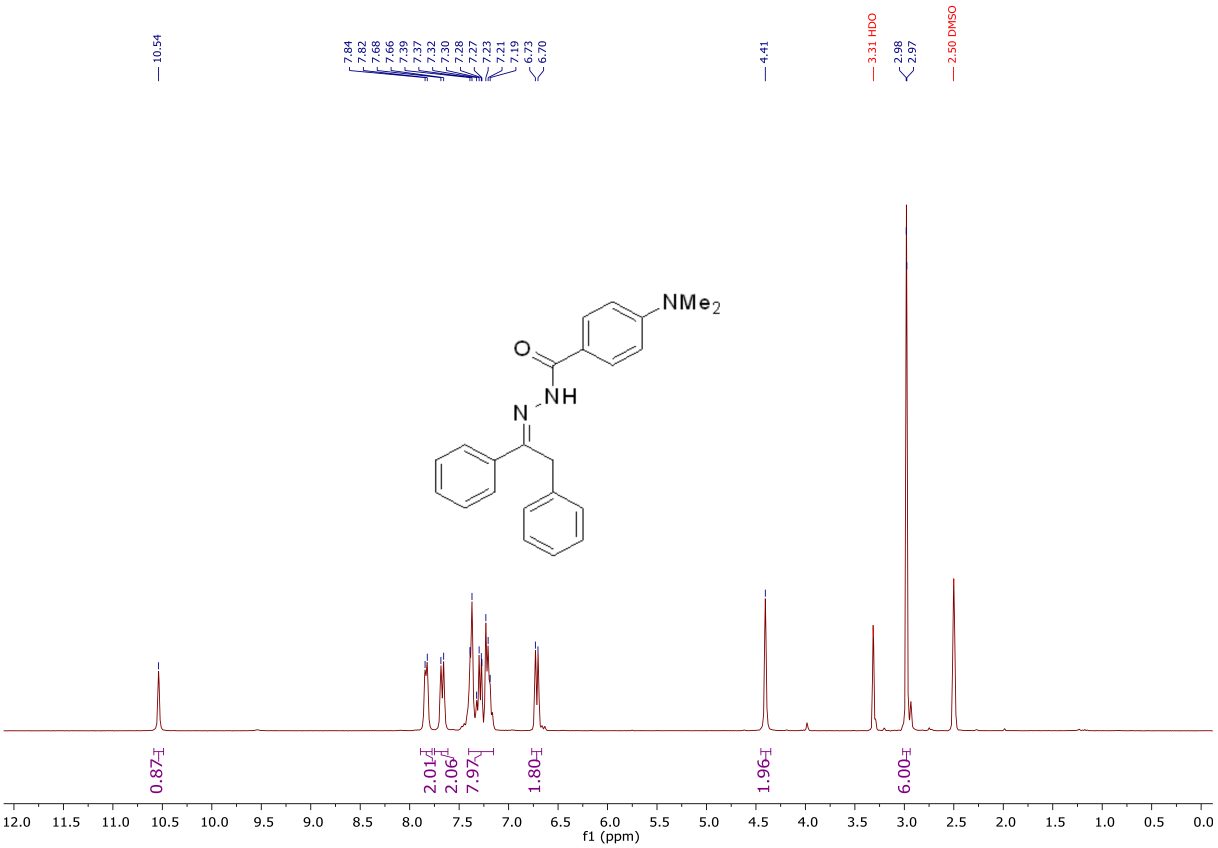

Figure S48: ^{1}H NMR of 4-(dimethylamino)-*N*'-(1,2-diphenylethylidene)benzohydrazide (7f) in DMSO-*d*$_6$.

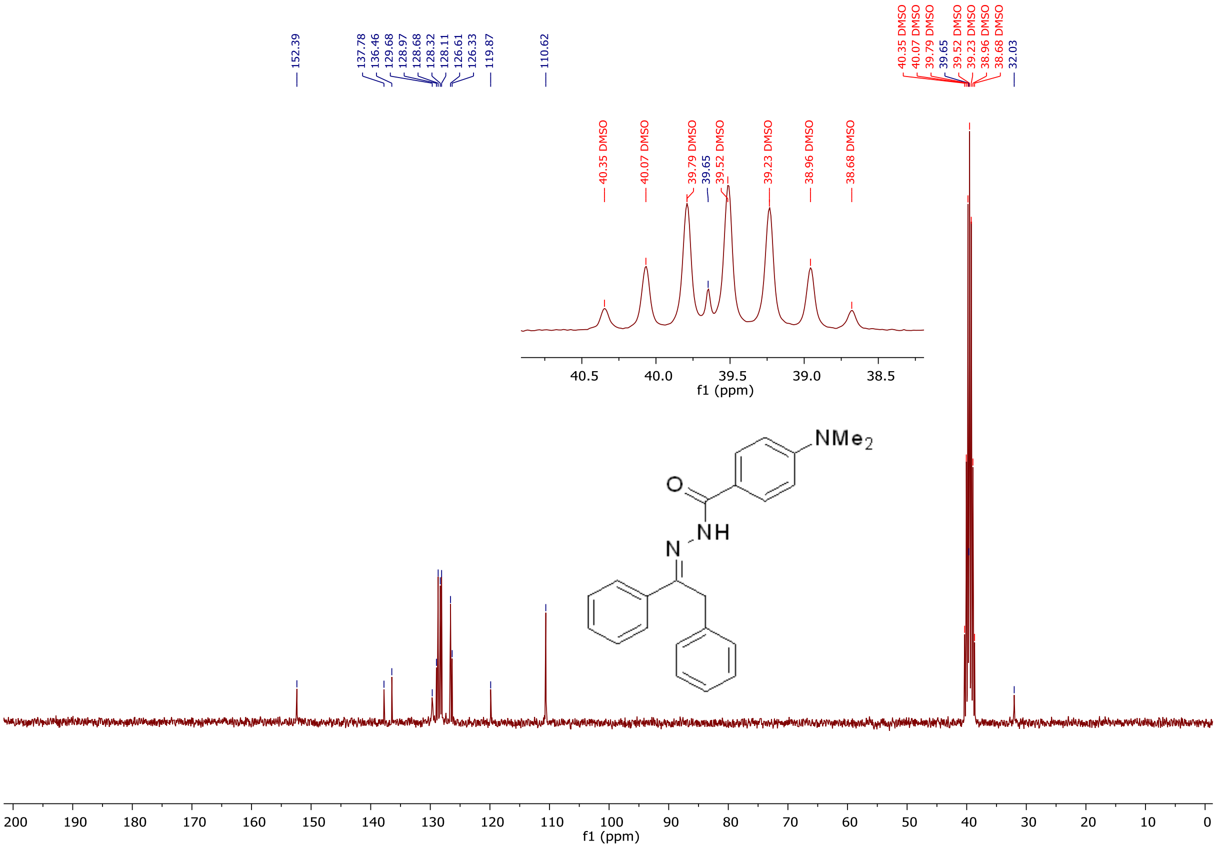

Figure S49: ^{13}C NMR of 4-(dimethylamino)-*N*'-(1,2-diphenylethylidene)benzohydrazide (7f) in DMSO-d_6.

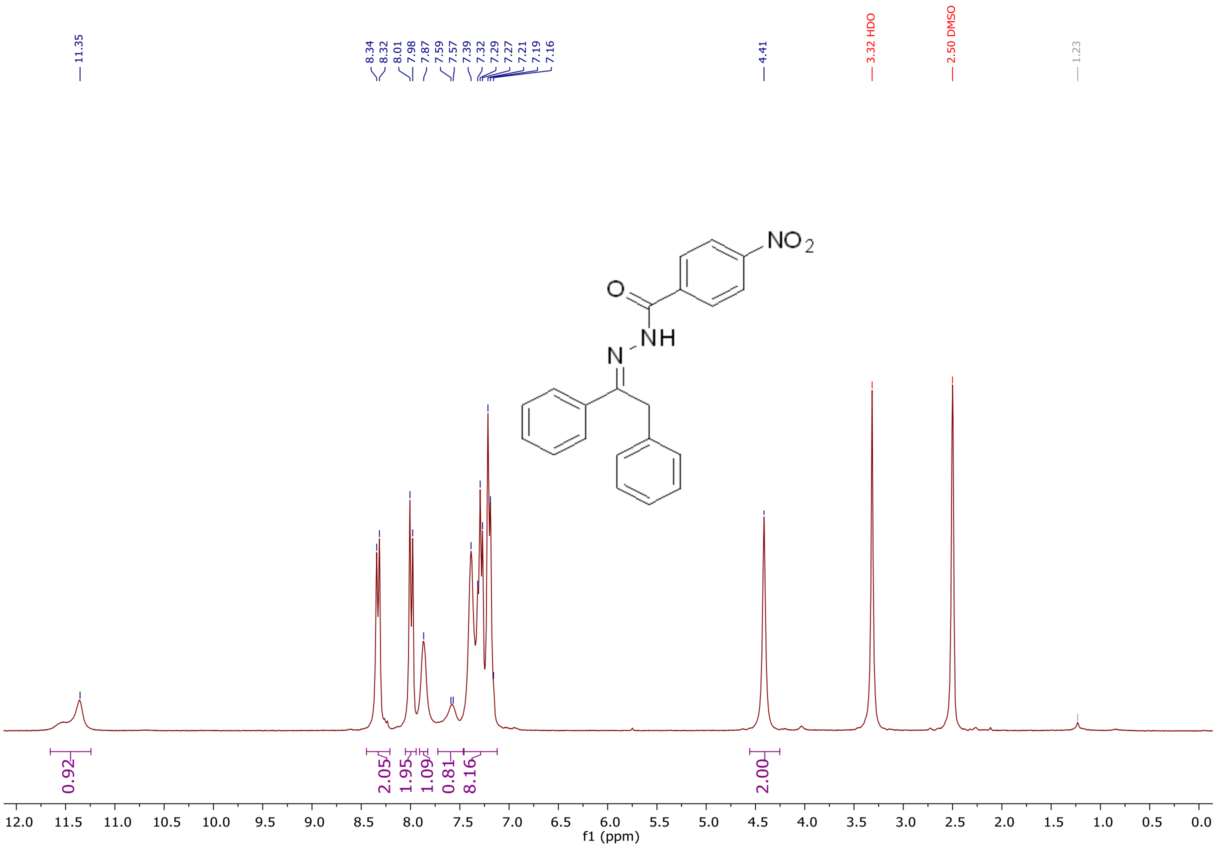

Figure S50: ^{1}H NMR of 4-nitro-*N*'-(1,2-diphenylethylidene)benzohydrazide (7g) in DMSO-d_6.

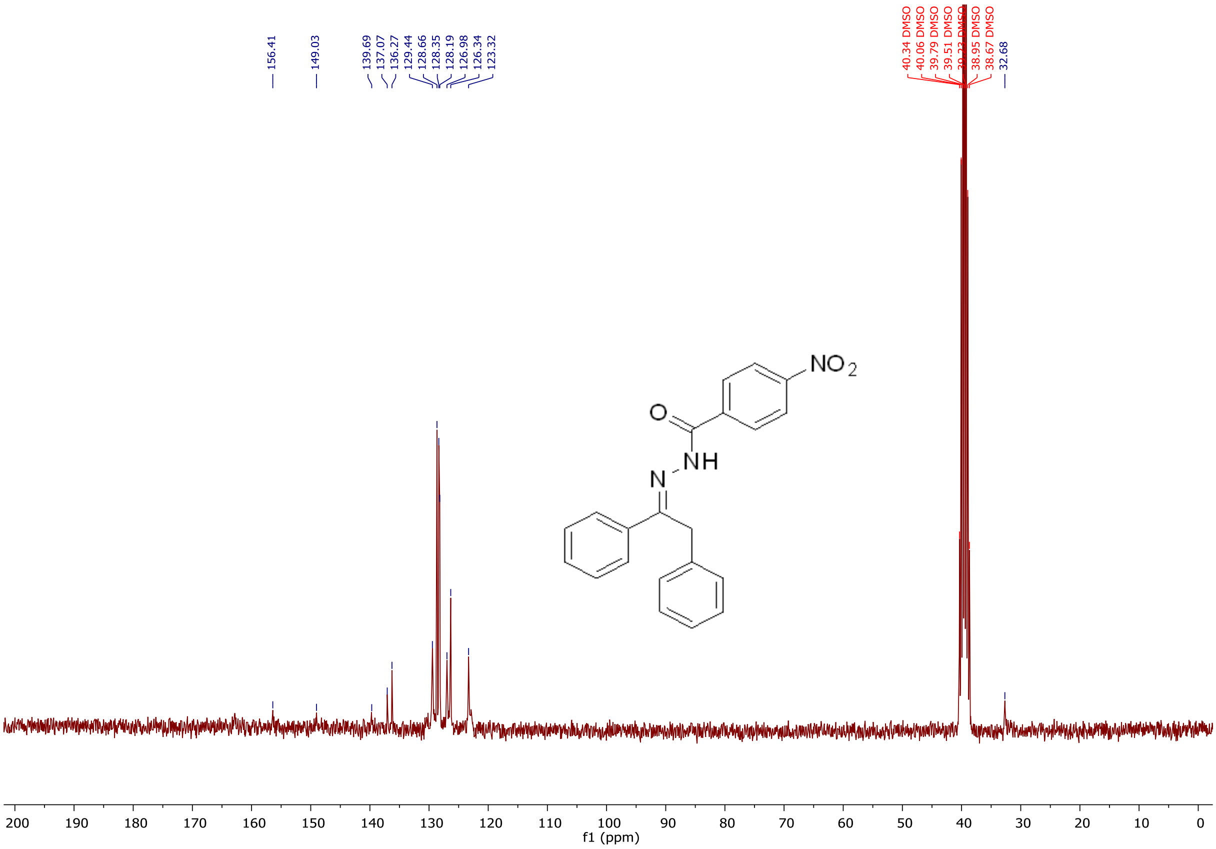

Figure S51: ^{13}C NMR of 4-nitro-*N*'-(1,2-diphenylethylidene)benzohydrazide (7g) in DMSO-*d*$_6$.

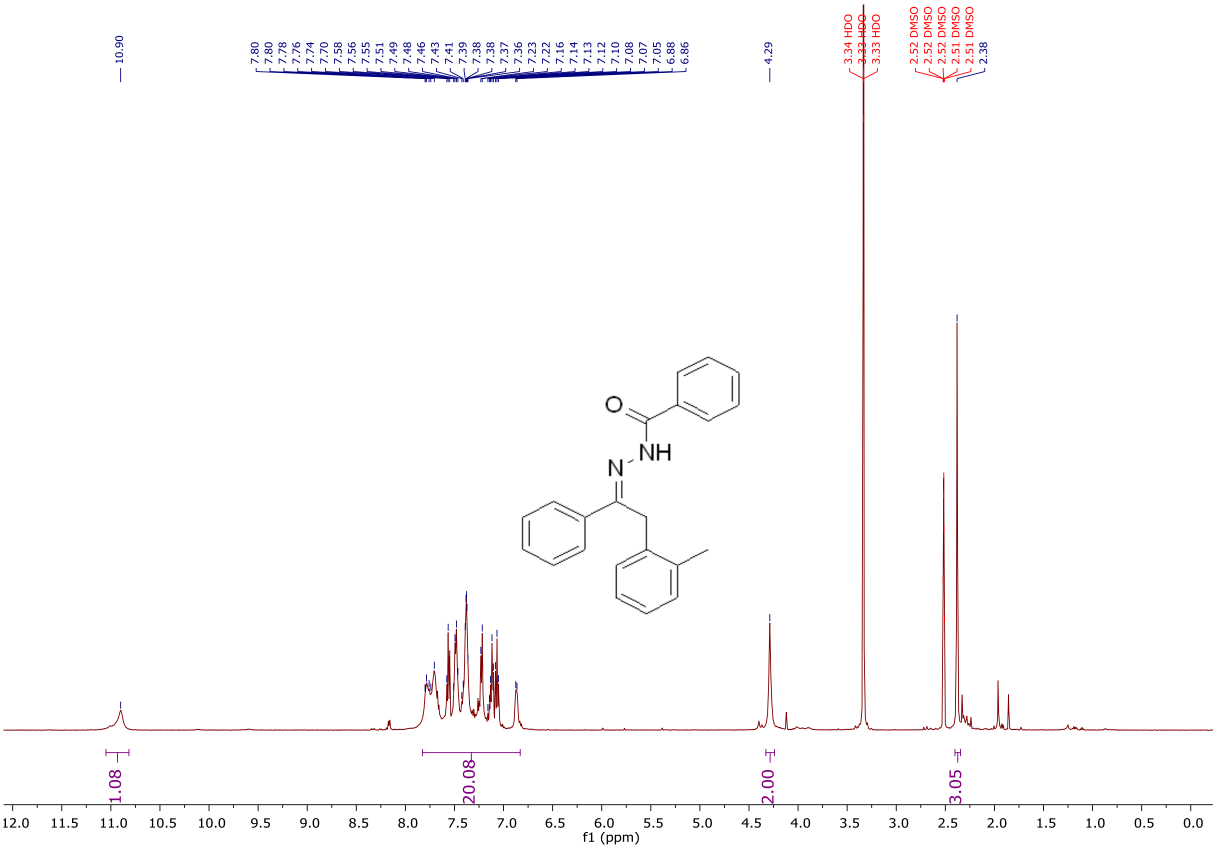

Figure S52: ^{1}H NMR of *N*'-(2-phenyl-1-(*o*-tolyl)ethylidene)benzohydrazide and *N*'-(1-phenyl-2-(*o*-tolyl)ethylidene)-benzohydrazide (7h) in DMSO-*d*$_6$.

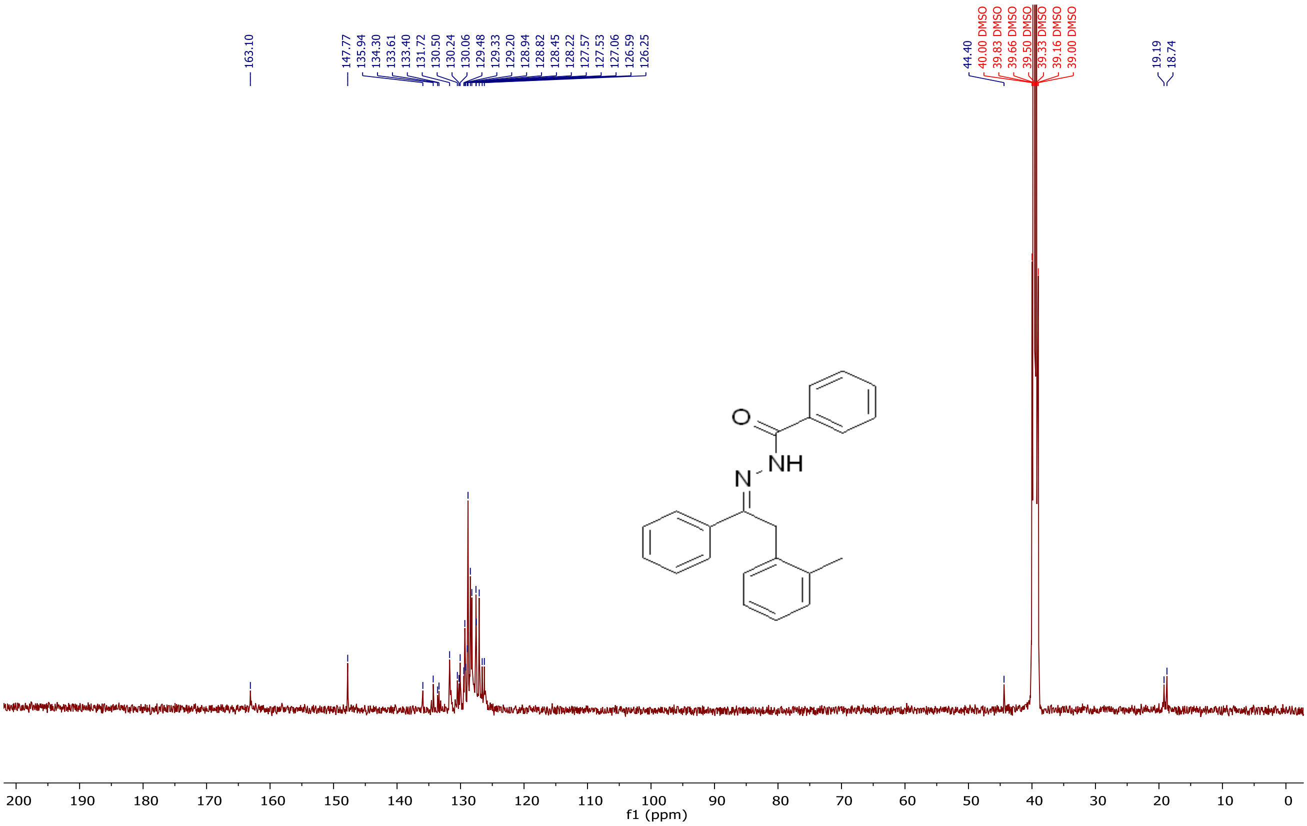

Figure S53: ^{13}C NMR of *N*'-(2-phenyl-1-(*o*-tolyl)ethylidene)benzohydrazide and *N*'-(1-phenyl-2-(*o*-tolyl)ethylidene)-benzohydrazide (7h) in DMSO-d_6.

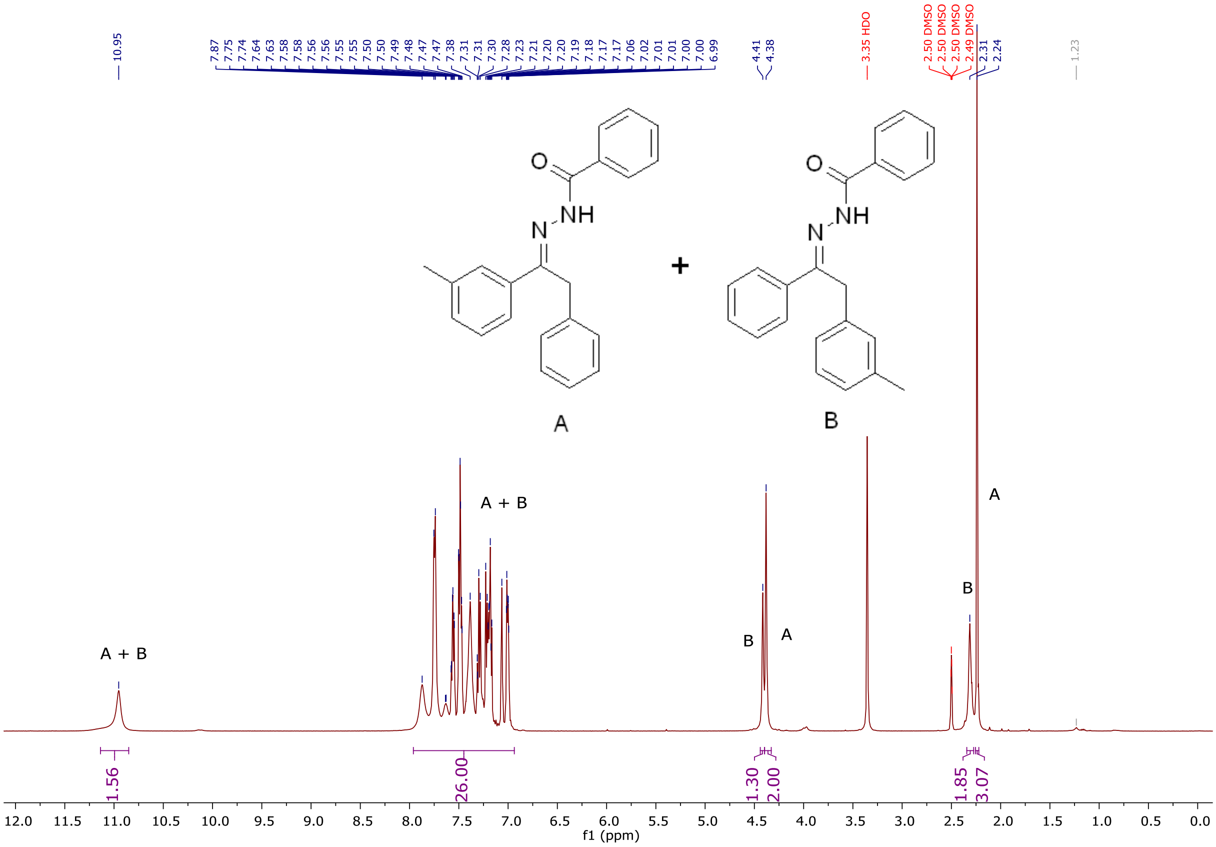

Figure S54: ^{1}H NMR of *N*'-(2-phenyl-1-(*m*-tolyl)ethylidene)benzohydrazide and *N*'-(1-phenyl-2-(*m*-tolyl)ethylidene)-benzohydrazide (7i) in DMSO-d_6.

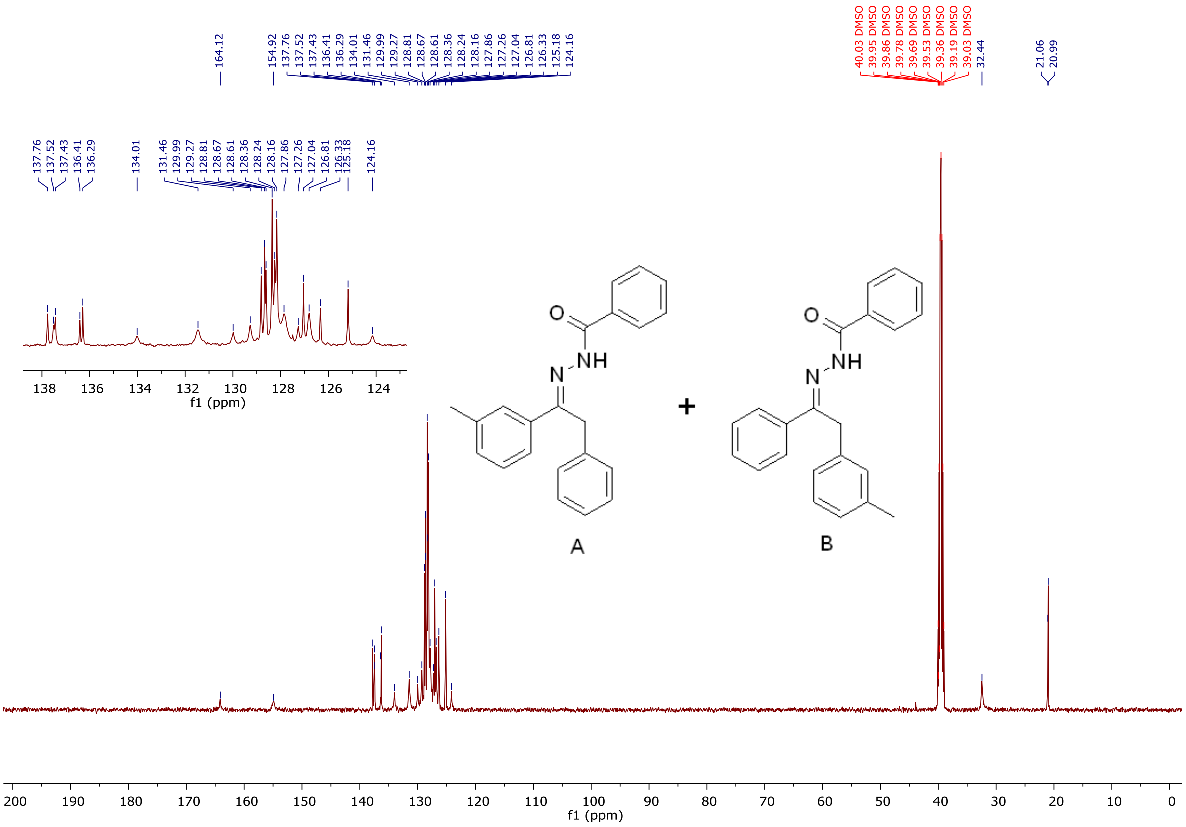

Figure S55: ^{13}C NMR of *N*'-(2-phenyl-1-(*m*-tolyl)ethylidene)benzohydrazide and *N*'-(1-phenyl-2-(*m*-tolyl)ethylidene)-benzohydrazide (7i) in DMSO-d_6.

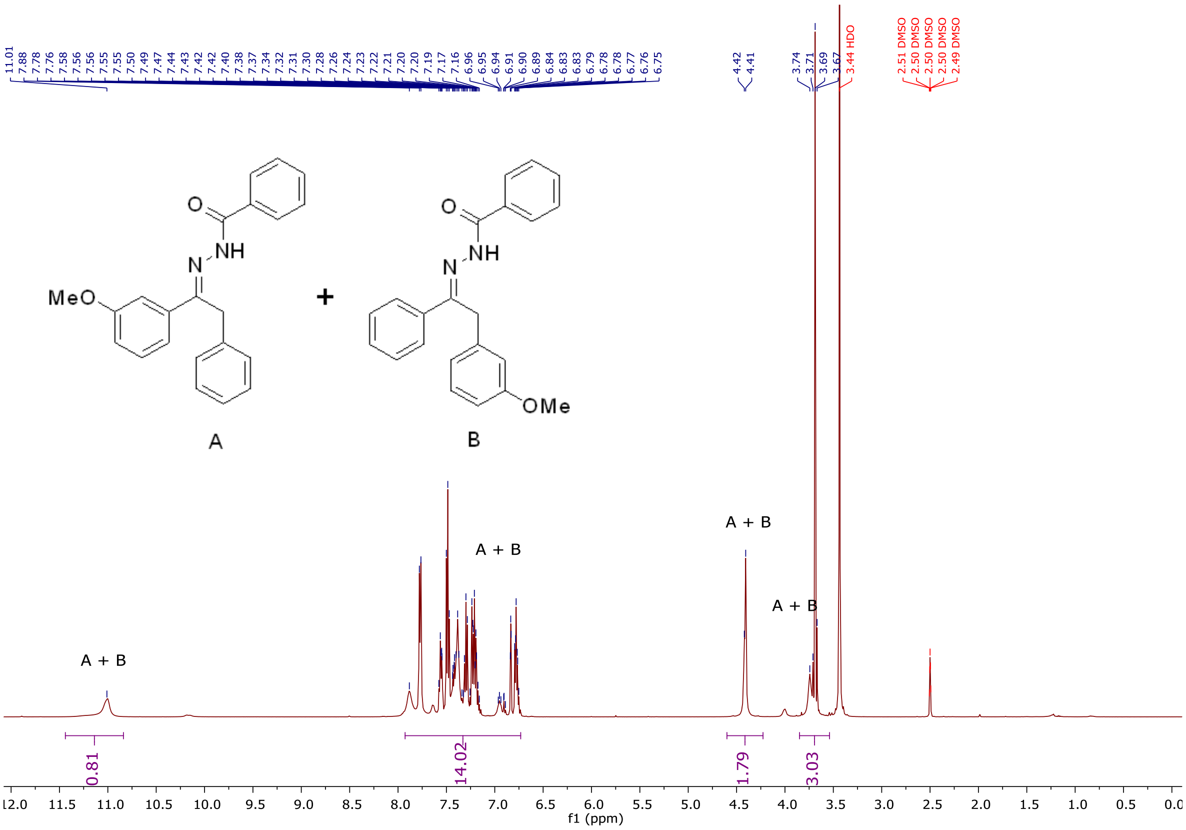

Figure S56: ^{1}H NMR of *N*'-(1-(3-methoxyphenyl)-2-phenylethylidene)benzohydrazide and *N*'-(2-(3-methoxyphenyl)-1-phenylethylidene)benzohydrazide (7j) in DMSO-d_6.

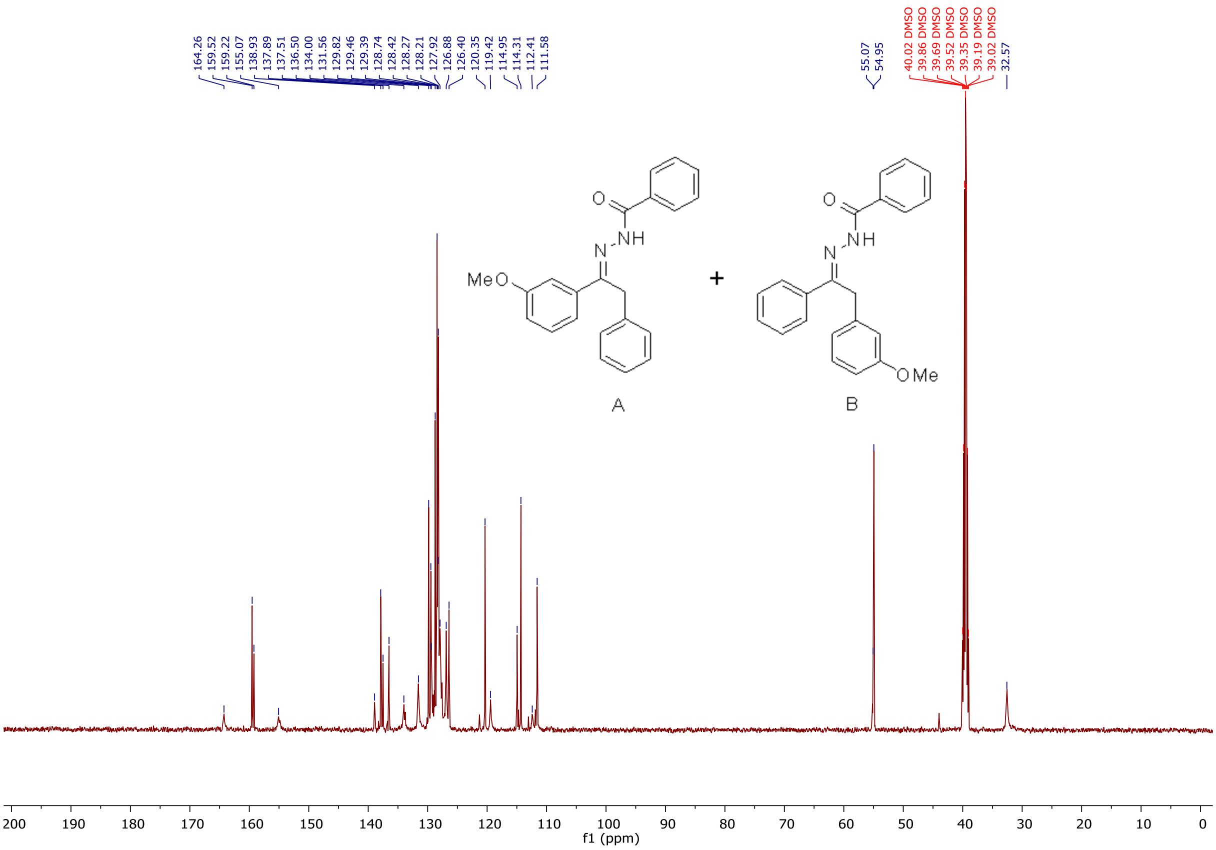

Figure S57: ^{13}C NMR of *N*'-(1-(3-methoxyphenyl)-2-phenylethylidene)benzohydrazide and *N*'-(2-(3-methoxyphenyl)-1-phenylethylidene)benzohydrazide (7j) in DMSO-d_6.

3. Mass spectra

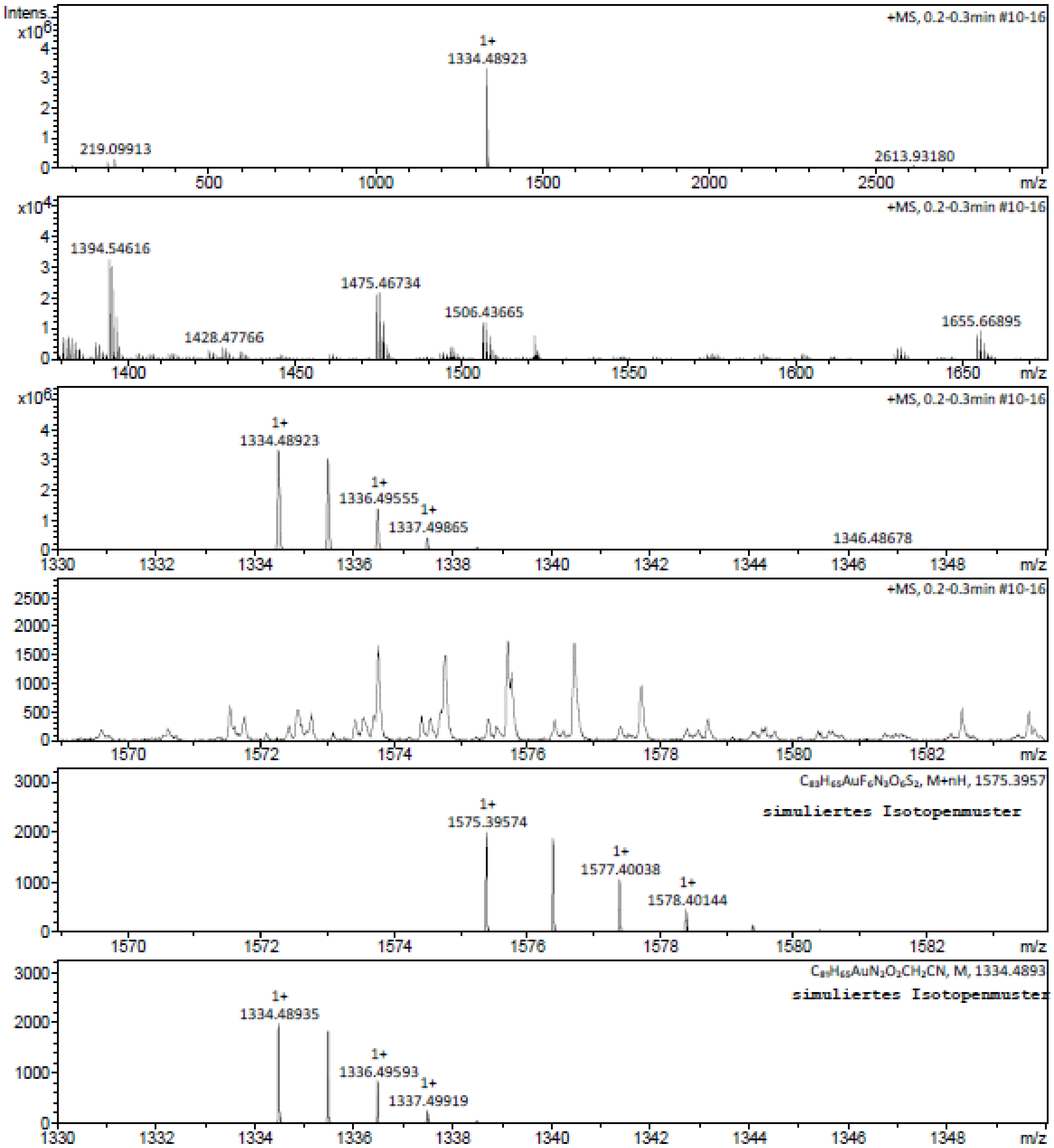

Meas. m/z	#	Ion Formula	Score	m/z	err [mDa]	err [ppm]	mSigma	rdb	e⁻ Conf	N-Rule	Adduct
1334.48923	1	C83H67AuN3O2	100.00	1334.48935	0.12	0.09	2.7	51.5	even	ok	M

Figure S58: HRMS of complex 1.

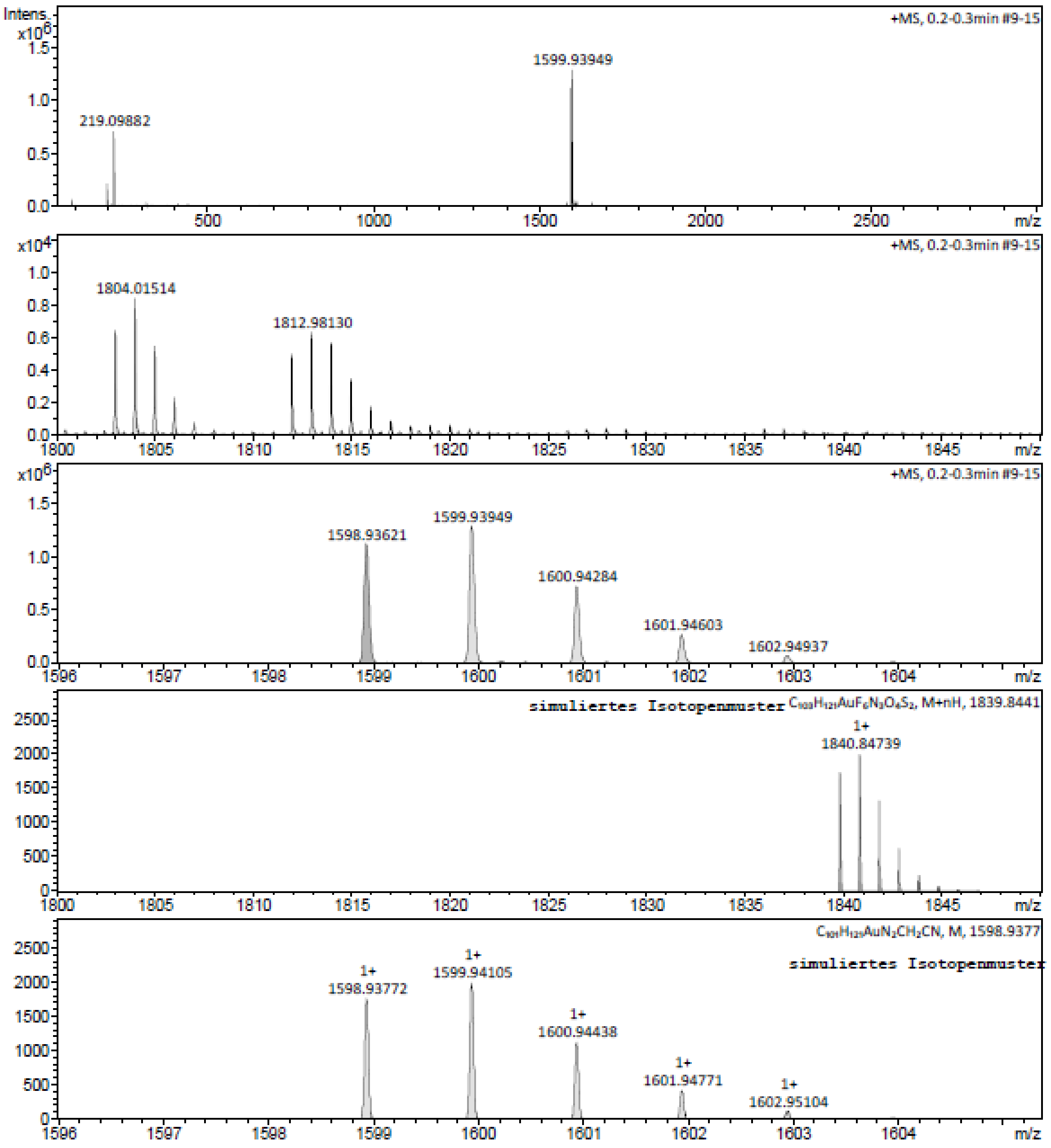

#	Meas. m/z	Ion Formula	m/z	Sum Formula	|err| [mDa]	err [ppm]	e¯ Conf	Adduct
1	1598.93621	C103H123AuN3	1598.93772	C103H123AuN3	1.51	0.95	even	M
2	1598.93621	C100H125AuN2O3	1598.93504	C100H125AuN2O3	1.17	-0.73	odd	M
3	1598.93621	C89H254AuNO4	1598.93631	C89H254AuNO4	0.10	0.06	odd	M
4	1598.93621	C87H252AuN4O3	1598.93497	C87H252AuN4O3	1.24	-0.78	even	M

Figure S59: HRMS of complex 2.

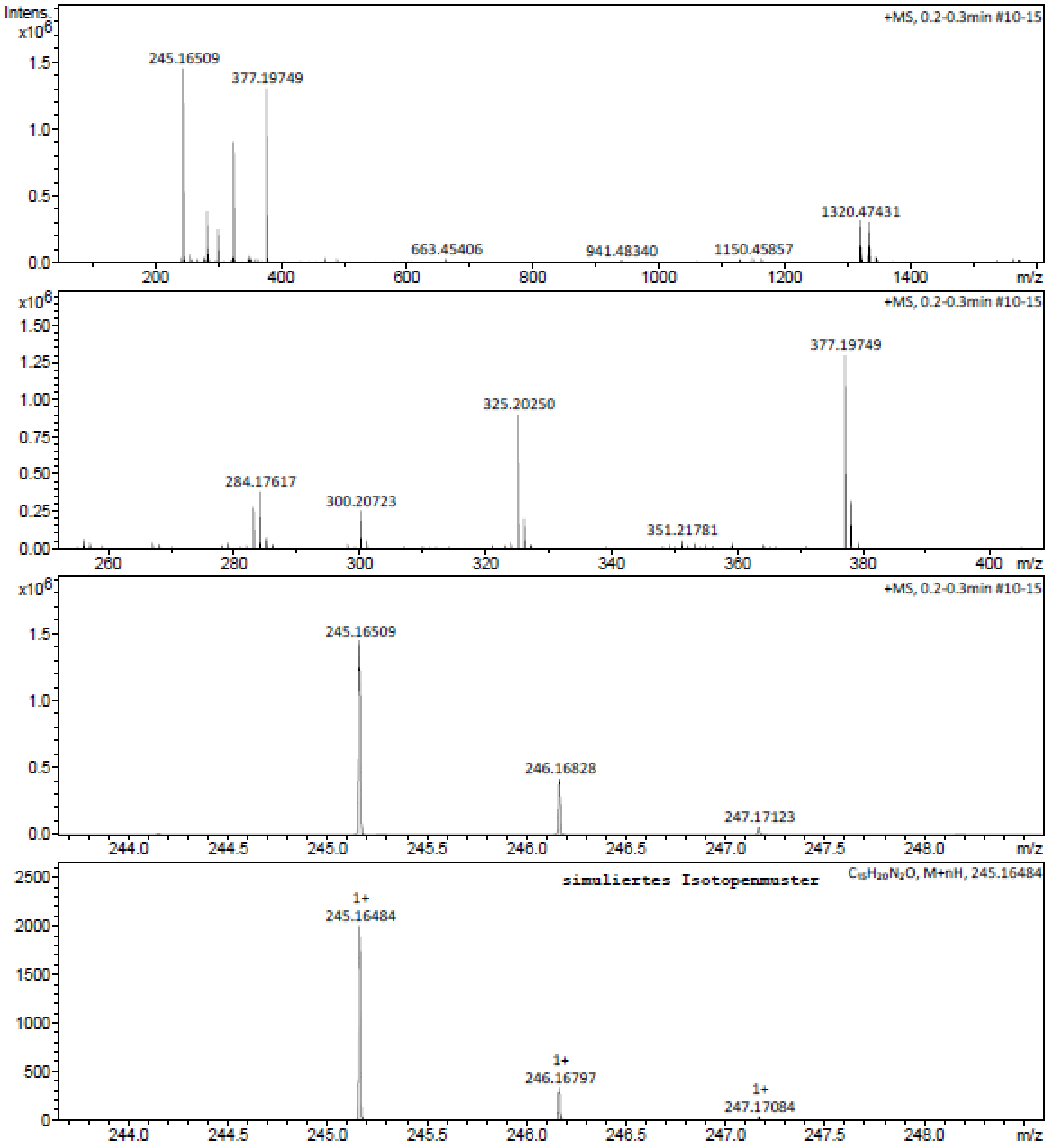

#	Meas. m/z	Ion Formula	m/z	Sum Formula	|err| [mDa]	err [ppm]	e⁻ Conf	Adduct
1	245.16509	C15H21N2O	245.16484	C15H20N2O	0.25	-1.04	even	M+H

Figure S60: HRMS of *N*'-(1-cyclohexylethylidene)benzohydrazide (6b).

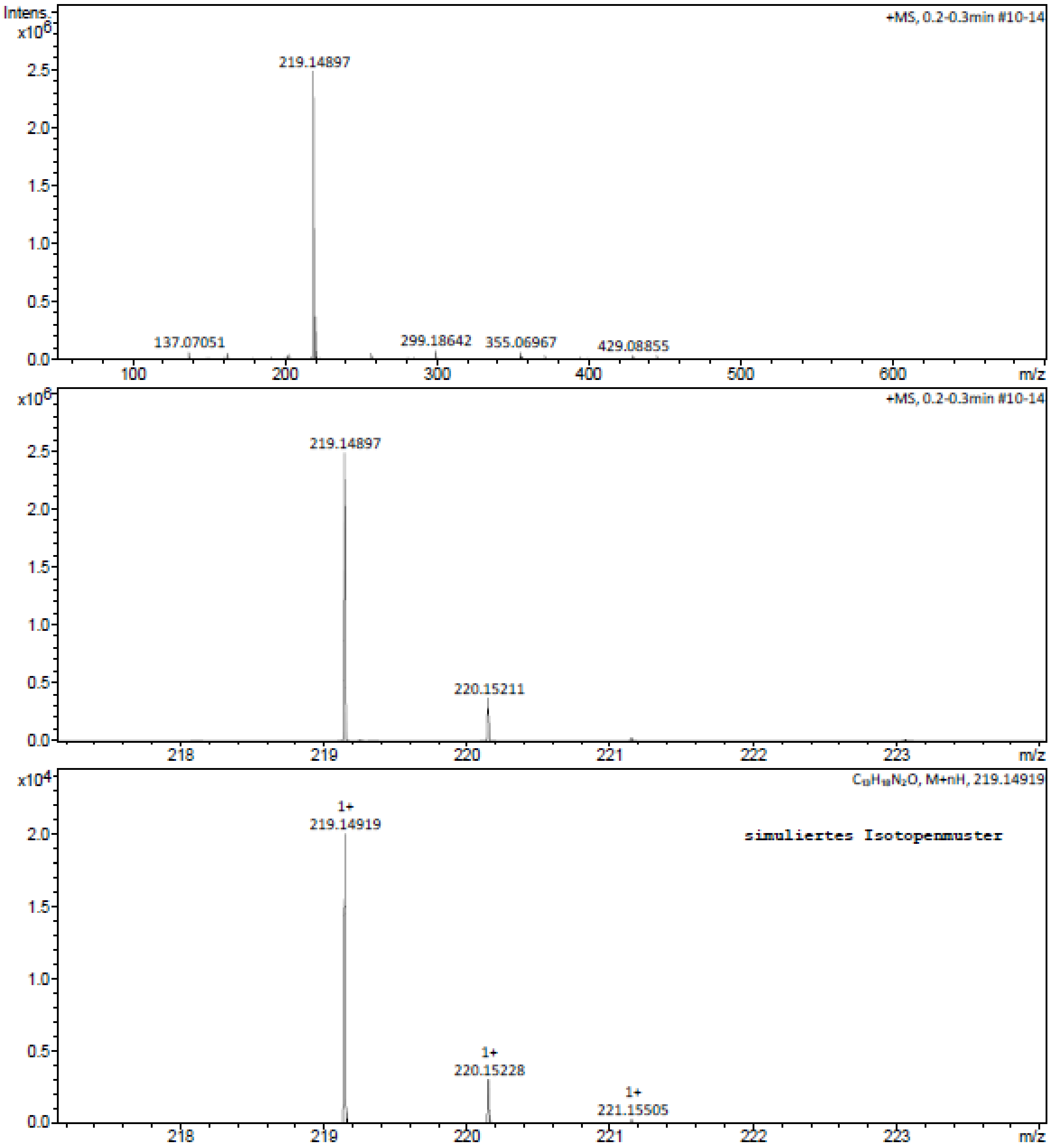

#	Meas. m/z	Ion Formula	m/z	Sum Formula	|err| [mDa]	err [ppm]	e⁻ Conf	Adduct
1	219.14897	C13H19N2O	219.14919	C13H18N2O	0.22	1.02	even	M+H

Figure S61: HRMS of *N*'-(3,3-dimethylbutan-2-ylidene)benzohydrazide (6c).

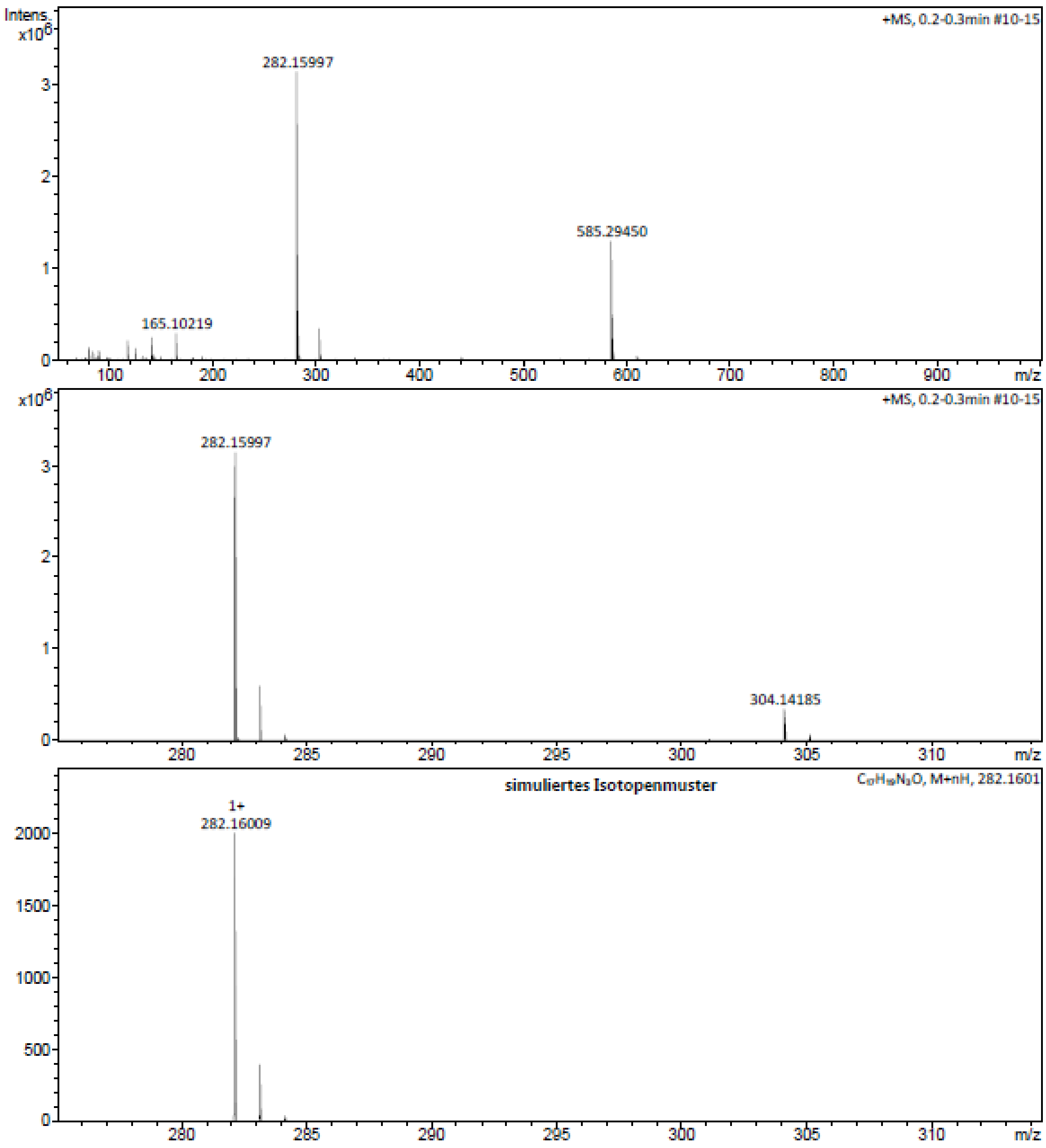

#	Meas. m/z	Ion Formula	m/z	Sum Formula	|err| [mDa]	err [ppm]	e⁻ Conf	Adduct
1	282.15997	C17H20N3O	282.16009	C17H19N3O	0.11	0.41	even	M+H
1	304.14185	C17H19N3NaO	304.14203	C17H19N3O	0.18	0.60	even	M+Na
1	585.29450	C34H38N6NaO2	585.29485	C17H19N3O	0.34	0.59	even	2M+Na

Figure S62: HRMS of 4-(dimethylamino)-(1-phenylethylidene)benzohydrazide (6e).

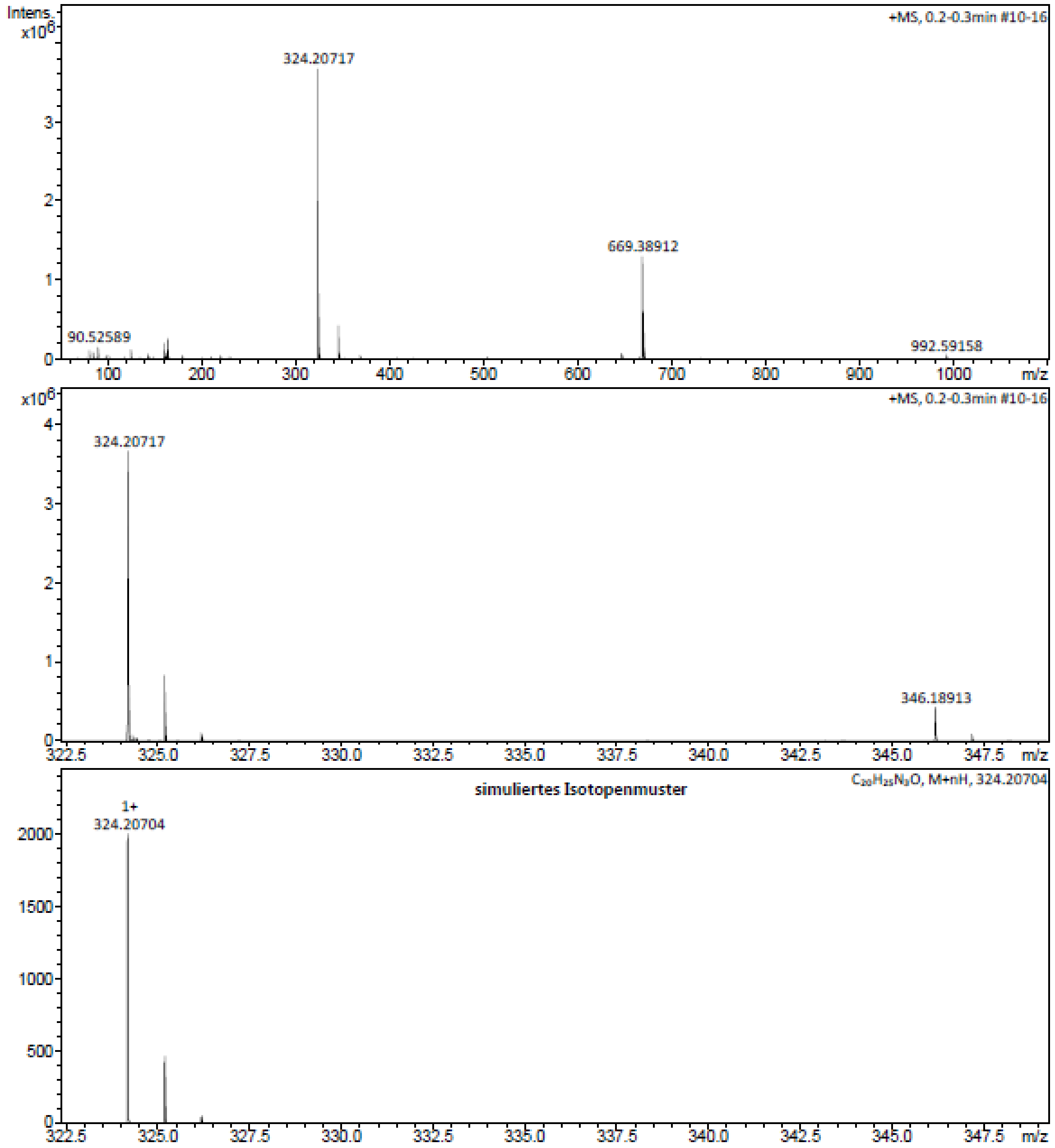

#	Meas. m/z	Ion Formula	m/z	Sum Formula	|err| [mDa]	err [ppm]	e⁻ Conf	Adduct
1	324.20717	C20H26N3O	324.20704	C20H25N3O	0.13	-0.41	even	M+H
1	669.38912	C40H50N6NaO2	669.38875	C20H25N3O	0.37	-0.56	even	2M+Na

Figure S63: HRMS of 4-(dimethylamino)-*N*'-(1-mesitylethylidene)benzohydrazide (6h).

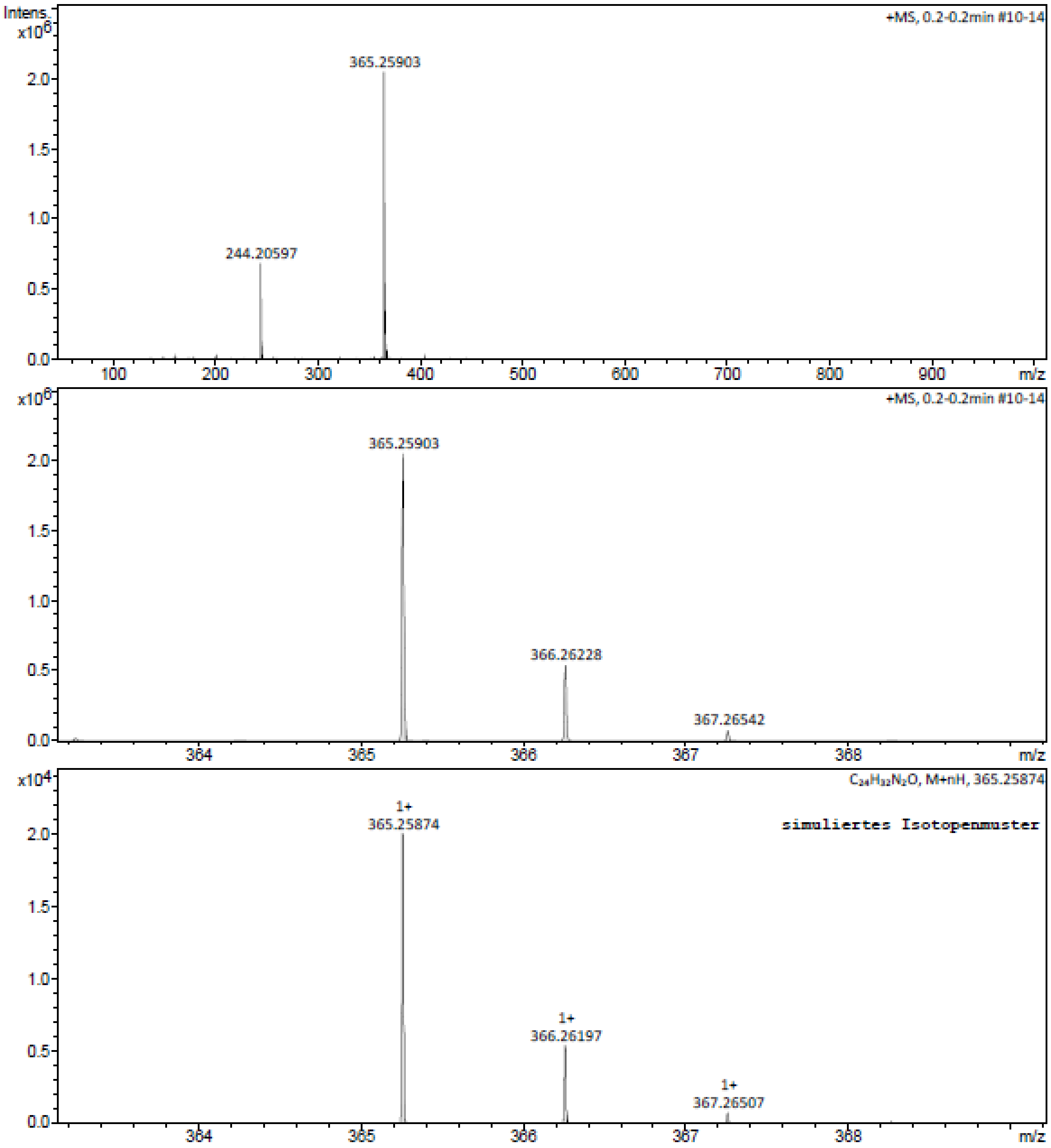

#	Meas. m/z	Ion Formula	m/z	Sum Formula	|err| [mDa]	err [ppm]	e⁻ Conf	Adduct
1	365.25903	C24H33N2O	365.25874	C24H32N2O	0.29	-0.81	even	M+H

Figure S64: HRMS of *N*'-(1-(2,4,6-triisopropylphenyl)ethylidene)benzohydrazide (6i).

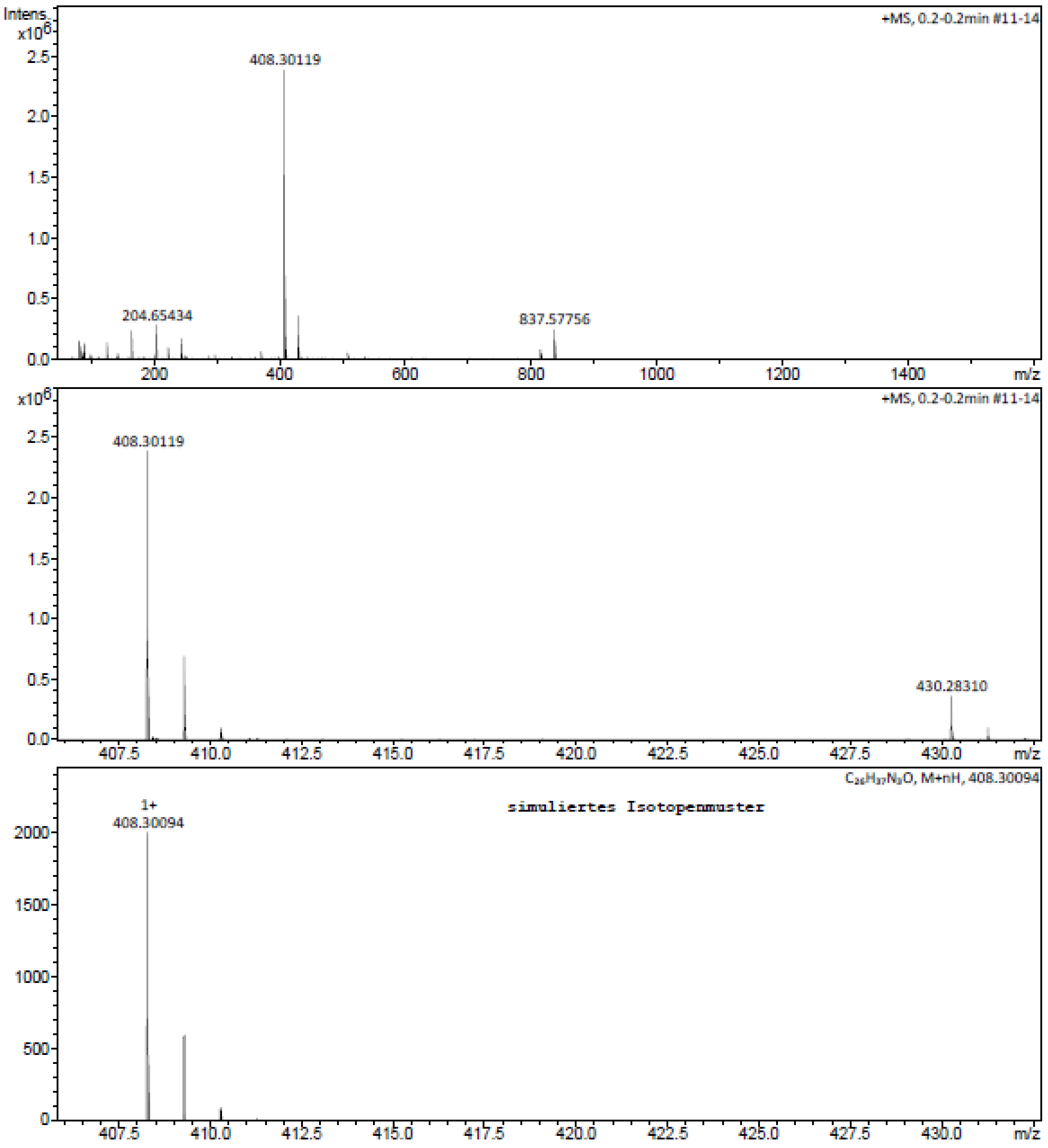

#	Meas. m/z	Ion Formula	m/z	Sum Formula	|err| [mDa]	err [ppm]	e⁻ Conf	Adduct
1	408.30119	C26H38N3O	408.30094	C26H37N3O	0.25	-0.61	even	M+H
1	430.28310	C26H37N3NaO	430.28288	C26H37N3O	0.21	-0.49	even	M+Na
1	815.59513	C52H75N6O2	815.59460	C26H37N3O	0.53	-0.65	even	2M+H
1	837.57756	C52H74N6NaO2	837.57655	C26H37N3O	1.01	-1.21	even	2M+Na

Figure S65: HRMS of 4-(dimethylamino)-*N*'-(1-(2,4,6-triisopropylphenyl)ethylidene)benzohydrazide (6j).

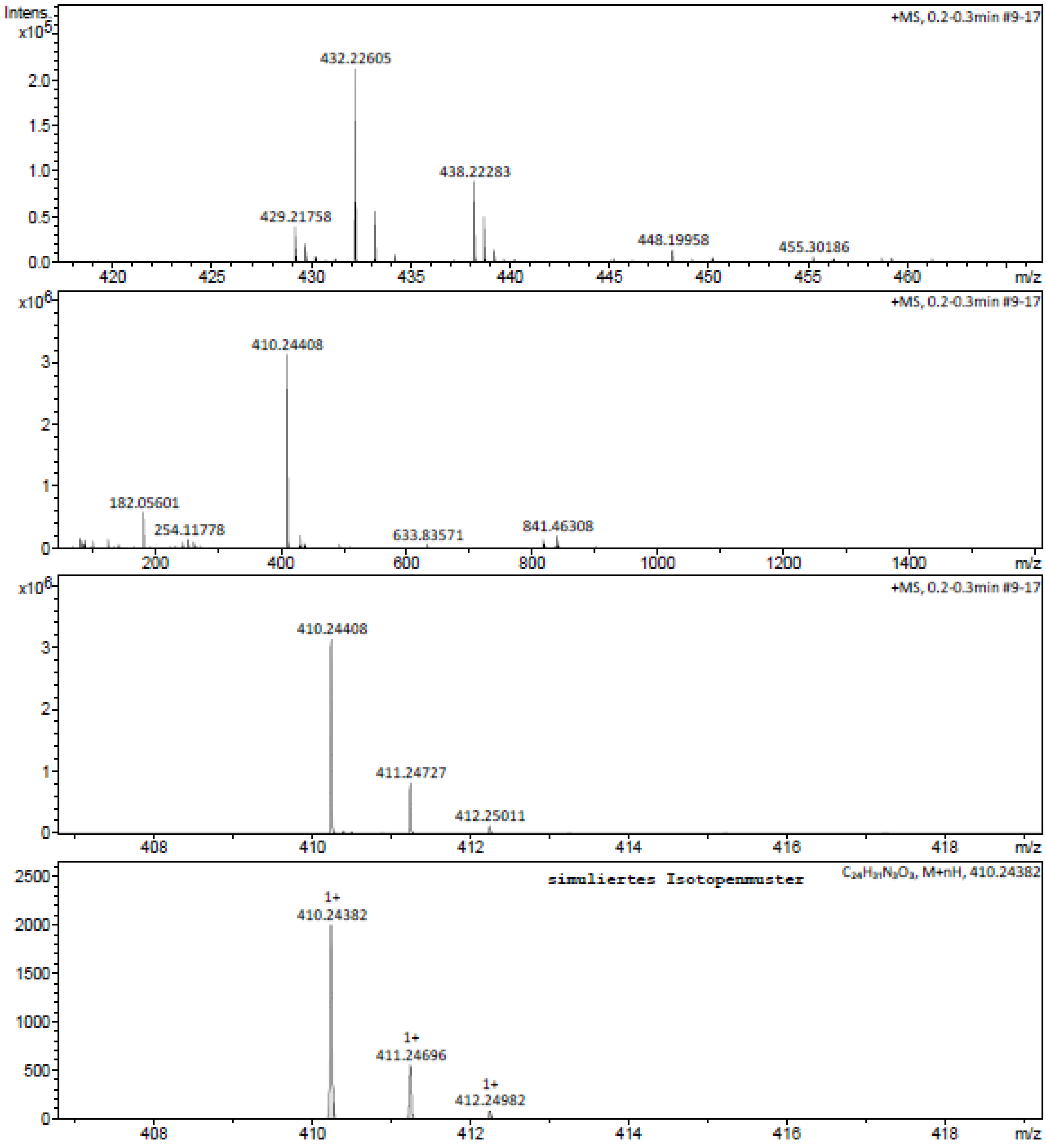

#	Meas. m/z	Ion Formula	m/z	Sum Formula	|err| [mDa]	err [ppm]	e⁻ Conf	Adduct
1	410.24408	C24H32N3O3	410.24382	C24H31N3O3	0.27	-0.65	even	M+H
1	432.22605	C24H31N3NaO3	432.22576	C24H31N3O3	0.29	-0.67	even	M+Na
1	819.48091	C48H63N6O6	819.48036	C24H31N3O3	0.55	-0.67	even	2M+H
1	841.46308	C48H62N6NaO6	841.46230	C24H31N3O3	0.78	-0.93	even	2M+Na

Figure S66: HRMS of 4-nitro-*N*'-(1-(2,4,6-triisopropylphenyl)ethylidene)benzohydrazide (6k).

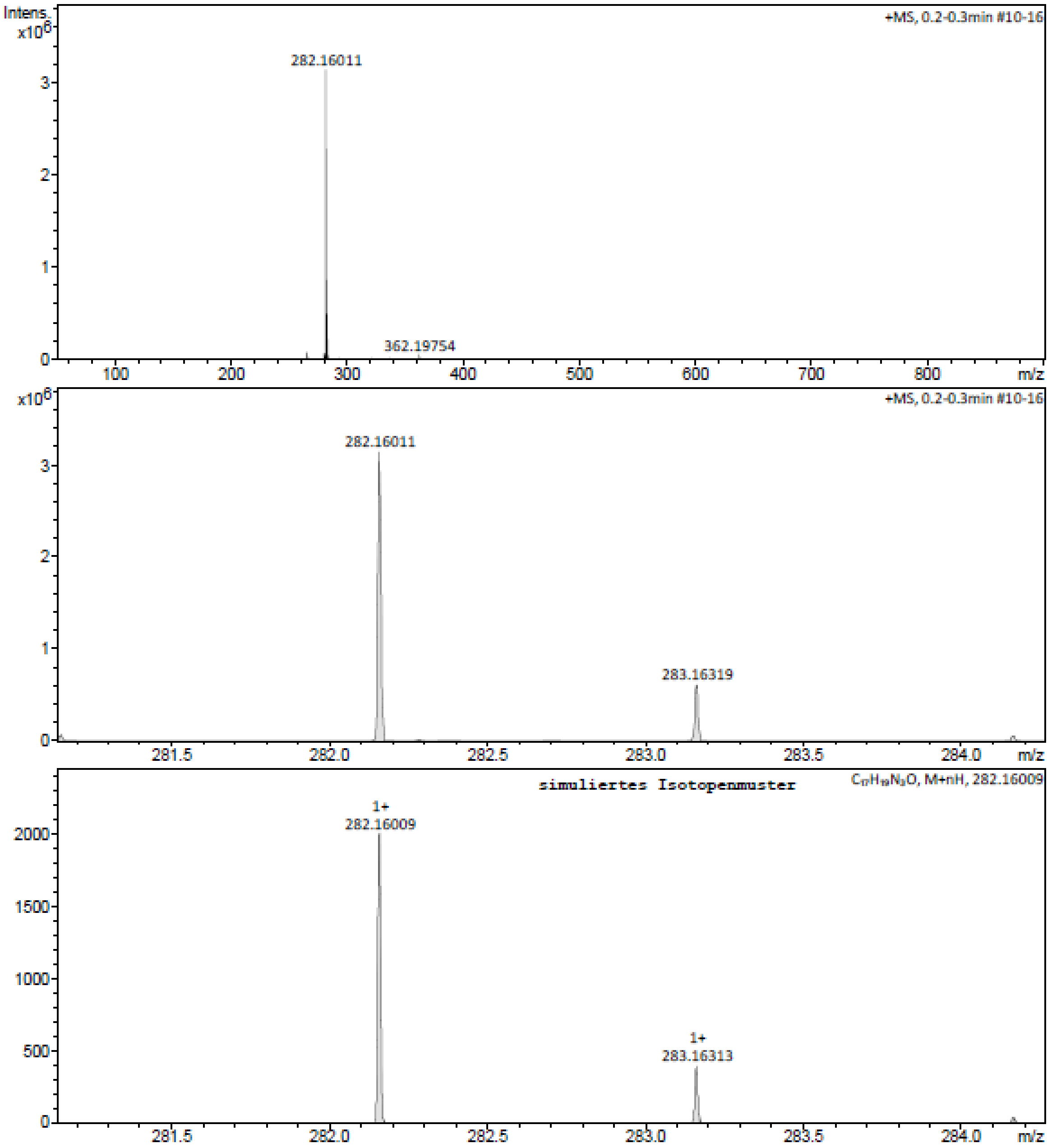

#	Meas. m/z	Ion Formula	m/z	Sum Formula	|err| [mDa]	err [ppm]	e⁻ Conf	Adduct
1	282.16011	C17H20N3O	282.16009	C17H19N3O	0.02	-0.07	even	M+H

Figure S67: HRMS of *N*'-(1-(4-(dimethylamino)phenyl)ethylidene)benzohydrazide (6l).

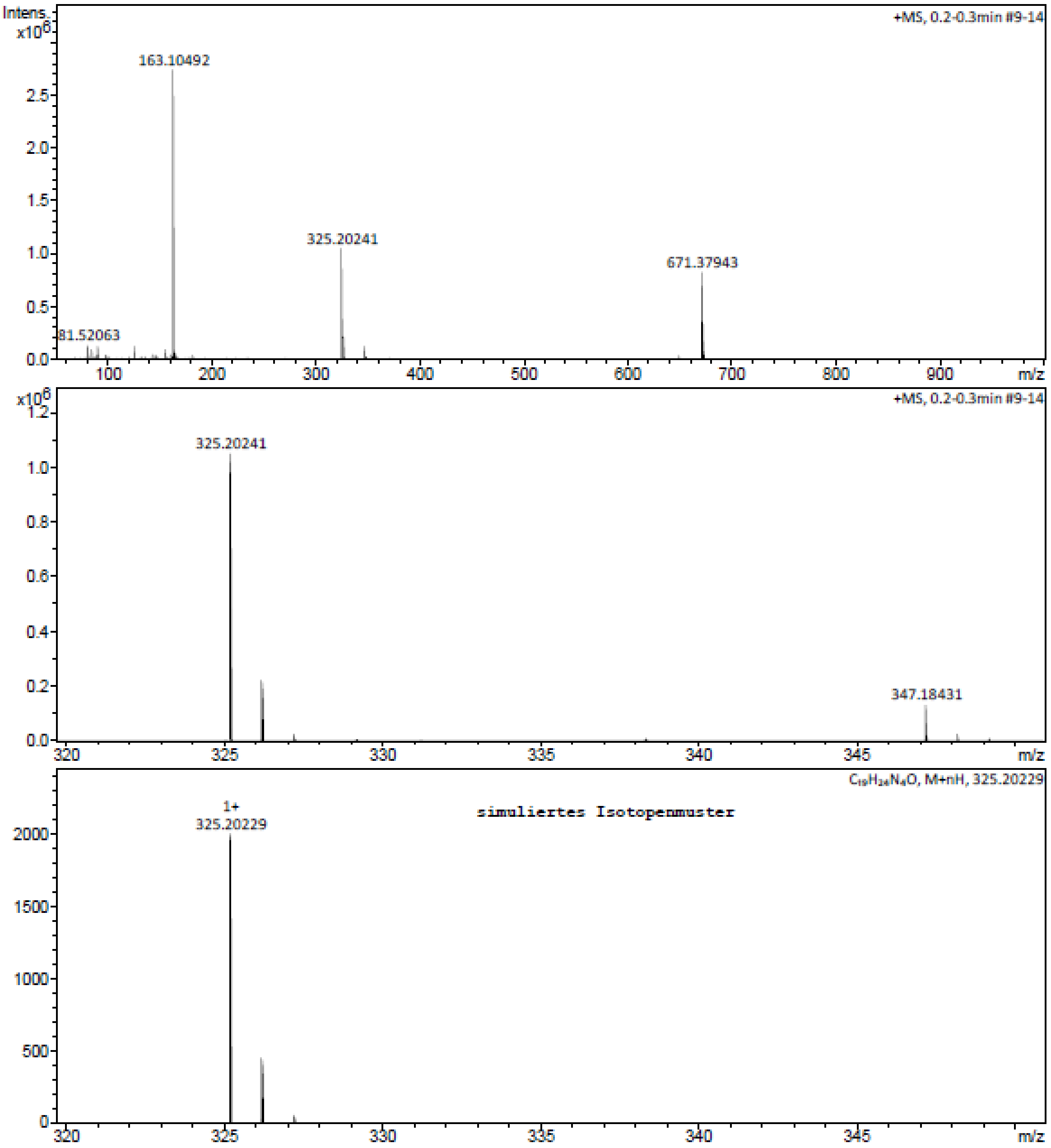

#	Meas. m/z	Ion Formula	m/z	Sum Formula	|err| [mDa]	err [ppm]	e⁻ Conf	Adduct
1	325.20241	C19H25N4O	325.20229	C19H24N4O	0.12	-0.37	even	M+H
1	671.37943	C38H48N8NaO2	671.37924	C19H24N4O	0.19	-0.28	even	2M+Na

Figure S68: HRMS of 4-(dimethylamino)-*N*'-(1-(4-(dimethylamino)phenyl)ethylidene)benzohydrazide (6m).

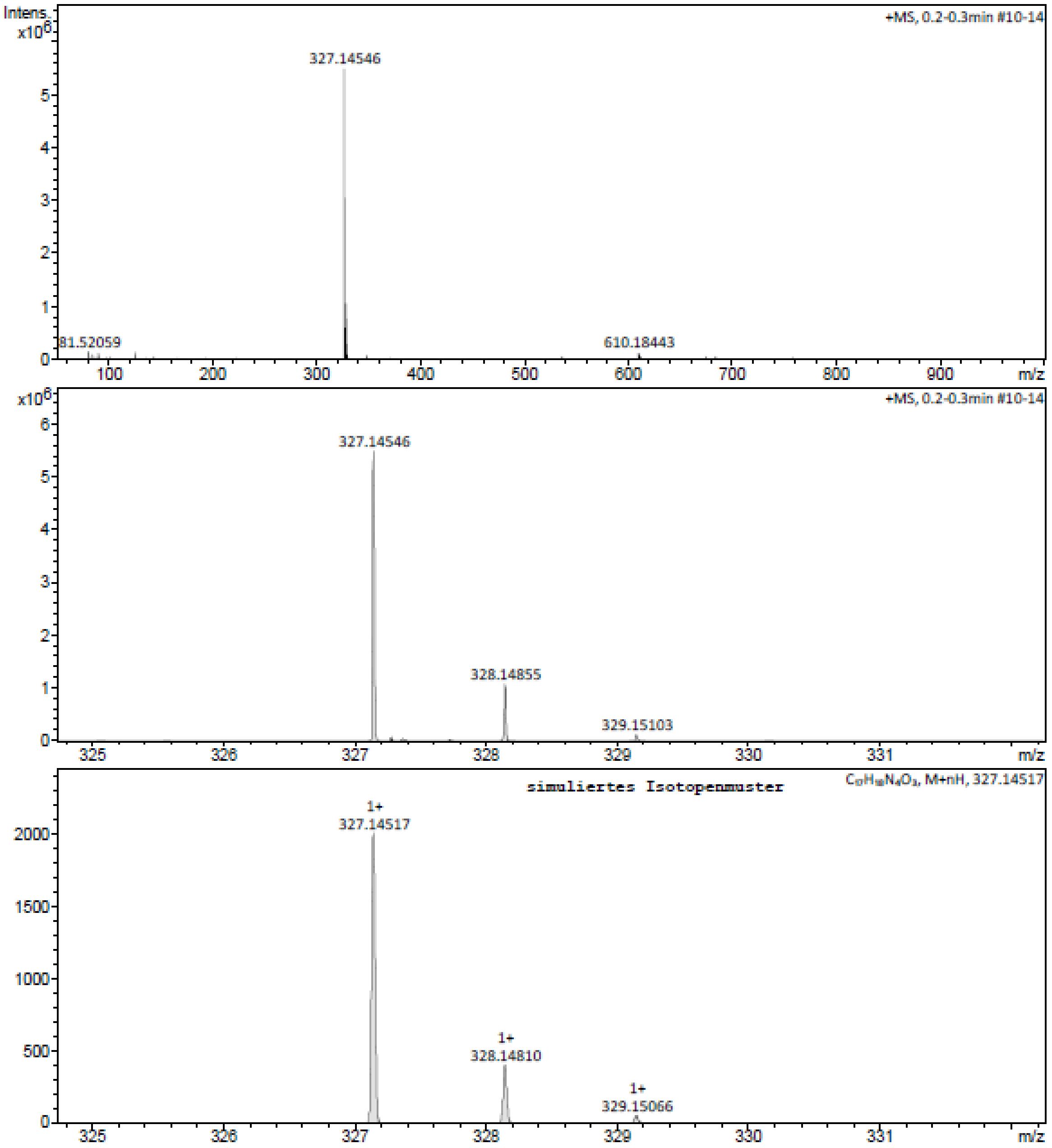

#	Meas. m/z	Ion Formula	m/z	Sum Formula	|err| [mDa]	err [ppm]	e⁻ Conf	Adduct
1	327.14546	C17H19N4O3	327.14517	C17H18N4O3	0.29	-0.90	even	M+H

Figure S69: HRMS of 4-nitro-*N*'-(1-(4-(dimethylamino)phenyl)ethylidene)benzohydrazide (6n).

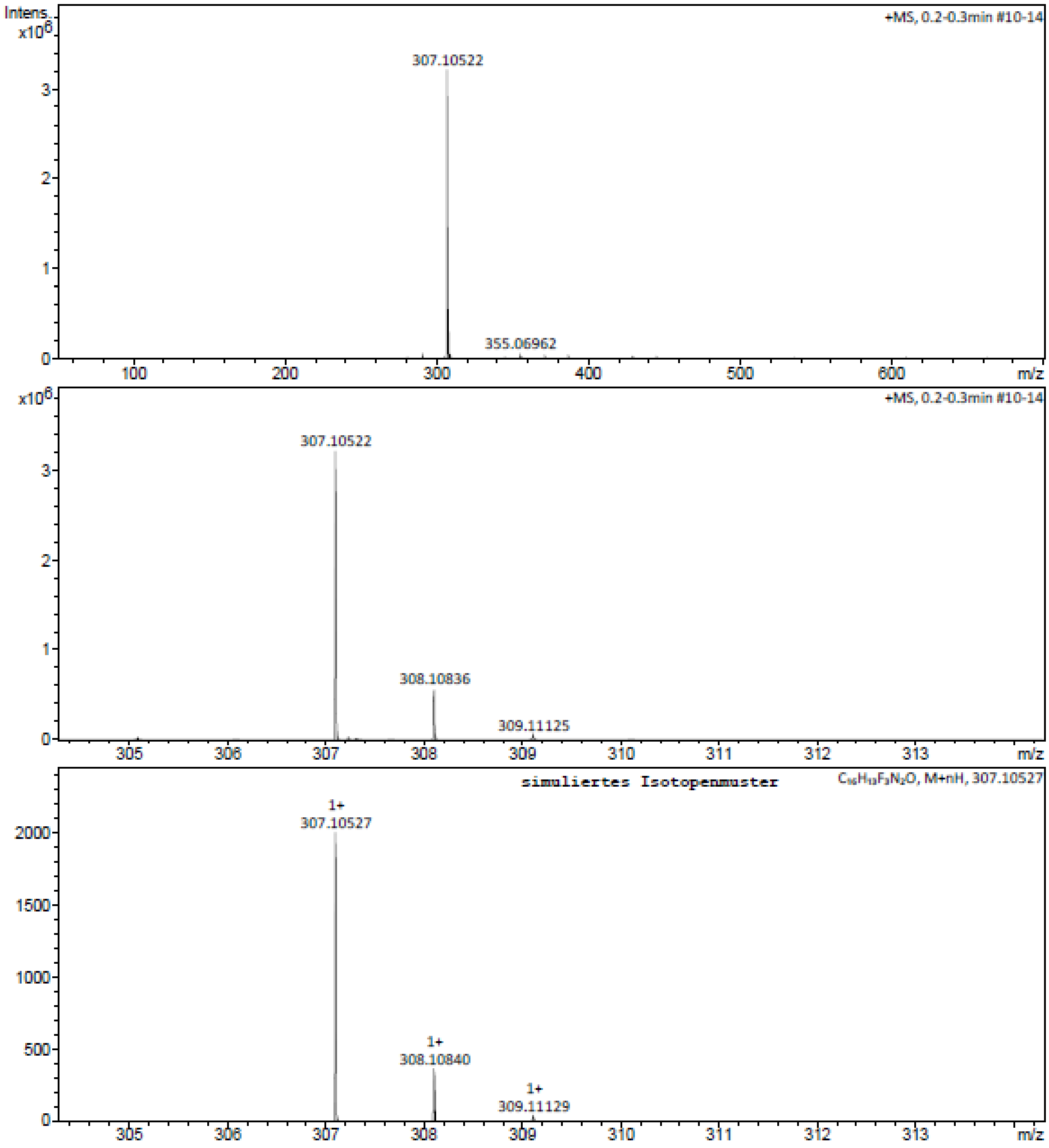

#	Meas. m/z	Ion Formula	m/z	Sum Formula	|err| [mDa]	err [ppm]	e¯ Conf	Adduct
1	307.10522	C16H14F3N2O	307.10527	C16H13F3N2O	0.06	0.18	even	M+H

Figure S70: HRMS of *N*'-(1-(4-(trifluoromethyl)phenyl)ethylidene)benzohydrazide (6p).

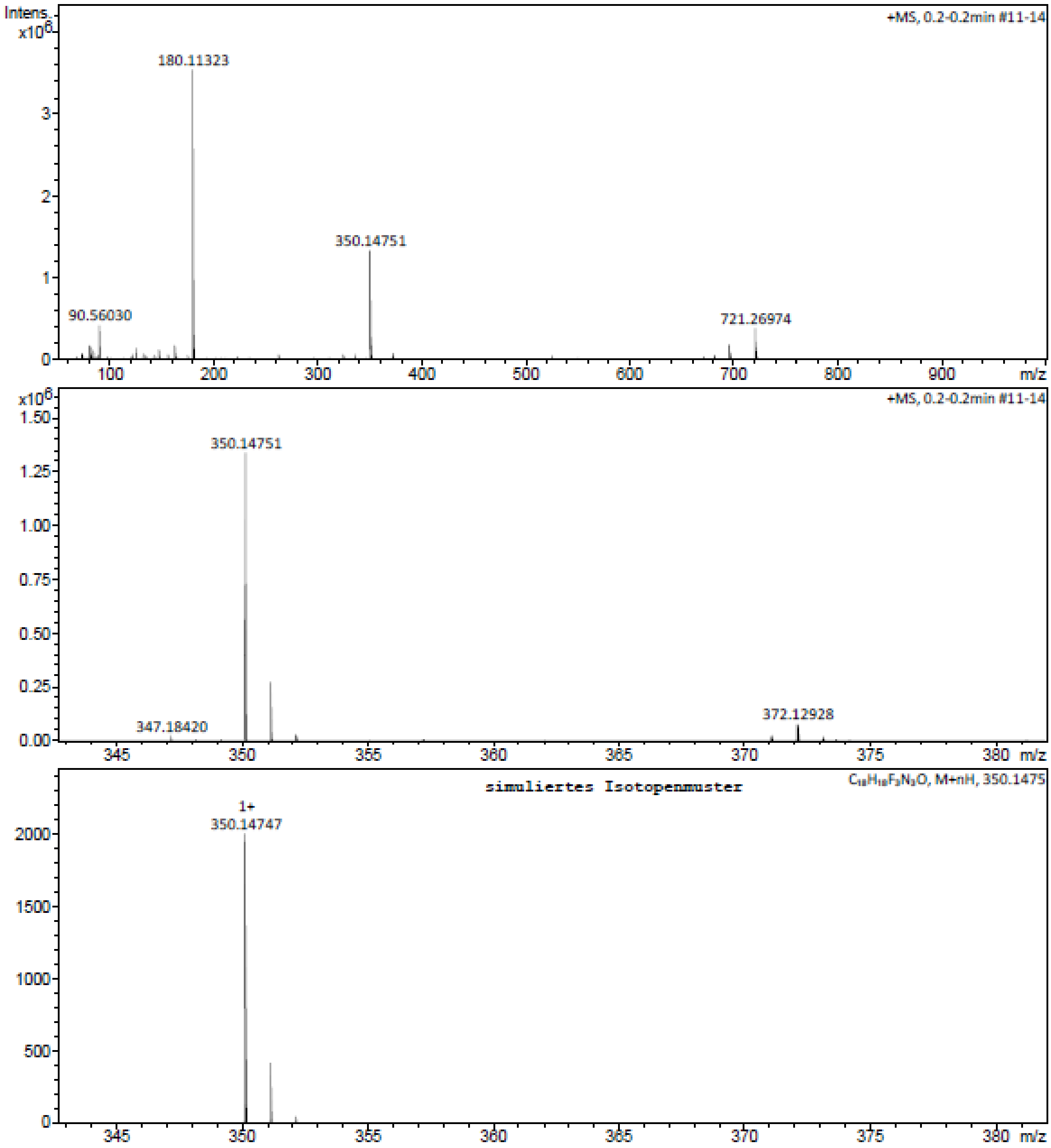

#	Meas. m/z	Ion Formula	m/z	Sum Formula	|err| [mDa]	err [ppm]	e⁻ Conf	Adduct
1	350.14751	C18H19F3N3O	350.14747	C18H18F3N3O	0.04	-0.12	even	M+H
1	721.26974	C36H36F6N6NaO2	721.26961	C18H18F3N3O	0.13	-0.17	even	2M+Na

Figure S71: HRMS of 4-(dimethylamino)-*N*'-(1-(4-(trifluoromethyl)phenyl)ethylidene)benzohydrazide (6q).

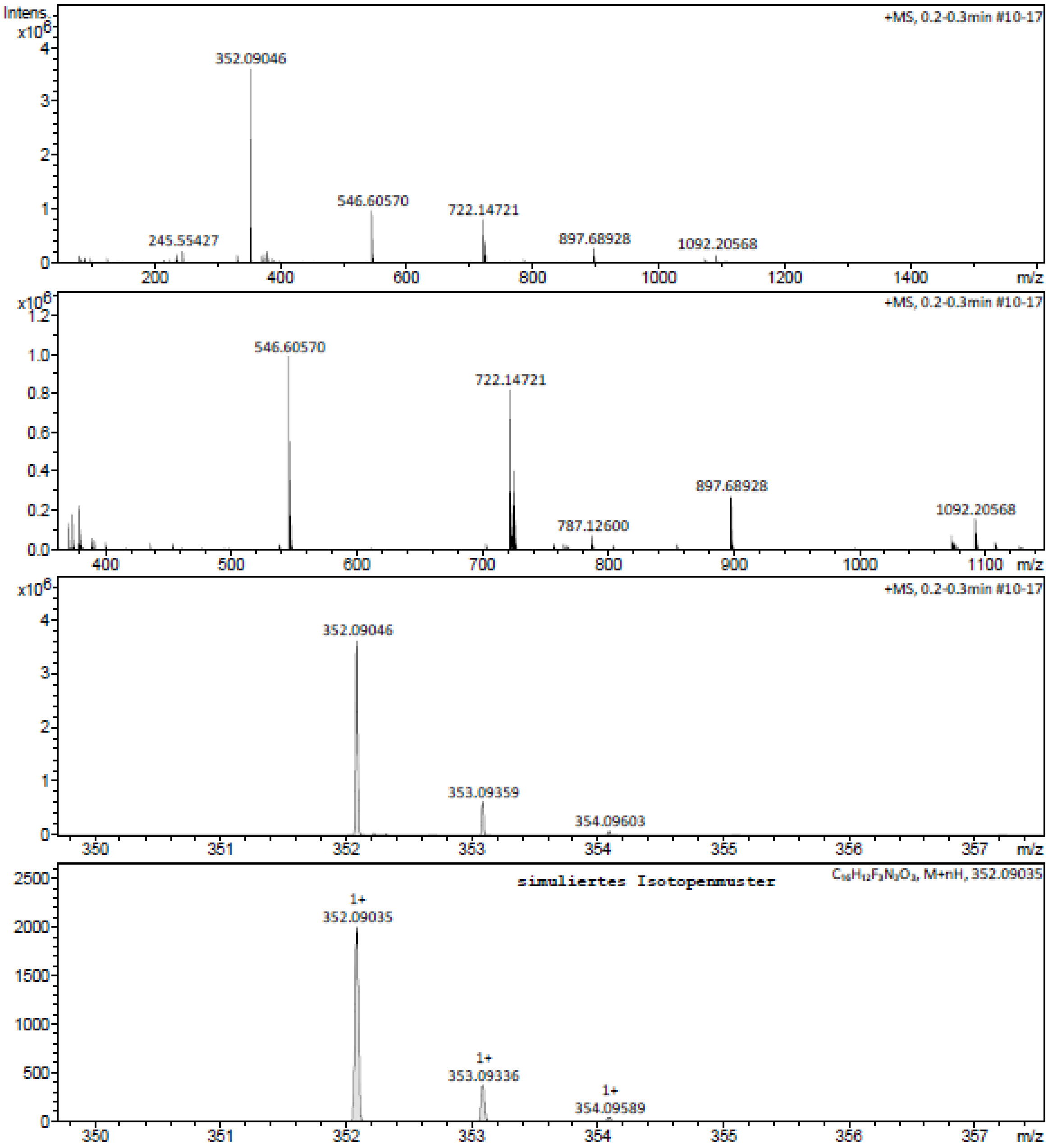

#	Meas. m/z	Ion Formula	m/z	Sum Formula	|err| [mDa]	err [ppm]	e⁻ Conf	Adduct
1	352.09046	C16H13F3N3O3	352.09035	C16H12F3N3O3	0.10	-0.29	even	M+H

Figure S72: HRMS of 4-nitro-*N*'-(1-(4-(trifluoromethyl)phenyl)ethylidene)benzohydrazide (6r).

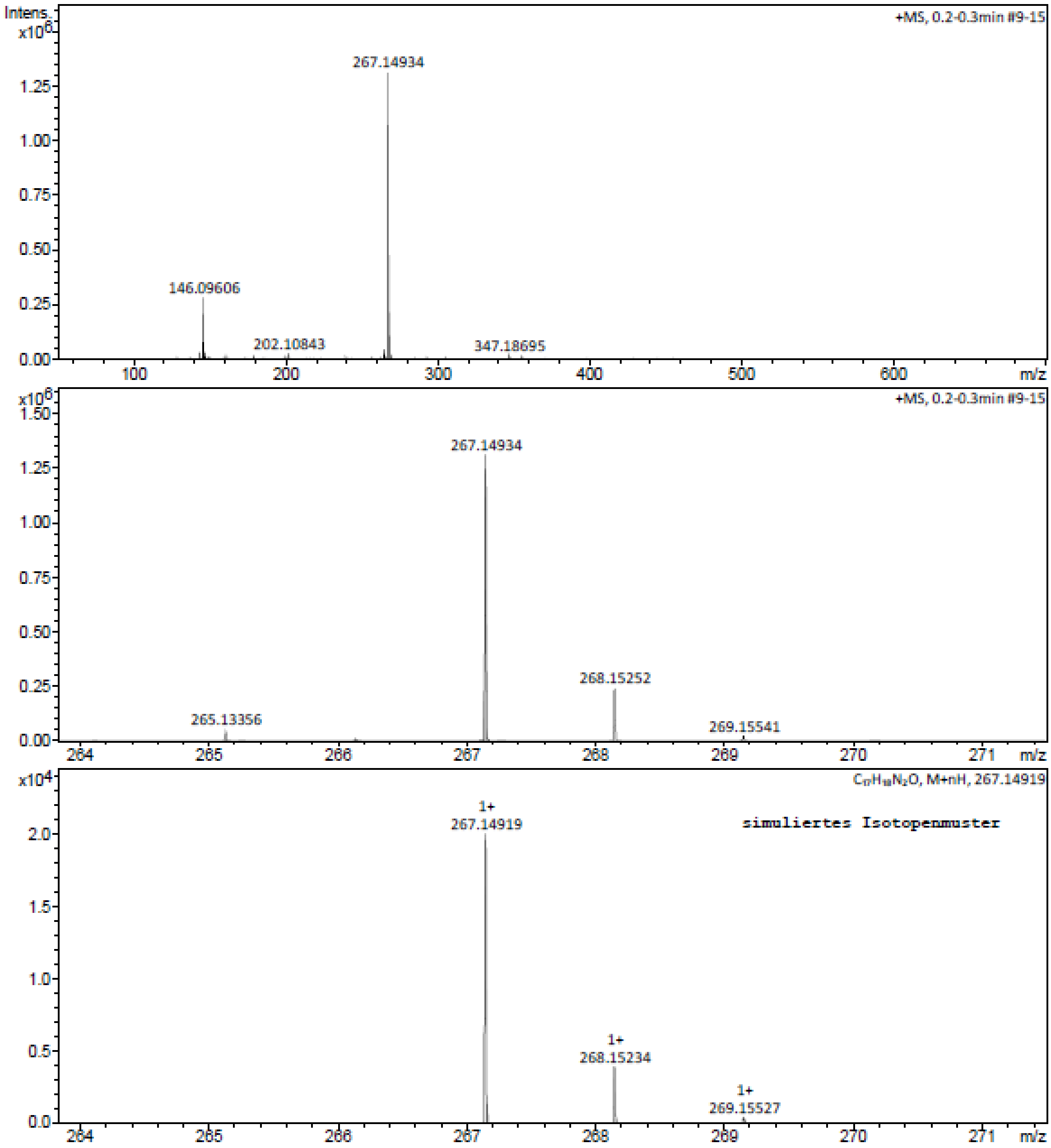

#	Meas. m/z	Ion Formula	m/z	Sum Formula	|err| [mDa]	err [ppm]	e⁻ Conf	Adduct
1	267.14934	C17H19N2O	267.14919	C17H18N2O	0.15	-0.55	even	M+H

Figure S73: HRMS of *N*'-(1-phenylbutylidene)benzohydrazide and *N*'-(1-phenylbutan-2-ylidene)benzohydrazide (7b).

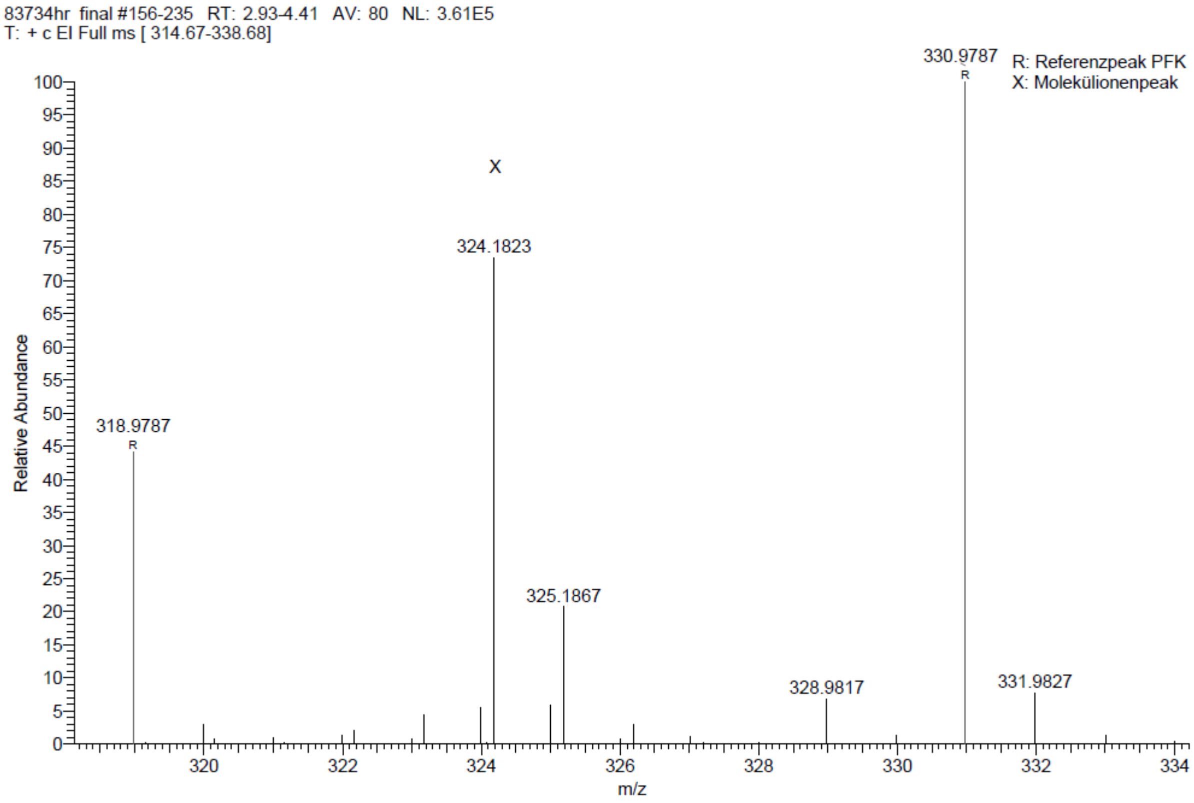

m/z= 319.1823-329.1823

m/z	Theo. Mass	Delta (mmu)	RDB equiv.	Composition
324.1823	324.1832	-0.95	10.0	$C_{20}H_{24}O_2N_2$
	324.1792	3.07	6.0	$C_{15}H_{24}O_4N_4$
	324.1873	-4.97	14.0	$C_{25}H_{24}$

Figure S74: HRMS of *N*'-(1-(3-methoxyphenyl)hexylidene)benzohydrazide and *N*'-(1-(3-methoxyphenyl)hexan-2-ylidene)benzohydrazide (7c).

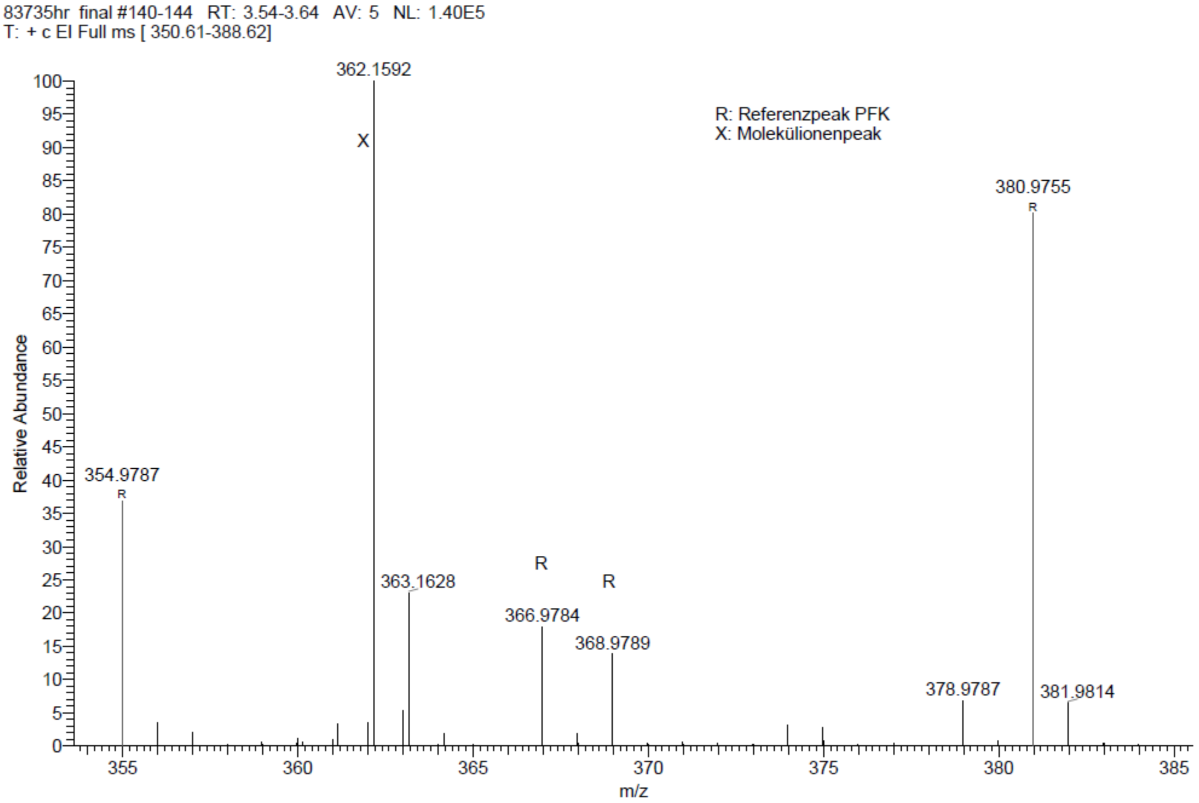

m/z= 357.1592-367.1592

m/z	Theo. Mass	Delta (mmu)	RDB equiv.	Composition
362.1592	362.1600	-0.85	10.0	$C_{20}H_{21}O_1N_2F_3$
	362.1562	2.97	9.5	$C_{20}H_{22}O_3N_1F_2$
	362.1560	3.17	6.0	$C_{15}H_{21}O_3N_4F_3$
	362.1549	4.32	10.0	$C_{18}H_{20}O_2N_4F_2$

Figure S75: HRMS of *N'*-(1-(3-(trifluoromethyl)phenyl)hexylidene)benzohydrazide and *N'*-(1-(3-(trifluoromethyl)phenyl)hexan-2-ylidene)benzohydrazide (7d).

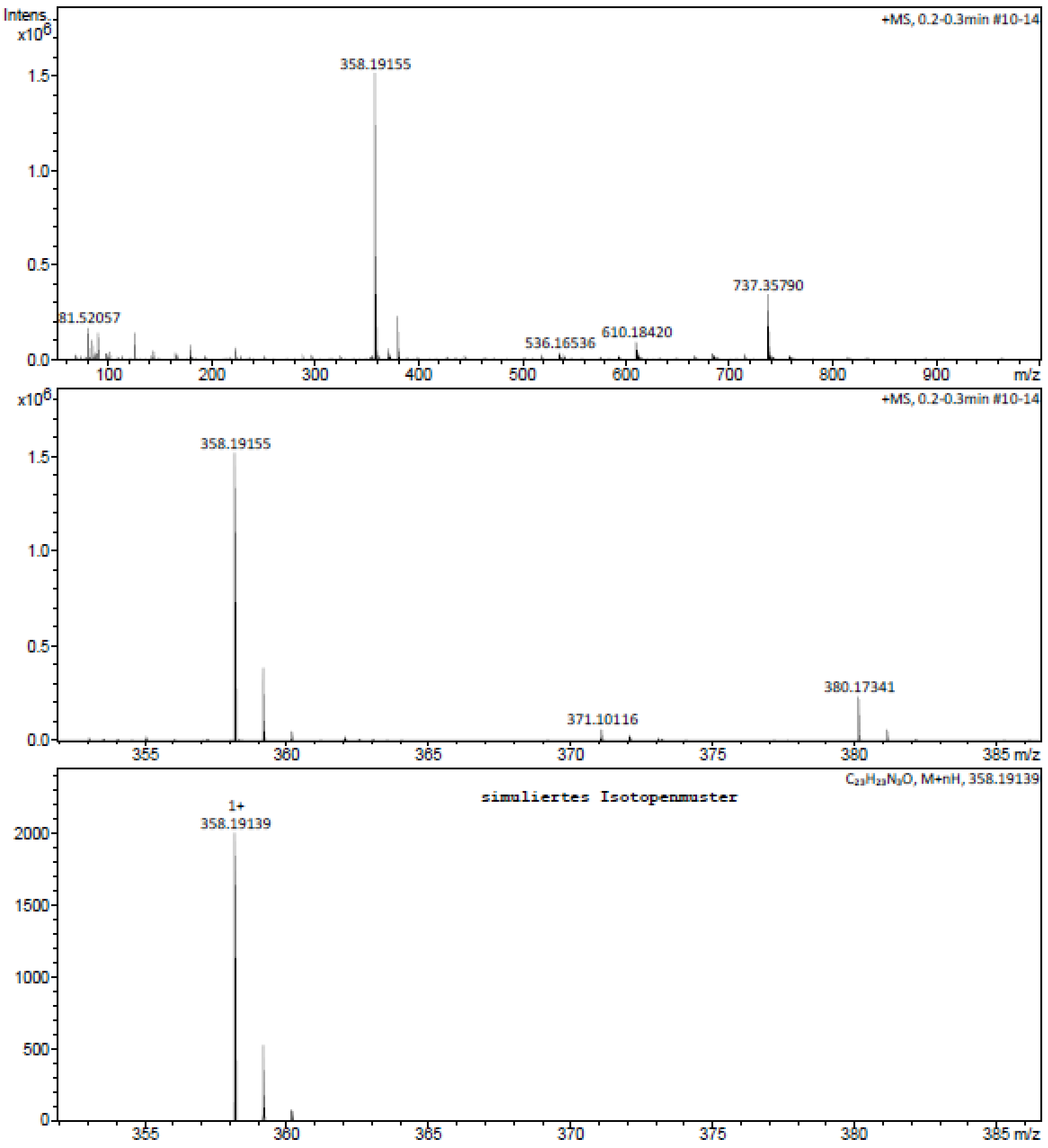

#	Meas. m/z	Ion Formula	m/z	Sum Formula	|err| [mDa]	err [ppm]	e⁻ Conf	Adduct
1	358.19155	C23H24N3O	358.19139	C23H23N3O	0.16	-0.44	even	M+H
1	737.35790	C46H46N6NaO2	737.35745	C23H23N3O	0.45	-0.61	even	2M+Na

Figure S76: HRMS of 4-(dimethylamino)-*N*'-(1,2-diphenylethylidene)benzohydrazide (7f).

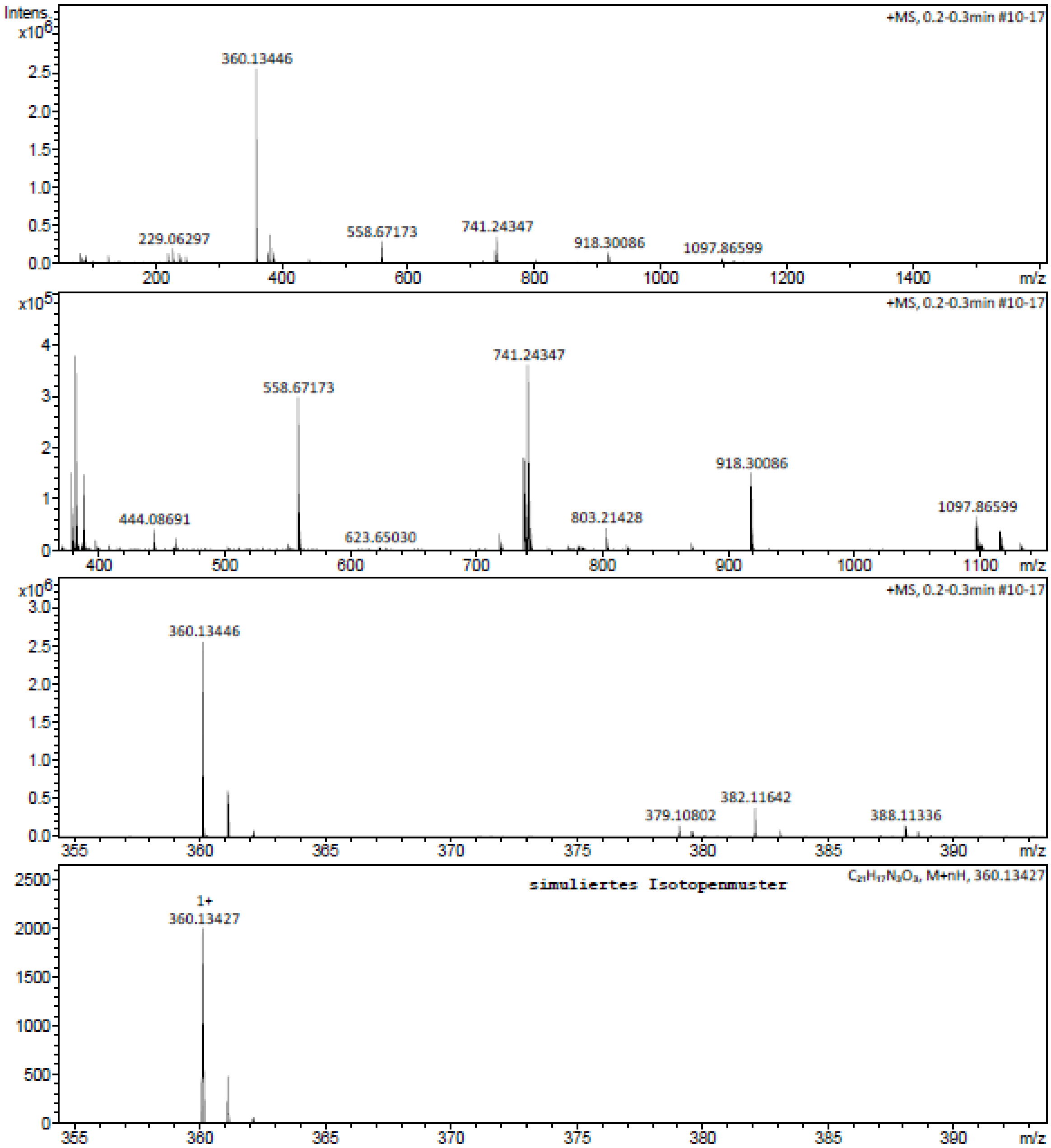

#	Meas. m/z	Ion Formula	m/z	Sum Formula	|err| [mDa]	err [ppm]	e⁻ Conf	Adduct
1	360.13446	C21H18N3O3	360.13427	C21H17N3O3	0.19	-0.53	even	M+H
1	382.11642	C21H17N3NaO3	382.11621	C21H17N3O3	0.21	-0.54	even	M+Na
1	741.24347	C42H34N6NaO6	741.24320	C21H17N3O3	0.26	-0.36	even	2M+Na

Figure S77: HRMS of 4-nitro-*N*'-(1,2-diphenylethylidene)benzohydrazide (7g).

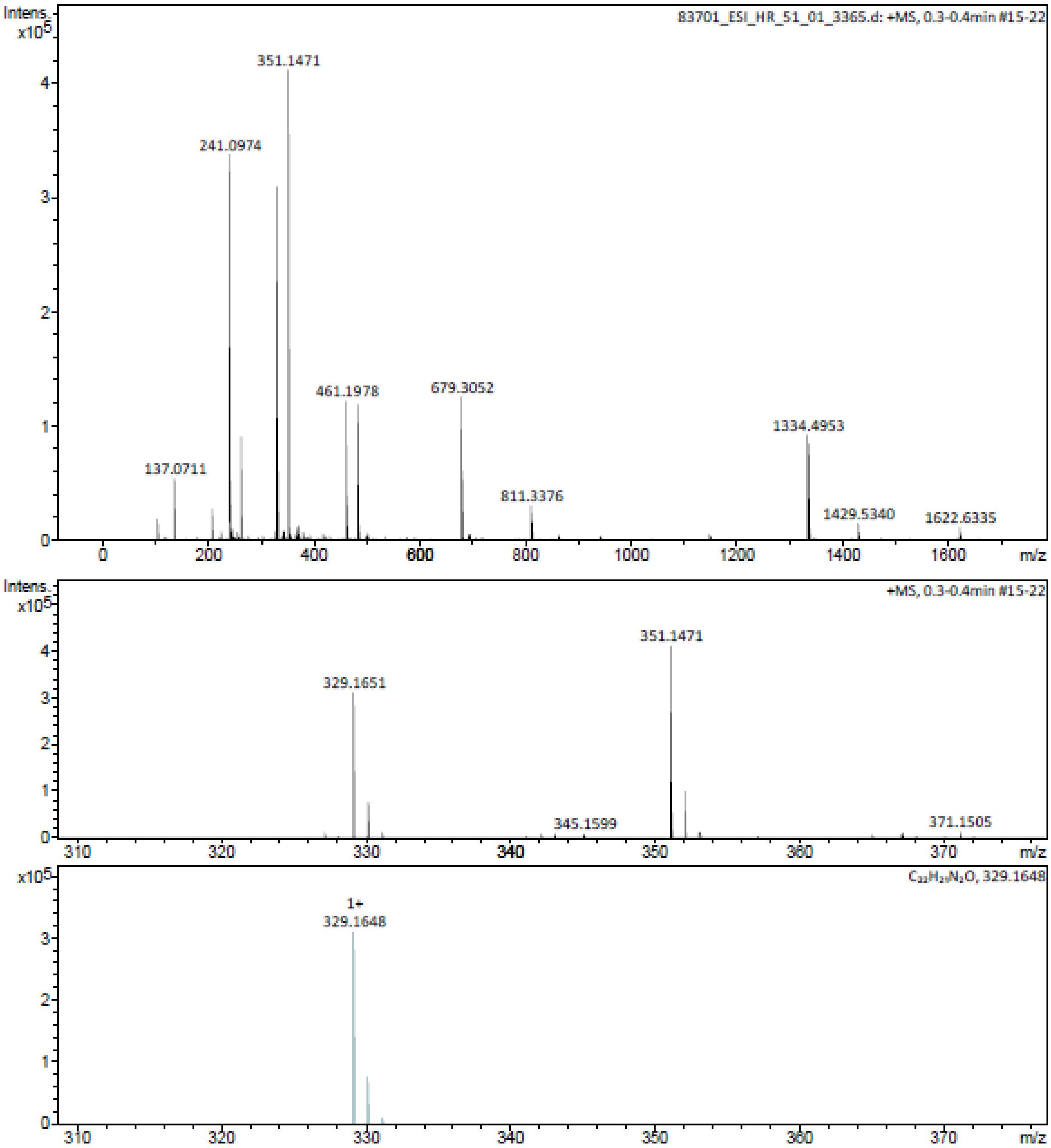

Meas. m/z	#	Ion Formula	m/z	err [ppm]	mSigma	# mSigma	Score	rdb	e⁻ Conf	N-Rule
329.1651	1	C22H21N2O	329.1648	-0.9	1.4	1	100.00	13.5	even	ok

Figure S78: HRMS of *N*'-(1-phenyl-2-(*o*-tolyl)ethylidene)benzohydrazide (7h).

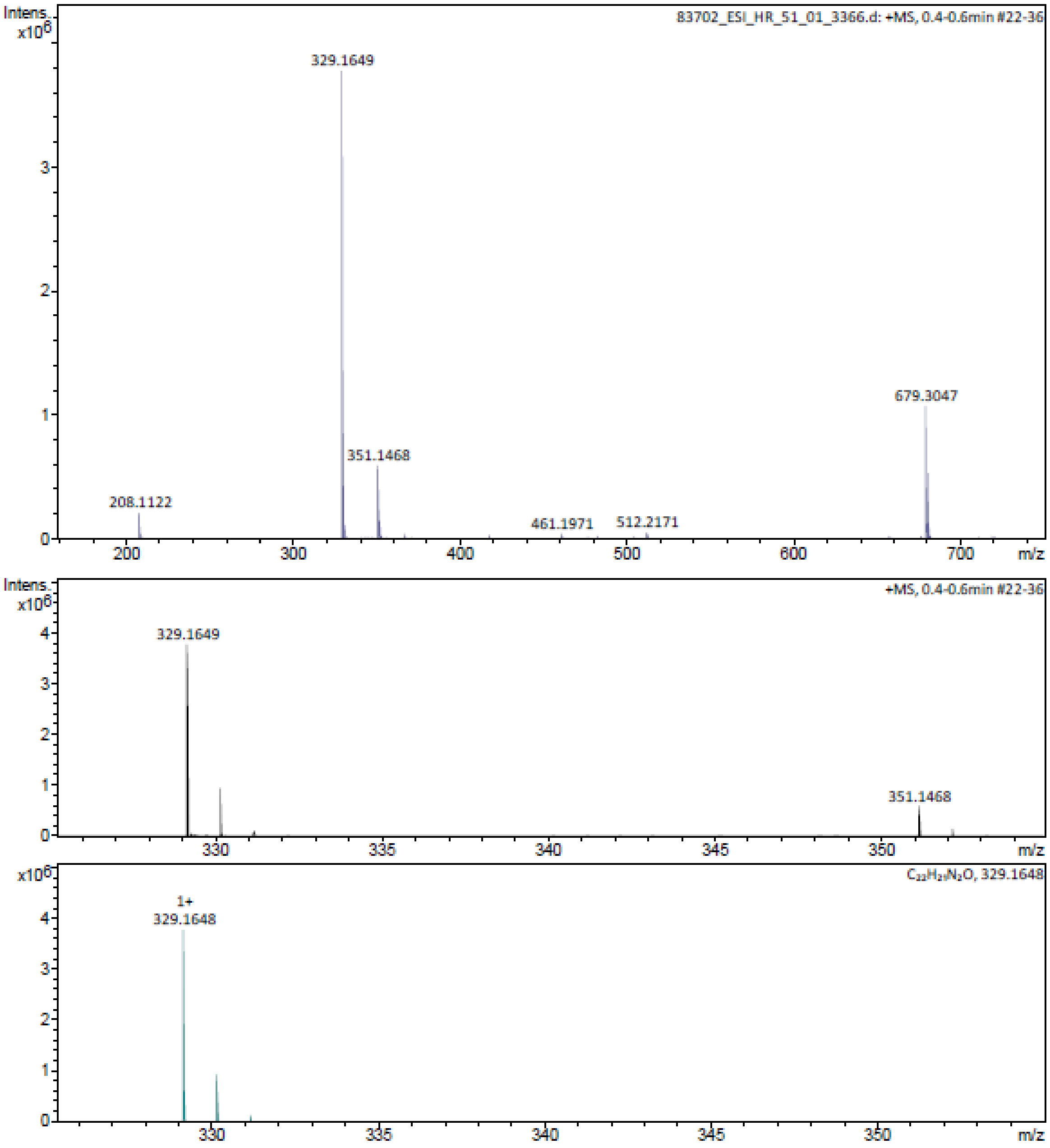

Meas. m/z	#	Ion Formula	m/z	err [ppm]	mSigma	# mSigma	Score	rdb	e⁻ Conf	N-Rule
329.1649	1	C22H21N2O	329.1648	-0.0	0.7	1	100.00	13.5	even	ok
351.1468	1	C22H20N2NaO	351.1468	-0.0	4.4	1	100.00	13.5	even	ok

Figure S79: HRMS of *N*'-(2-phenyl-1-(*m*-tolyl)ethylidene)benzohydrazide and *N*'-(1-phenyl-2-(*m*-tolyl)ethylidene)-benzohydrazide (7i).

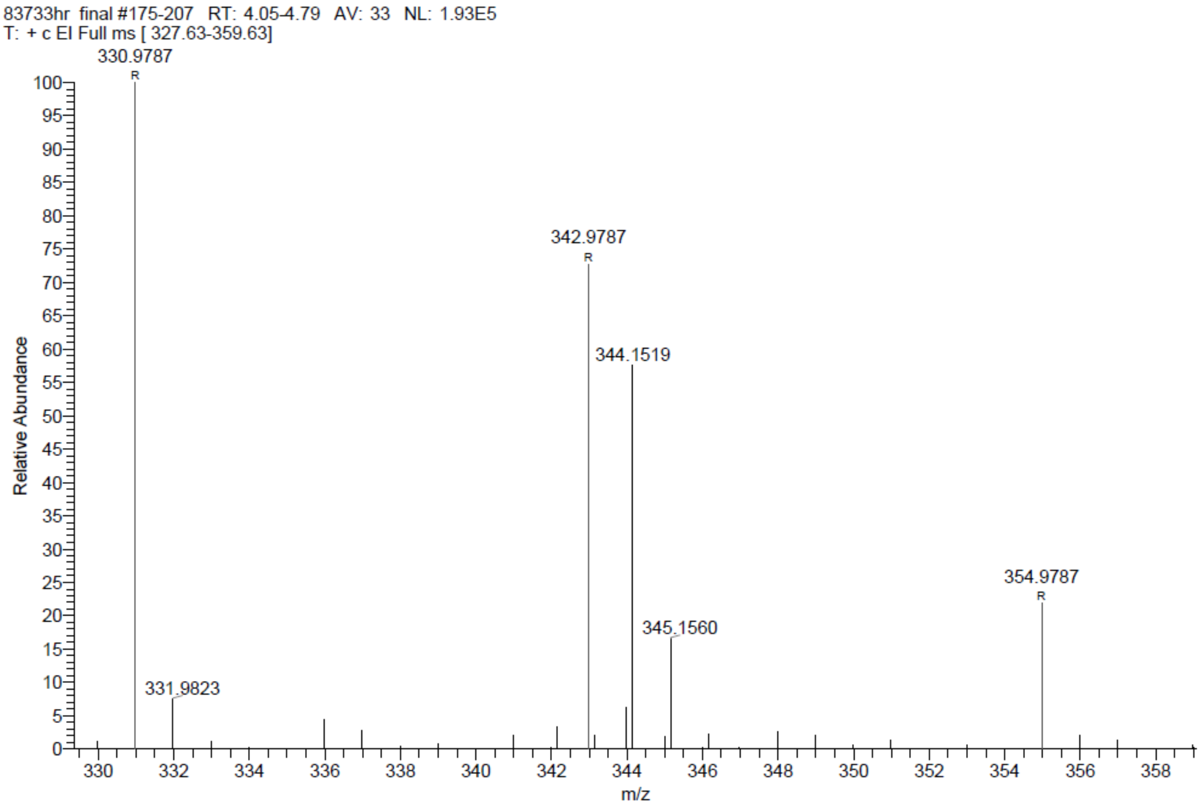

m/z= 339.1519-349.1519

m/z	Theo. Mass	Delta (mmu)	RDB equiv.	Composition
344.1519	344.1519	-0.05	14.0	$C_{22}H_{20}O_2N_2$
	344.1479	3.97	10.0	$C_{17}H_{20}O_4N_4$

Figure S80: HRMS of *N*'-(1-(3-methoxyphenyl)-2-phenylethylidene)benzohydrazide and *N*'-(2-(3-methoxyphenyl)-1-phenylethylidene)benzohydrazide (7j).

4. Gas chromatographic measurements

A chromatograph with a split/splitless injector system and a flame ionization detector was used. Chromatographic separation was performed by using a 15 m × 250 µm Varian CP-Sil 8 CB column (df = 1.0 µm) and nitrogen was used as carrier gas at a flow rate of 1.11 mL/min. All injections were carried out in the split flow mode with a split ratio of 7.6:1. The injector was maintained at a temperature of 200 °C and the detector at 270 °C. Quantification was accomplished by using mesitylene as internal standard. The concentration of the standard was equal to each of the substrates in the screening reactions.

The following ramping method was used for all substrate measurements: 3 min hold at 60 °C, 25°C/min heating to 300 °C, then hold at 300 °C for 6 min. (18.6 min total runtime).

Table S1: Retention times of all substrates and the internal standard

Compound class	Compound name	Retention time / min
Alkynes	1-Octyne	4.50
	Cyclohexylacetylene	4.79
	t-Butylacetylene	1.15
	Phenylacetylene	5.01
	Mesitylacetylene	7.81
	2,4,6-Triisopropylphenylacetylene	9.79
	4-Dimethylaminophenylacetylene	9.26
	4-Methoxylphenylacetylene	7.67
	4-Trifluormethylphenylacetylene	5.21
	1-Phenyl-1-butyne	7.70
	1-(Hex-1-yn-1-yl)-3-methoxybenzene	9.61
	1-(Hex-1-yn-1-yl)-3-(trifluoromethyl)benzene	9.81
	Diphenylacetylene	10.31
	1-Methyl-2-(phenylethynyl)benzene	10.72
	1-Methyl-3-(phenylethynyl)benzene	10.87
	1-Methyl-4-(phenylethynyl)benzene	10.92
	1-Methoxy-3-(phenylethynyl)benzene	11.30
Internal standard	Mesitylene	6.12

Benzhydrazides and benzhydrazones could not be observed via GC due to low volatility.

5. References

[1] S. G. Weber, F. Rominger, B. F. Straub, *Eur. J. Inorg.* Chem, **2012**, *17*, 2863–2867.

[2] S. G. Weber, D. Zahner, F. Rominger, B. F. Straub, *ChemCatChem*, **2013**, *5*, 2330–2335.

[3] D. P. Zimin, D. V. Dar'in, V. A. Rassadin, V Y. Kukushkin, *Org. Lett.,* **2018,** *20*, 4880–4884.

[4] R. Berger, K. Duff, J. L. Leighton, *J. Am. Chem. Soc.,* **2004**, 126(18), 5686–5687

[5] Y. Hu, Z. Zhang, J. Zhang, Y. Liu, I.D. Gridnev, W. Zhang, Angew. Chem. Int. Ed., **2019**, 58(44), 15767-15771
